# Dynamic alterations and potential roles of gut microbiota and metabolites in *Angiostrongylus cantonensis*-infected mice and rats

**DOI:** 10.1186/s40249-026-01436-7

**Published:** 2026-07-02

**Authors:** Yue Hu, Jian-Song Chen, Min-Yu Zhou, Hui Huang, Yun-Fei Zhou, Hai-Yun Zhou, Zhi-Yue Lv

**Affiliations:** 1 Key Laboratory of Tropical Disease Control (Sun Yat-sen University), Ministry of Education, Guangzhou, Guangdong China; 2 Provincial Engineering Technology Research Center for Biological Vector Control, Guangzhou, Guangdong China; 3 Guangdong MS Institute of Scientific Instrument Innovation, Guangzhou, China; 4 National Health Commission of the People’s Republic of China (NHC) Key Laboratory of Tropical Disease Control, Hainan Medical University, Haikou, Hainan China; 5 Instrumental Analysis and Research Center, Sun Yat-sen University, Guangzhou, China

**Keywords:** *Angiostrongylus cantonensis*, Gut microbiome, Metabolomics, Biomarker, Microbiota-gut-brain axis

## Abstract

**Background:**

Angiostrongyliasis, a food-borne parasitic disease caused by *Angiostrongylus cantonensis*, is characterized by eosinophilic meningitis or meningoencephalitis, leading to serious central nervous system damage. Current diagnostic methods lack specificity or sensitivity, and the pathogenesis is complex and incompletely understood. This study aimed to comprehensively characterize the dynamic alterations in the gut microbiota and host metabolism in both suitable (rats) and non-suitable (mice) hosts following *A. cantonensis* infection and to identify potential metabolic biomarkers for early diagnosis.

**Methods:**

Female BALB/c mice and Sprague Dawley rats (*n* = 10/group) were infected with 30 or 100 third-stage larvae, respectively. Serum, urine, feces, and brain samples were collected longitudinally. Gut microbiota was analyzed via *16S* rRNA gene sequencing and metagenomics. Host metabolism was profiled using untargeted and targeted metabolomics via ultraperformance liquid chromatography-quadrupoles/time of flight-mass spectrometry. Statistical analyses included Wilcoxon rank sum test, linear discriminant effect size analysis, Spearman correlation analysis, orthogonal partial least squares-discriminatory analysis, and receiver operating characteristic curve analysis.

**Results:**

Infection induced significant, host-specific gut microbiota dysbiosis. In infected hosts, Firmicutes decreased (*P* < 0.05) while Bacteroidetes increased (*P* < 0.05). A main difference in gut flora structure between infected hosts was observed in Prevotellaceae, which increased significantly in mice (*P* < 0.05) but decreased in rats (*P* < 0.05). Metagenomics revealed enhanced carbohydrate metabolism and fatty acid biosynthesis in gut microbes of infected mice, whereas up-regulated amino acid and vitamin metabolism were also observed in infected rats. Infection caused pronounced disruptions in host lipid and bile acid (BA) metabolism, changes in various BA types were closely related to alterations in specific bacterial genera (*P* < 0.05). Several metabolites, including phosphatidylcholine (16:0/18:1), 2-phenyl acetic acid, 2-octenoylglycine, lysophosphatidylcholine (18:2), O-glucuronide, and 2-carboxylic acid, were identified as potential early diagnostic biomarkers in the mouse model.

**Conclusions:**

*A. cantonensis* infection causes profound host-specific dysregulation of the gut microbiome and metabolome, with severe disturbances in Firmicutes, Bacteroidetes, lipid and BA metabolism being central features. These alterations highlight the critical role of the host-gut microbiota-metabolite axis in pathogenesis and offer novel insights for developing diagnostic and therapeutic strategies.

**Graphical abstract:**

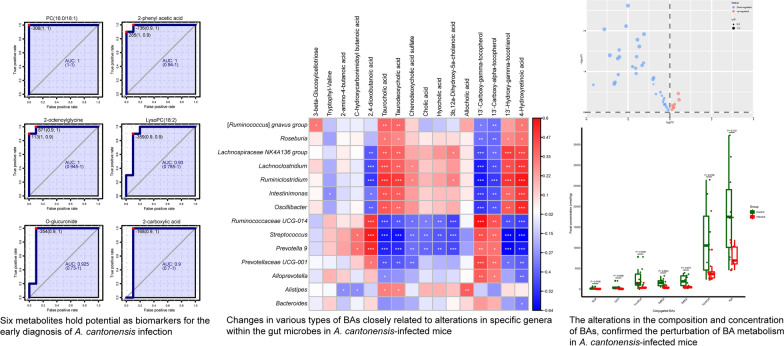

**Supplementary Information:**

The online version contains supplementary material available at 10.1186/s40249-026-01436-7.

## Background

*Angiostrongylus cantonensis* was first discovered in the pulmonary artery of *Rattus norvegicus* by Professor Chen in 1935 [1]. Since the first case of angiostrongyliasis was reported in Taiwan, China in 1945 [2], the endemic area of this disease has gradually expanded, resulting in an increased number of affected individuals. Angiostrongyliasis is mainly distributed in tropical and subtropical regions, particularly in Southeast Asia, the Pacific islands, and the Caribbean. However, recent global changes such as climate warming, environmental alterations, changes in dietary patterns, and increased international travel and cooperation have contributed to the spread of angiostrongyliasis in the Americas, including the United States, Caribbean islands, and Brazil [3–5]. Consequently, the World Health Organization (WHO) has declared angiostrongyliasis an emerging global threatening infectious disease in the twenty-first century [6]. As a result, there has been an increased focus on the prevalence and control of this disease among the general public.

The rat is a suitable host for *A. cantonensis*, whereas the mouse and human are non-suitable hosts. Upon ingestion of third-stage larvae (L3) from contaminated food, the larvae migrate to the central nervous system (CNS) in non-suitable hosts, causing severe mechanical injury and inflammation, but fail to complete their development. In contrast, in suitable hosts, larvae migrate to the pulmonary arteries and mature with a comparatively milder CNS inflammatory response [7]. Beyond direct parasitic damage, the host’s systemic response plays a crucial role in disease outcome. Research has demonstrated that *A. cantonensis* infection induces a Th2 immune response in the host, leading to increased eosinophil levels. These eosinophils further amplify and enhance the immune response [8–10], indicating their crucial role in the development and progression of angiostrongyliasis. Additionally, *A. cantonensis* infection upregulates the expression of IL-33 and activates the IL-33/ST2L pathway. IL-33, in turn, regulates eosinophils infiltration, Th2 cell differentiation, and the expression of IL-5 and IL-13 [11–13]. Eotaxin and Chi3l3 also play vital roles in eosinophils recruitment. The levels of Eotaxin and Chi3l3 are dramatically elevated in the cerebrospinal fluid (CSF) and peripheral blood of infected mice, while no significant changes in Chi3l3 were observed in infected rats [14–16]. Furthermore, significant differences in chemokines and miRNAs related to eosinophils increase were observed in the brains of infected rats and mice [17, 18]. Further studies have shown that microglia in the brains of infected mice become activated, a process related to the development of angiostrongyliasis [19]. Moreover, the apoptosis and necroptosis of microglia, astrocytes, and neurons in the brains of infected mice induce an inflammatory response in the CNS [20]. Nevertheless, although the induction of angiostrongyliasis involves multiple factors, its underlying mechanism remains complex and not fully understood.

Angiostrongyliasis typically presents as eosinophilic meningitis or meningoencephalitis, with a hallmark clinical feature being a marked elevation of eosinophils in CSF. Common clinical symptoms and signs include acute and severe headache, stiff neck, nausea and vomiting, paresthesia, cognitive dysfunction, and even coma or epilepsy [3, 21, 22]. The gold standard for diagnosis is parasitological examination, which detects larvae in the CSF, ocular tissues, or other affected tissues. Unfortunately, the detection rate is approximately 10% [21, 22]. In addition, immunological examinations, including enzyme linked immunosorbent assay (ELISA), radiological examinations such as computed tomography (CT) and magnetic resonance imaging (MRI), and molecular diagnostics like polymerase chain reaction (PCR), real-time quantitative polymerase chain reaction (RT-qPCR), and loop-mediated isothermal amplification (LAMP) are also employed for adjunctive diagnosis of angiostrongyliasis [21–25]. While these methods have been developed, issues with cross-reactivity, sensitivity, and accessibility limit their utility for early and definitive diagnosis. Consequently, there is a pressing need for novel diagnostic biomarkers.

Recent research has highlighted the significance of the gut-brain axis—a bidirectional communication network linking the enteric nervous system (ENS) and the CNS via neural, immunological, neuroendocrinal, and metabolic pathways. The gut microbiota, a key component of this axis, modulates host immunity, metabolism, and even brain function [26]. Previous studies have shown that imbalances in the gut microbiota occur in patients with autism spectrum disorders (ASD), Alzheimer's disease (AD), and Parkinson's disease (PD); these imbalances increase the permeability of the intestinal mucosal barrier and the blood–brain barrier (BBB), leading to a series of inflammatory responses [27–29]. Patients with angiostrongyliasis often exhibit inflammation of the CNS and cognitive dysfunction, but so far, it remains uncertain whether the gut microbiota mediates the occurrence and development of the disease.

Given that *A. cantonensis* infection induces a strong systemic Th2 immune response and CNS inflammation, and considering the gut's role as a primary interface with the parasite during initial infection, we hypothesized that the gut microbiota and its associated metabolites are dynamically altered during infection and may contribute to the differential pathogenesis observed in suitable versus non-suitable hosts. Furthermore, such systemic metabolic alterations may yield biomarkers detectable in biofluids.

To test this hypothesis, we employed a multi-omics approach, integrating *16S* rRNA gene sequencing, metagenomics, and untargeted/targeted metabolomics to longitudinally profile the gut microbiota and host metabolism in both *A. cantonensis*-infected mice (non-suitable host) and rats (suitable host). This comparative study aims to: (1) elucidate the dynamic changes in gut microbial community structure and function; (2) characterize the systemic metabolic perturbations induced by infection; (3) explore correlations between specific microbial shifts and metabolic changes; and (4) identify potential metabolic biomarkers for early diagnosis. These findings will provide a better understanding of the mechanisms involved in the development of angiostrongyliasis.

## Methods

### Animals and parasite

Specific-pathogen-free, 6-week-old female BALB/c mice (18–20 g body weight) and Sprague Dawley (SD) rats (180–230 g body weight) were purchased from Charles River Laboratories (Beijing, China) and housed in plastic cages. The animals had ad libitum access to autoclaved chow and water, and were kept under controlled temperature and humidity conditions. After a one-week acclimation period, animals were randomly assigned to control or infected groups (*n* = 10 per group per time point). For the infection model, each mouse or rat was infected with either 30 or 100 L3 of *A. cantonensis* via intragastric administration [16]. The L3 of *A. cantonensis* were obtained from infected *Biomphalaria glabrata* by digesting minced snail tissue in a pepsin-HCl solution and incubating for 40 min at 37 °C. Subsequently, an appropriate volume of PBS was added to terminate digestion, and the L3 were counted under a stereoscopic microscope (SZ650, Cnoptec, Chongqing, China).

### Experimental design and sample collection

A schematic workflow of the experimental design is presented in Additional file 1. Body weight was monitored throughout the experiment as a general health indicator. At least 0.5 ml of urine and 1 g of feces were collected from metabolic cages under low temperature conditions one day before animal sacrifice. Subsequently, healthy and *A. cantonensis*-infected animals were anesthetized at the corresponding time points (1, 3, 7, 14, 21, 28, and 35 days) using intraperitoneal injection of pentobarbital sodium. Blood was collected from the orbital veins, and serum was obtained by centrifugation at 3000 × *g* for 10 min. Finally, the animals were euthanized by cervical dislocation, and their whole brains were dissected on ice. All samples were immediately flash-frozen in liquid nitrogen and stored at −80 °C until analysis.

### DNA extraction, *16S* rRNA gene sequencing and analysis

Genomic DNA was extracted from fecal samples collected from mice and rats in the control and infected groups (*n* = 5 for each group). The extracted DNA was used as a template for amplifying the V3–V4 regions of the *16S* rRNA gene, and the resulting amplicons were purified and pooled for subsequent *16S* rRNA sequencing as described previously [30]. Quality control of sequencing data, clustering, and annotation of operational taxonomic units (OTUs) were performed as previously mentioned [30].

To assess species level differences, several alpha diversity indices were calculated, including the Chao1 index, observed species index, Shannon index, and Simpson index. These indices allow for the assessment of species richness and evenness within the bacterial community. Meanwhile, beta diversity analysis was applied to compare the differences in community structure among different groups, using principal coordinate analysis (PCoA) for visual representation. To distinguish significant differences in bacterial communities between healthy animals and *A. cantonensis*-infected animals at each time point, Wilcoxon rank sum test was performed at various taxonomic levels. The resulting *P*-values were adjusted for multiple comparisons using the Benjamini–Hochberg false discovery rate (FDR) correction. A corrected *P*-value < 0.05 was considered statistically significant. In addition, biomarkers distinguishing groups were identified using linear discriminant effect size (LEfSe) analysis with a linear discriminant analysis (LDA) score threshold > 3.0. Spearman correlation coefficients were calculated to explore potential relationships between the top 15 dominant gut bacteria at the genus level, and Cytoscape 3.8.2 software (Cytoscape Consortium, San Diego, CA, USA) was utilized for visualization. Unless otherwise stated, all statistical analyses and data visualizations were performed using R 4.0.3 software (R Foundation for Statistical Computing, Vienna, Austria).

### Metagenome sequencing and analysis

DNA extraction was performed on fecal samples from control and infected animals at selected time points (mice: day 21; rats: days 21 and 35), with five samples per group. The DNA was fragmented, a library was constructed, bridge PCR was performed, and the resulting DNA was then pooled and sequenced according to the previous protocol [30]. Detailed bioinformatics parameters are provided in Additional file 2.

### Sample processing for untargeted metabolomics

Serum (10 μl) was mixed with 90 μl of prechilled methanol, vortexed, incubated overnight at 4 °C, and centrifuged at 10,000 × *g* for 10 min at 4 °C. The supernatant was transferred to a vial for testing. Urine (50 μl) was diluted at a ratio of 1∶10 with precooled Milli-Q water and centrifuged at 10,000 × *g* for 10 min at 4 °C. Subsequently, the supernatant was transferred to a vial. Feces (50 mg) were mixed with 1 ml of precooled methanol, vortexed and centrifuged twice at 13,000 × *g* for 10 min at 4 °C, the supernatant was then transferred into a vial for further analysis. Brain tissue (50 ± 0.5 mg) was homogenized at low temperature in 1.5 ml of prechilled methanol/water (1∶1, v∶v) containing magnetic beads. The suspension was centrifuged at 16,000 × *g* for 10 min at 4 °C, then the resulting supernatant was discarded, and the sediment was homogenized in 1.6 ml of precooled dichloromethane/methanol (3∶1, v∶v) solvent, followed by centrifugation under the same condition. The supernatant was transferred to a new Eppendorf tube, redissolved in 120 μl of precooled methanol/water (1∶1, v∶v) after drying, and centrifuged for 10 min at 13,000 × *g* to remove particulates. Subsequently, the supernatant was transferred to a sampling vial. To ensure the stability and repeatability of the analysis, quality control (QC) samples were prepared by mixing equal volumes of all experimental samples.

### Data analysis of untargeted metabolomics

Detailed conditions of ultraperformance liquid chromatography-quadrupoles/time of flight-mass spectrometry (UPLC-Q/TOF-MS) are provided in Additional file 2. Masslynx 4.1 (Waters, Milford, MA, USA) was applied to acquire the raw data, which were then imported into Progenesis QI 2.1 (Waters, Milford, MA, USA) for data preprocessing, including peak alignment, picking, and normalization. Peak intensities were normalized to the pooled QC sample, providing relative abundance data for each metabolite feature across samples. Subsequently, multivariate statistical analyses were performed with SIMCA-P 14.0 (Umetrics AB, Umea, Sweden) and MetaboAnalyst 4.0 [31]. These analyses included principal component analysis (PCA) and orthogonal partial least squares-discriminatory analysis (OPLS-DA). The explanatory power, predictive ability, and goodness of fit of the OPLS-DA models were assessed using the quality parameters (R^2^Y and Q^2^), and validated by permutation tests (100 iterations) to guard against overfitting. Metabolites deemed discriminative were selected based on criteria: variable importance in the projection (VIP) > 2.0 or 1.0 in the OPLS-DA model and *P* < 0.05 in an independent-samples t test (with Benjamini–Hochberg FDR correction). The accurate mass spectral data and MS/MS spectra were compared with the Human Metabolome Database (HMDB) [32] and LipidMaps database [33] for putative identification of differential metabolites. Furthermore, MetaboAnalyst 4.0 [31] was used to perform receiver operating characteristic (ROC) curve analysis to evaluate the early diagnostic capability of the identified potential biomarkers. Additionally, pathway analysis was performed to investigate the primary significant metabolic pathways involved in *A. cantonensis* infection. Finally, R 4.0.3 software (R Foundation for Statistical Computing, Vienna, Austria) was applied to calculate correlations between differential gut microbiota and differential fecal metabolites.

### Targeted metabolomics analysis of bile acids (BAs) in feces

Targeted metabolomics analysis was conducted to quantify the absolute concentrations of BAs in fecal samples. A 25 mg aliquot of feces was mixed with 1 ml of prechilled acetonitrile/methanol/water (2∶2∶1, v∶v, containing 0.1% formic acid and isotopically-labelled internal standard mixture), vortexed for 30 s, homogenized for 4 min at 35 Hz, sonicated for 5 min in an ice-water bath thrice, followed by incubation for 1 h at −40 °C. Subsequently, the clear supernatant was obtained after centrifugation at 13,000 × *g* for 15 min at 4 °C and transferred to an auto-sampler vial.

Feces samples and BA reference standards were separated using Vanquish ultra-high pressure liquid chromatography (Thermo Fisher Scientific, Waltham, MA, USA), equipped with an ACQUITY UPLC C18 BEH column (2.1 × 150 mm, 1.7 μm; Waters, Milford, MA, USA). The mobile phase A consisted of 1 mmol/L ammonium acetate and 0.1% acetic acid in water, while the mobile phase B comprised acetonitrile. All samples were injected at 50 °C in a random order with an injection volume of 1 μl.

A Q Exactive HFX mass spectrometer (Thermo Fisher Scientific, Waltham, MA, USA) was operated with spray voltages of 3.5 kV (positive polarity) and 3.1 kV (negative polarity), respectively. The sheath gas and auxiliary gas were set to 40 psi and 15 psi, respectively. The auxiliary gas temperature was maintained at 350 °C, and the capillary temperature at 320 °C. Mass spectrometry analysis was conducted in a parallel reaction monitoring (PRM) pattern, with optimized PRM parameters for each targeted analyte. Since most BAs lacked suitable product ions for quantification, the precursor ion in high resolution was selected for quantification. Raw data were analyzed using Xcalibur 3.0 software (Thermo Fisher Scientific, Waltham, MA, USA) to obtain calibration equations and the quantitative concentration of each analyte in the samples. Univariate and multivariate statistical analyses were carried out as described for untargeted metabolomics.

## Results

### Community structure of gut microbiota in *A. cantonensis*-infected mice and rats

High-throughput sequencing of the *16S* rRNA gene was performed to assess the impact of infection on the gut microbiota. A total of 9233 and 8923 OTUs at a 97% similarity level were obtained from mouse and rat fecal samples for subsequent analysis, respectively. No significant differences in alpha diversity indices were observed between infected and control groups for either species at any time point (*P* > 0.05), indicating comparable richness and evenness of gut flora between the two groups (Additional files 4 and 5).

However, beta diversity analysis revealed significant structural alterations. In mice, three-dimensional PCoA plots based on weighted UniFrac distances (Additional file 6) showed clear separation between infected and control groups at early time points (days 1, 3, and 7; Adonis analysis, *P* < 0.05), but the two groups relatively converged at later stages (days 14 and 21; Adonis analysis, *P* > 0.05). In rats, the community structure of infected and control groups significantly differed from day 1 through day 28 (Adonis analysis, *P* < 0.05), but showed no significant difference at day 35 (Adonis analysis, *P* > 0.05) (Additional file 7).

**Fig. 1:**
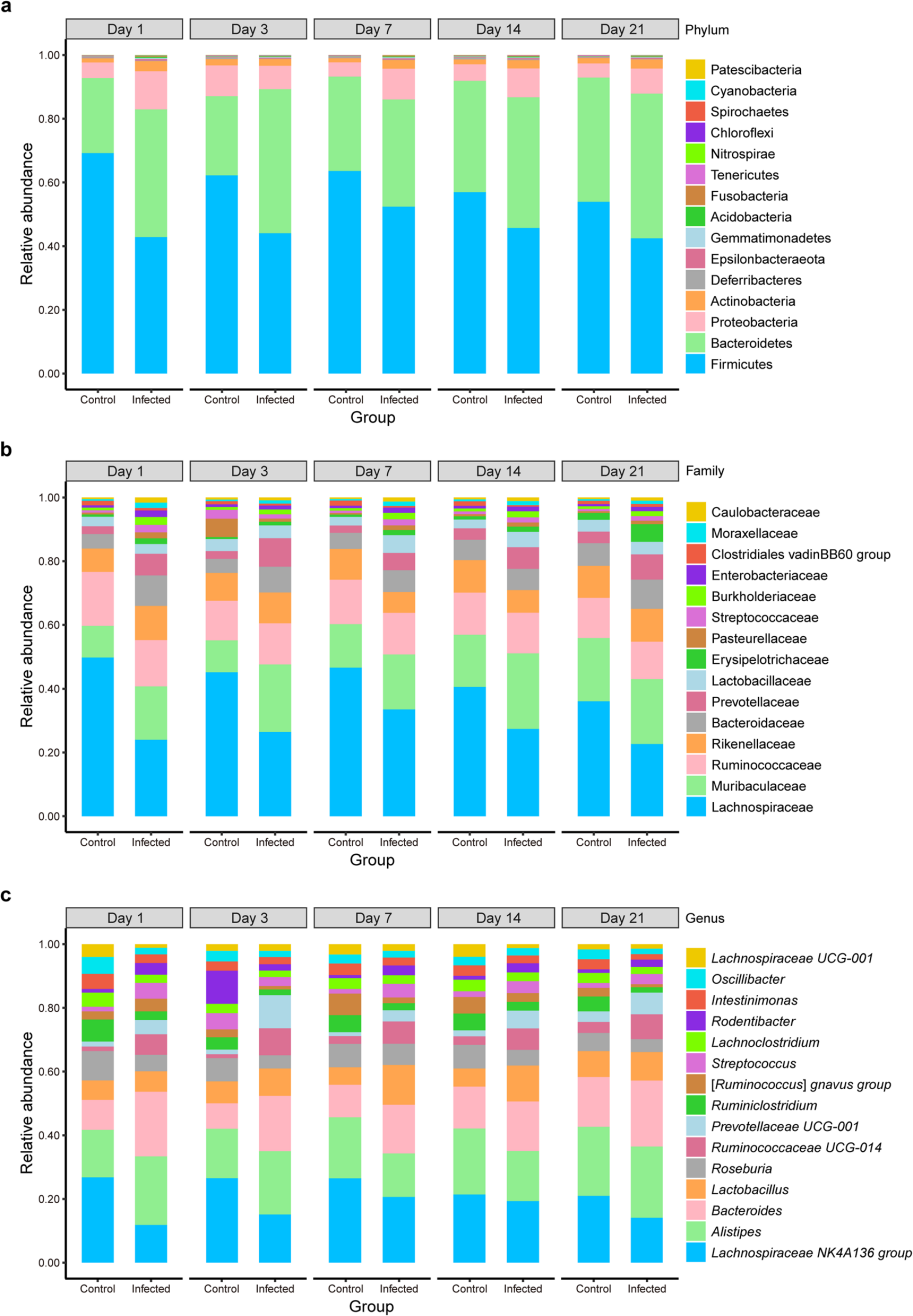
Relative abundances of the 15 most important gut microbiota constituents of control mice and *Angiostrongylus cantonensis*-infected mice at the phylum (**a**), family (**b**) and genus (**c**) levels across different time points as assessed by *16S* rRNA sequencing. Each column represents the composition of the microbial taxa in one group

To assess temporal changes in gut microbiota during *A. cantonensis* infection, bar plots were generated to depict the relative abundances of the top 15 dominant bacterial taxa. At the phylum level, fecal bacteria from both infected and control mice were predominantly classified into four phyla: Firmicutes, Bacteroidetes, Proteobacteria, and Actinobacteria, together accounting for over 98% of the total community (Fig. 1a). Lachnospiraceae, Muribaculaceae, Ruminococcaceae, Rikenellaceae, Bacteroidaceae, and Prevotellaceae had relatively high abundances at the family level (Fig. 1b), all belonging to Firmicutes or Bacteroidetes. At the genus level, *Lachnospiraceae NK4A136 group*, *Alistipes*, *Bacteroides*, *Lactobacillus*, *Roseburia*, *Prevotellaceae UCG-001*, etc., were the dominant bacteria in both infected and control mouse fecal samples (Fig. 1c), accounting for average relative abundances greater than 1%.

**Fig. 2:**
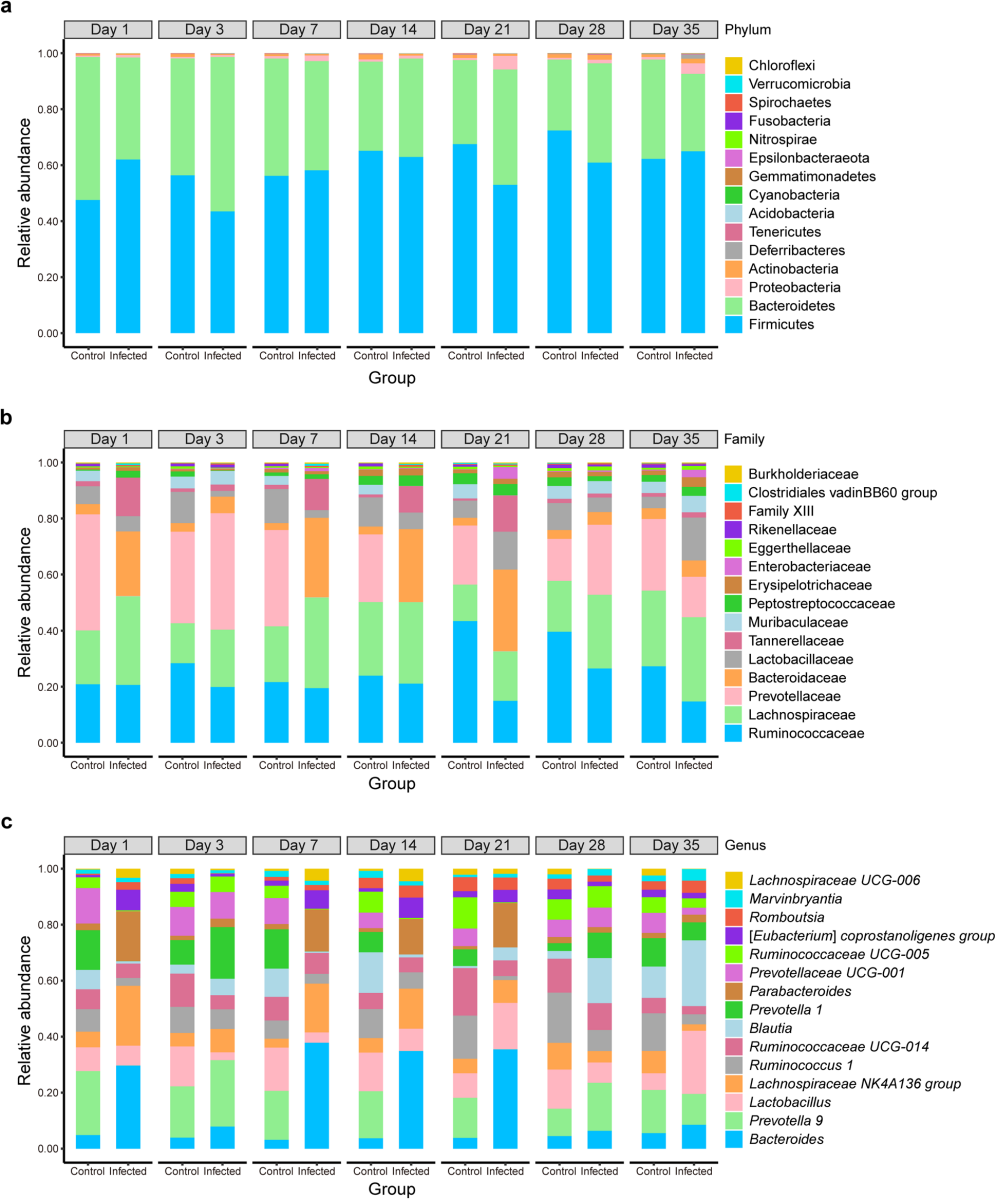
Relative abundances of the 15 most important gut microbiota constituents of control rats and *Angiostrongylus cantonensis*-infected rats at the phylum (**a**), family (**b**) and genus (**c**) levels across different time points as assessed by *16S* rRNA sequencing. Each column represents the composition of the microbial taxa in one group

In both infected and control rats, Firmicutes, Bacteroidetes, Proteobacteria, and Actinobacteria were the dominant phyla, collectively accounting for over 98% in samples of each group (Fig. 2a). At the family level, Ruminococcaceae, Lachnospiraceae, Prevotellaceae, Bacteroidaceae, and Lactobacillaceae, belonging to Firmicutes or Bacteroidetes, exhibited higher relative abundances in all groups (Fig. 2b). The top 15 genera in the samples included *Bacteroides*, *Prevotella 9*, *Lactobacillus*, *Lachnospiraceae NK4A136 group*, *Ruminococcus 1*, *Ruminococcaceae UCG-014*, etc., with average relative abundances exceeding 1% (Fig. 2c).

### Infection induced dynamic shifts in gut bacteria of mice

In *A. cantonensis*-infected mice, the relative abundance of Firmicutes decreased significantly at days 1 and 7 (*P* < 0.05), whereas Bacteroidetes increased dramatically at days 1 and 3 (*P* < 0.05). However, no significant difference was observed in the ratio of Firmicutes to Bacteroidetes between infected and control groups (*P* > 0.05). Proteobacteria was enriched in feces at days 1, 7, 14, and 21 (*P* < 0.05), and Actinobacteria was also enriched at 1 and 7 days post-infection (dpi) (*P* < 0.05). Furthermore, Epsilonbacteraeota abundance was up-regulated markedly in infected groups at days 1, 7, and 21 (*P* < 0.05) (Additional file 8).

Muribaculaceae and Bacteroidaceae, members of Bacteroidetes, were markedly increased in infected mice at days 1 and 3 (*P* < 0.05), and Prevotellaceae abundance increased significantly at days 1, 3, 7, 14, and 21 (*P* < 0.05). However, Rikenellaceae abundance decreased in infected mice at day 7 (*P* < 0.05). Lachnospiraceae was reduced at 1 and 7 dpi (*P* < 0.05), whereas Streptococcaceae increased at days 1, 7, and 14 (*P* < 0.05), Lactobacillaceae was significantly enriched at day 7 (*P* < 0.01); these families belong to Firmicutes. In addition, Burkholderiaceae and Enterobacteriaceae, members of Proteobacteria, were dramatically up-regulated at 1 and 7 dpi (*P* < 0.01) (Additional file 9).

In infected mice, major genera within Lachnospiraceae exhibited variable changes compared to controls. Specifically, *Lachnospiraceae NK4A136 group* and *Lachnoclostridium* down-regulated at 1 and 7 dpi (*P* < 0.05), *Roseburia* and [*Ruminococcus*] *gnavus group* were notably reduced in infected mice at days 1 and 7, respectively (*P* < 0.01), while *Lactobacillus* was more abundant at day 7 (*P* < 0.05). Although overall Ruminococcaceae abundance did not differ, *Ruminiclostridium* and *Oscillibacter*, both belonging to Ruminococcaceae, significantly decreased at 1, 7, and 21 dpi (*P* < 0.05), *Intestinimonas* declined at day 1 (*P* < 0.01), whereas *Ruminococcaceae UCG-014* increased at 1, 3, and 7 dpi (*P* < 0.05). Within Streptococcaceae, *Streptococcus* was noticeably enriched in infected mice at days 1, 7, 14, and 21 (*P* < 0.05), *Prevotellaceae UCG-001* was markedly up-regulated at 1 and 3 dpi (*P* < 0.05). Interestingly, among Bacteroidaceae and Rikenellaceae, *Bacteroides* only increased at 3 dpi (*P* < 0.05), whereas *Alistipes* decreased at 7 dpi (*P* < 0.05) (Additional file 10).

**Fig. 3:**
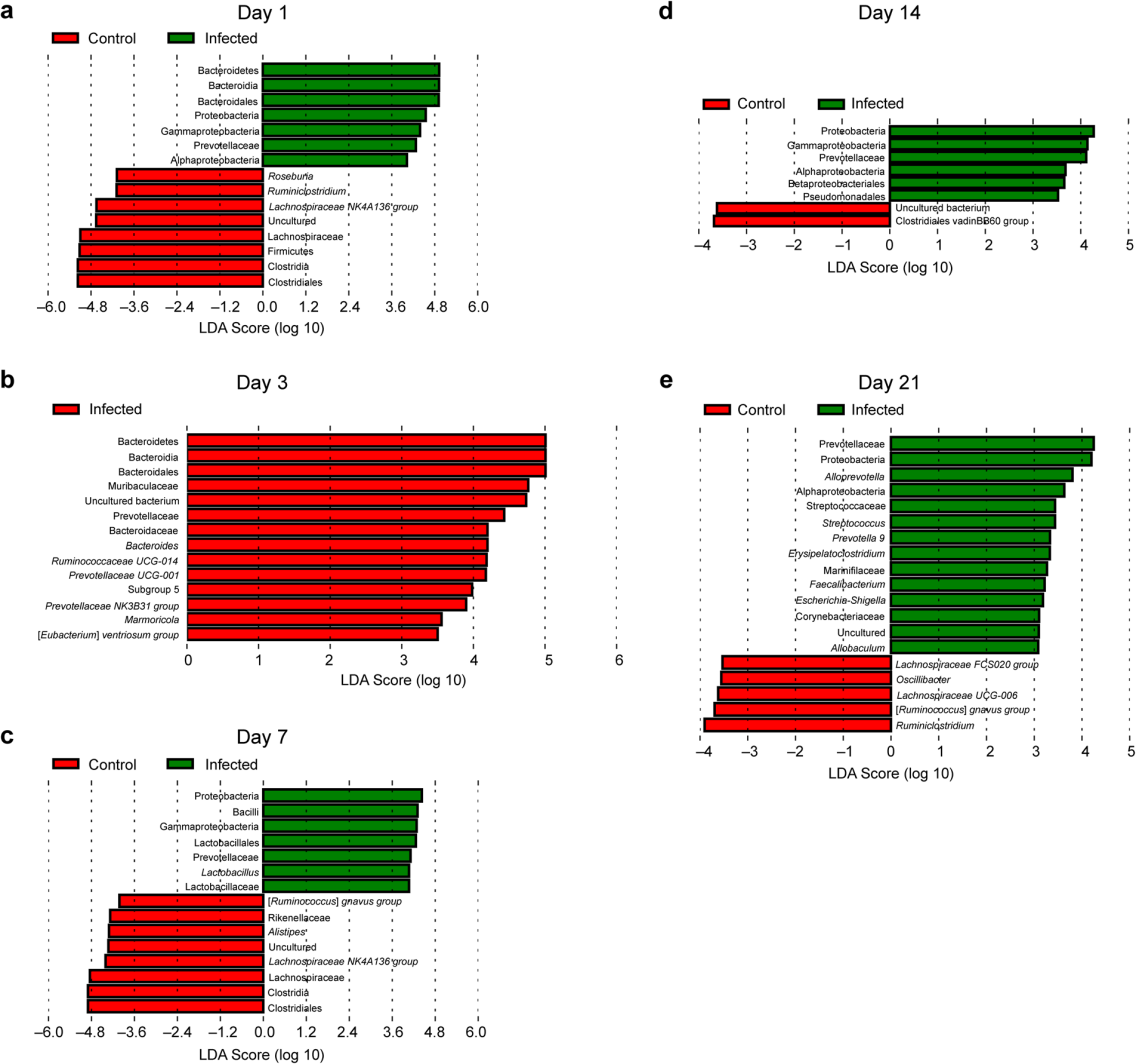
LEfSe analysis of gut microbiome between control mice and *Angiostrongylus cantonensis*-infected mice. **a** Day 1. **b** Day 3. **c** Day 7. **d** Day 14. **e** Day 21. Taxa highlighted in different colors indicate over-representation in the corresponding groups. *LEfSe* linear discriminant effect size

Subsequently, LEfSe analysis was applied to identify the microbial taxa distinguishing infected and control groups (Fig. 3). At 1 dpi, Gammaproteobacteria, Alphaproteobacteria, Bacteroidales, and Prevotellaceae were enriched in infected mice. At 3 dpi, key taxa included Muribaculaceae, Prevotellaceae, *Prevotellaceae UCG-001*, Bacteroidaceae, *Bacteroides*, and *Ruminococcaceae UCG-014*. By day 7, Gammaproteobacteria, Prevotellaceae, and *Lactobacillus* were key taxa. At day 14, Gammaproteobacteria, Alphaproteobacteria, Betaproteobacteriales, and Prevotellaceae were enriched in infected mice, whereas at day 21, Alphaproteobacteria, Prevotellaceae, *Prevotella 9*, *Alloprevotella*, and *Streptococcus* were notably up-regulated. Overall, Prevotellaceae consistently exhibited higher abundance in the intestinal flora of *A. cantonensis*-infected mice across multiple time points (LDA score > 4.0, *P* < 0.05), indicating its important role in the development of *A. cantonensis* infection and its potential as a diagnostic marker.

### Infection induced dynamic shifts in gut bacteria of rats

Additional file 11 demonstrated that the relative abundances of Firmicutes and Proteobacteria in feces of *A. cantonensis*-infected rats at day 1 were increased significantly (*P* < 0.05), while Bacteroidetes exhibited a marked decrease (*P* < 0.05). In contrast, Firmicutes and Actinobacteria were dramatically reduced at 3 dpi (*P* < 0.01), while Bacteroidetes exhibit a significant increase (*P* < 0.01). Similarly, down-regulation of Actinobacteria was observed at 14 dpi (*P* < 0.01), Firmicutes decreased at 21 and 28 dpi (*P* < 0.05), while Bacteroidetes and Proteobacteria exhibit higher abundances at 28 dpi (*P* < 0.05). Nevertheless, no marked differences were observed in the ratio of Firmicutes to Bacteroidetes between infected and control groups at different time points (*P* > 0.05). Moreover, Tenericutes showed a consistent downward trend in infected groups at days 1, 7, 14, and 21 (*P* < 0.01).

At the family level, Ruminococcaceae, classified within Firmicutes, was obviously down-regulated at 21, 28, and 35 dpi (*P* < 0.01), while the relative abundance of Lachnospiraceae was markedly up-regulated at 3 and 7 dpi (*P* < 0.05). Furthermore, Peptostreptococcaceae showed an increase at day 1 (*P* < 0.01) followed by a decrease at day 3 (*P* < 0.01). In addition, Lactobacillaceae and Erysipelotrichaceae exhibited decreased abundance in the infected group at day 3 (*P* < 0.05). Contrastingly, Prevotellaceae, belonging to Bacteroidetes, showed a significant decreased at 1, 7, 14, and 21 dpi (*P* < 0.01), but exhibited a marked increase at 3 and 28 dpi (*P* < 0.05). The relative abundance of Bacteroidaceae was notably increased at day 1, 3, 7, 14, and 21 dpi (*P* < 0.05). Interestingly, Muribaculaceae showed a significant decrease at days 1, 7, 14, and 21 (*P* < 0.01), while Tannerellaceae exhibited an increase during the same period (*P* < 0.01) (Additional file 12).

At the genus level, *Ruminococcaceae UCG-005* was less abundant in infected rats at days 1, 7, 14, and 21 (*P* < 0.01), whereas [*Eubacterium*] *coprostanoligenes group*, also belonging to Ruminococcaceae, was more abundant during the same period (*P* < 0.01). In addition, *Ruminococcus 1* (family Ruminococcaceae) had reduced abundance at 21, 28, and 35 dpi (*P* < 0.05), and *Ruminococcaceae UCG-014* decreased at days 3 and 21 (*P* < 0.05). *Lachnospiraceae NK4A136 group* was up-regulated at 7 and 14 dpi (*P* < 0.01), but down-regulated at 35 dpi (*P* < 0.05). Nevertheless, *Lachnospiraceae UCG-006* was enriched in infected rats at days 1, 7, and 14 (*P* < 0.05). *Blautia* (family Lachnospiraceae) exhibited lower abundance at 1, 7, and 14 dpi (*P* < 0.05), but higher abundance at day 28 (*P* < 0.05). A significant reduction was observed in *Prevotella 9* (family Prevotellaceae) at days 1, 7, 14, and 21 (*P* < 0.01). *Prevotella 1* (family Prevotellaceae) showed the same pattern (*P* < 0.01), except for a significant increase at 3 dpi (*P* < 0.05). The relative abundance of *Prevotellaceae UCG-001* was markedly decreased at days 1, 7, 14, 21, and 35 (*P* < 0.01). Marked increases were observed in *Bacteroides* (family Bacteroidaceae) at 1, 3, 7, 14, and 21 dpi (*P* < 0.05), which corresponded to the changes in Bacteroidaceae. Similarly, *Parabacteroides* (family Tannerellaceae) was enriched in infected groups at days 1, 7, 14, and 21 (*P* < 0.01), corresponding to the changes observed in Tannerellaceae (Additional file 13).

In summary, these results revealed that infection induced dynamic, time-dependent shifts in intestinal microbial composition in mice and rats. A key finding was the divergent response of the family Prevotellaceae (phylum Bacteroidetes). Its relative abundance was significantly increased in infected mice across multiple time points (*P* < 0.05), whereas it was generally decreased in infected rats (*P* < 0.01). Nevertheless, it is important to note that the baseline gut microbiota composition differed between mice and rats. Prior to infection, rat fecal samples showed a significantly higher relative abundance of *Prevotellaceae UCG-001 and Prevotella 9* compared to mice (*P* < 0.01) (Additional file 14), which may contribute to the divergent responses of these taxa to infection observed later.

**Fig. 4:**
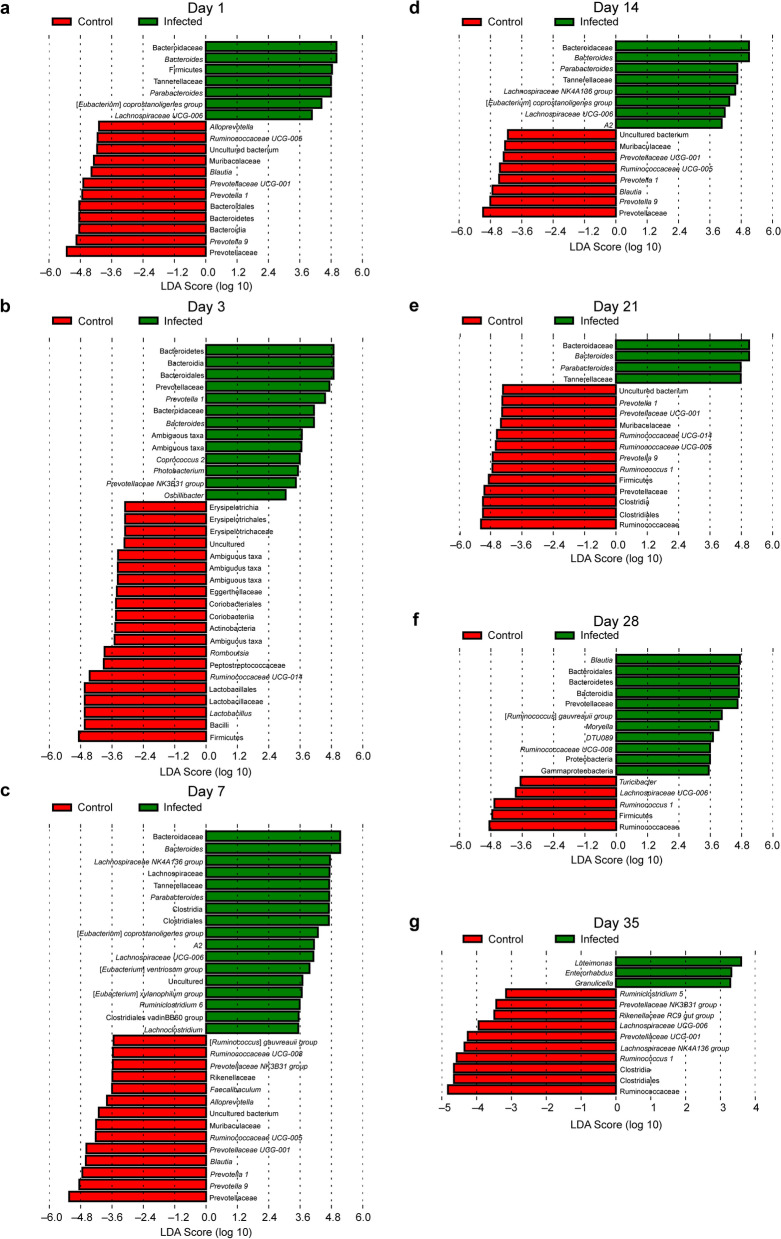
LEfSe analysis of gut microbiome between control rats and *Angiostrongylus cantonensis*-infected rats. **a** Day 1. **b** Day 3. **c** Day 7. **d** Day 14. **e** Day 21. **f** Day 28. **g** Day 35. Taxa highlighted in different colors indicate over-representation in the corresponding groups. *LEfSe* linear discriminant effect size

The results of LEfSe analysis indicated that *Bacteroides* could serve as a potential biomarker distinguishing infected groups from control groups at days 1, 3, 7, 14, and 21 (LDA score > 4.0, *P* < 0.05). Additionally, *Parabacteroides* and Tannerellaceae were capable of differentiating between infected and control groups at days 1, 7, 14, and 21. The relative abundance of Prevotellaceae notably increased in infected groups at days 3 and 28. *Blautia* and [*Ruminococcus*] *gauvreauii group* were also elevated at 28 dpi. Clostridia, Clostridiales, Lachnospiraceae, and *Lachnospiraceae NK4A136 group* were more abundant in the infected group at day 7, while *Luteimonas*, *Enterorhabdus*, and *Granulicella* were enriched in infected rats at 35 dpi (Fig. 4). These findings indicated that while *Bacteroides* plays a crucial part in the entry of *A. cantonensis* into the host’s brain, other species assume important roles after the parasite enters the lungs.

### Infection induced host-specific metagenome changes

High-throughput sequencing was performed on fecal samples from mice and rats. Host sequences were first removed, and the remaining non-redundant genes were annotated against the KEGG database. Among the annotated pathways of gut microbiota from mice and rats, metabolism contained the highest number of genes (Additional file 17).

**Fig. 5:**
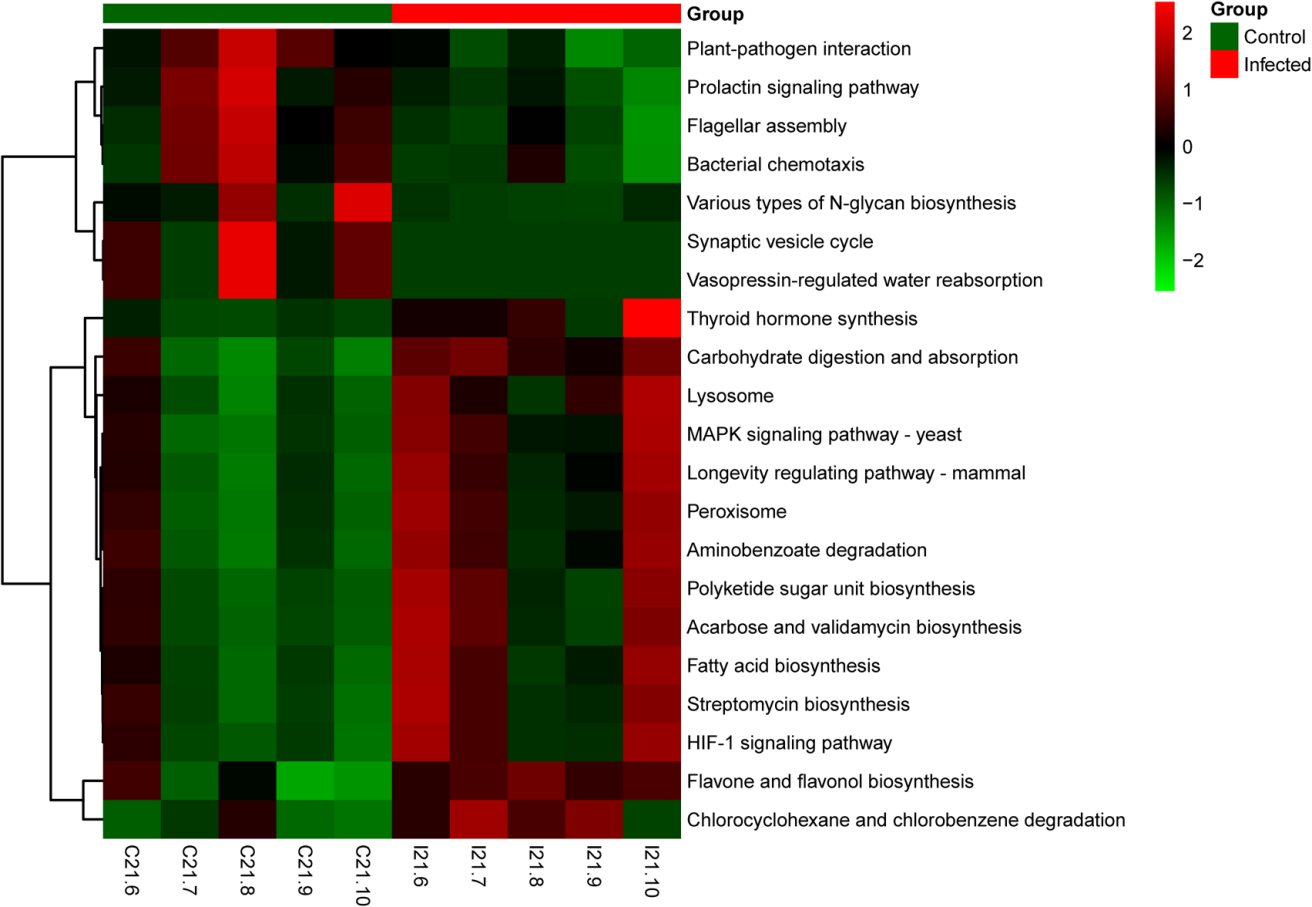
The significantly differential KEGG pathways of gut microbiome between control mice and *Angiostrongylus cantonensis*-infected mice. Samples are shown in columns, and KEGG pathways are shown in rows. *KEGG* Kyoto Encyclopedia of Genes and Genomes

To further analyze the differences, a hierarchical clustering heatmap was constructed based on the results of Wilcoxon rank sum test. The heatmap demonstrated that genes in the intestinal flora of infected mice were significantly enriched in several pathways, such as carbohydrate digestion and absorption, polyketide sugar unit biosynthesis, fatty acid biosynthesis, hypoxia-inducible factor-1 signaling pathway, and flavone and flavonol biosynthesis (Fig. 5).

**Fig. 6:**
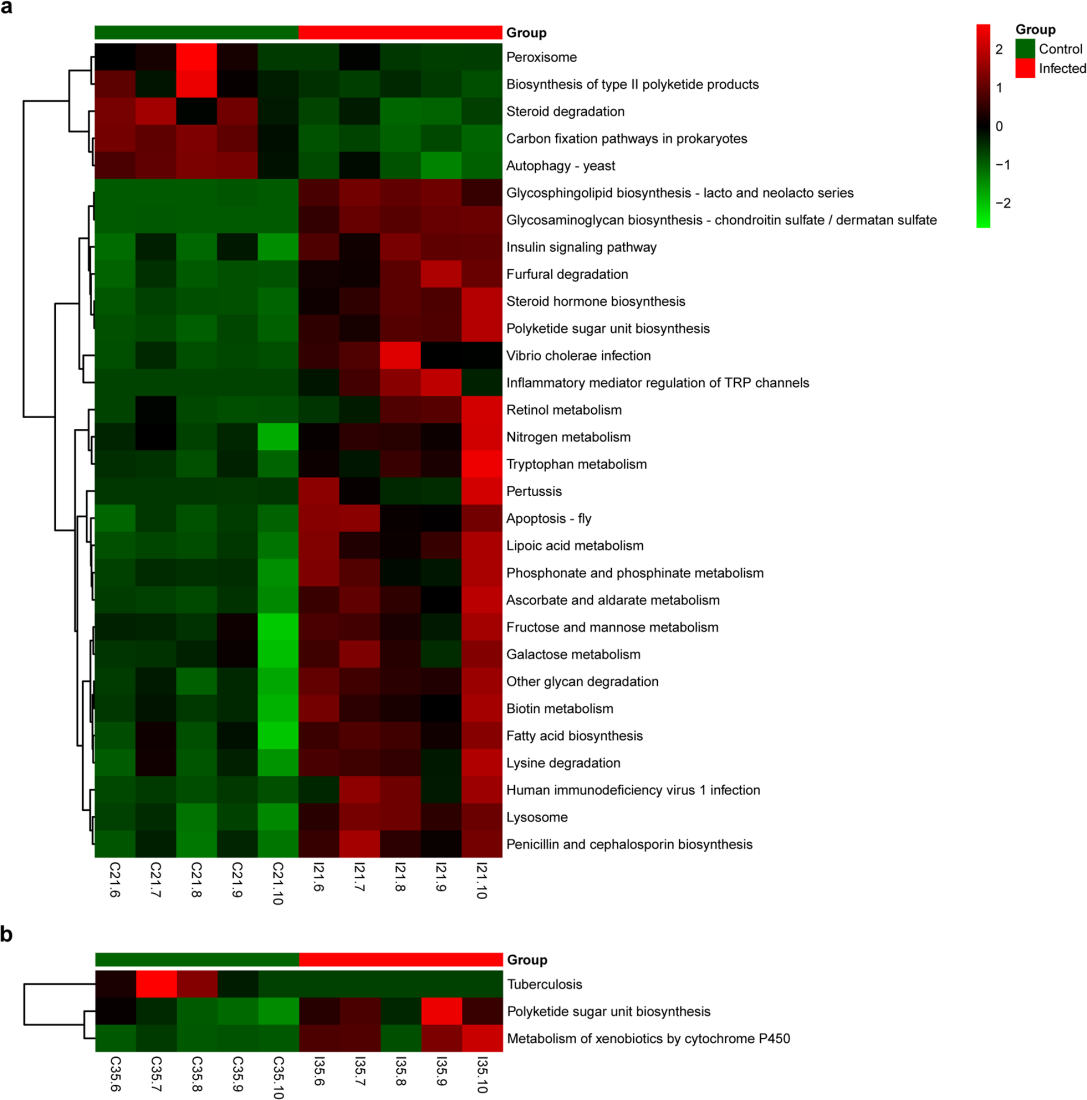
The significantly differential KEGG pathways of gut microbiome between control rats and *Angiostrongylus cantonensis*-infected rats. **a** Day 21. **b** Day 35. Samples are shown in columns, and KEGG pathways are shown in rows. *KEGG* Kyoto Encyclopedia of Genes and Genomes

In infected rats at 21 dpi, several metabolic pathways were significant up-regulated, including glycosphingolipid and glycosaminoglycan biosynthesis, ascorbate and aldarate metabolism, fructose and mannose metabolism, galactose metabolism, fatty acid and steroid hormone biosynthesis, tryptophan metabolism, lysine degradation, retinol and biotin metabolism, lipoic acid metabolism, nitrogen metabolism, and polyketide sugar unit biosynthesis (Fig. 6a). At 35 dpi, as the parasite matured in the lungs, the functional differences diminished, with only a few pathways like polyketide sugar unit biosynthesis and metabolism of xenobiotics by cytochrome P450 remaining significant (Fig. 6b).

### Systemic metabolic perturbations induced by infection

We next investigated the systemic metabolic consequences after infection using untargeted metabolomics on serum, urine, feces, and brain. In mice, differential metabolites were primarily glycerophospholipids, sphingomyelins, lysophospholipids, fatty acids, carnitines, amides, steroid hormones, cholic acids, vitamins, organic acids, amino acids, nucleotides, and purines. Cluster analysis using heatmaps (Additional files 30–33) was performed to visualize differences between infected and control groups. The heatmap indicated that, compared to control mice, differential metabolites in serum, urine and feces of infected mice were variably up-regulated or down-regulated, while changes in brain metabolites were relatively less pronounced.

In rats, differential metabolites were primarily categorized as glycerophospholipids, sphingomyelins, lysophospholipids, fatty acids, carnitines, steroid hormones, cholic acids, vitamins, organic acids, amino acids, and purines. Compared to infected mice, infected rats showed obvious increases in long-chain fatty acids and cholic acids, alongside decreases in SCFAs, amides, and amino acids. Additional files 34–37 present changes in the levels of differential metabolites in serum, urine, feces, and brain of infected rats compared to control rats, it is worth noting that the changes in brain were relatively insignificant compared to the other samples.

### Pathway analysis

**Fig. 7:**
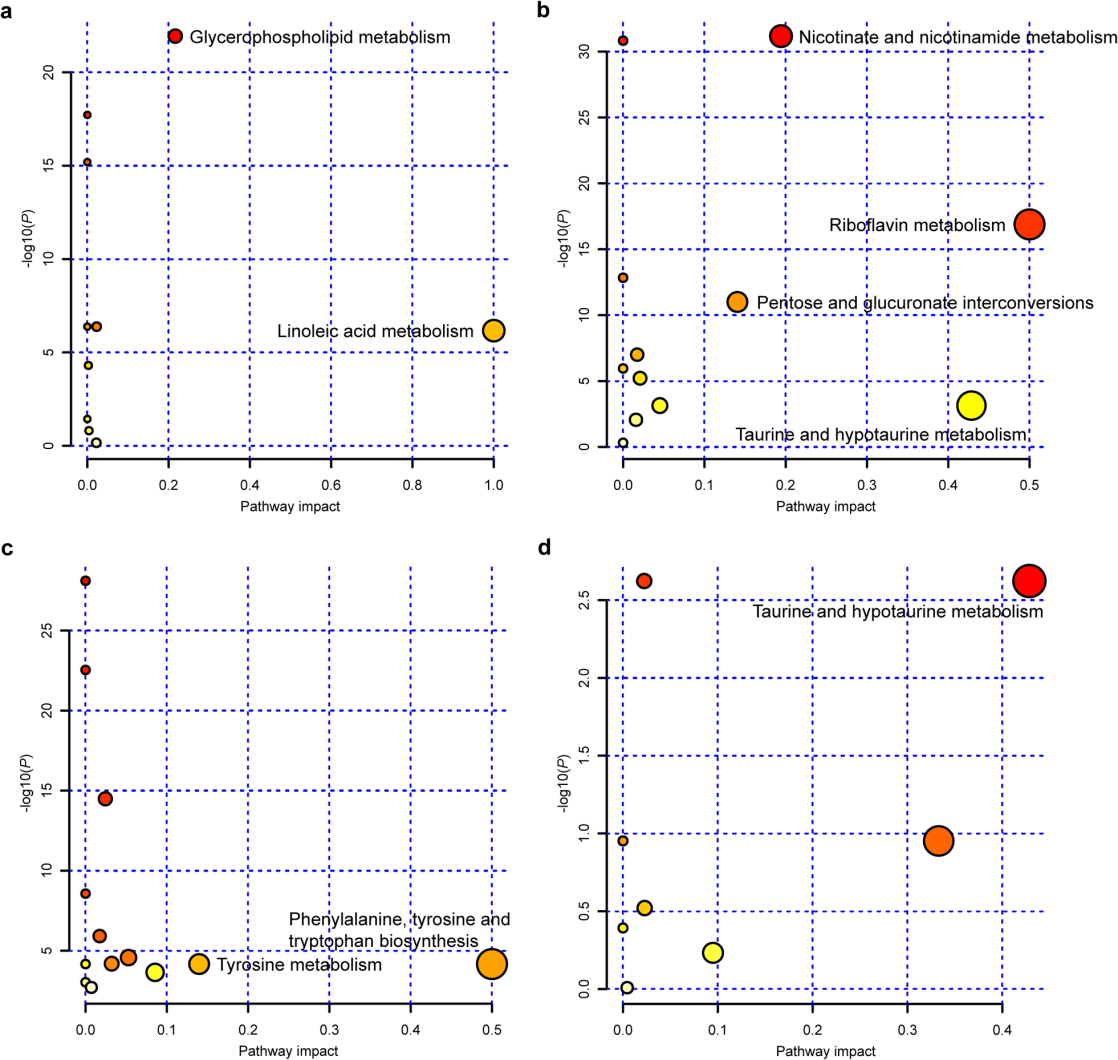
Metabolic pathway analysis of significantly differential metabolites in serum (**a**), urine (**b**), feces (**c**) and brain (**d**) between control mice and *Angiostrongylus cantonensis*-infected mice. The x-axis represents increasing metabolic pathway impact from pathway topology analysis, whereas the y-axis represents unadjusted *P*-value by pathway enrichment analysis. Greater pathway enrichment and higher pathway impact values are exhibited in larger sizes and darker colors, respectively

Pathway analysis using MetaboAnalyst revealed significant abnormalities in linoleic acid metabolism and glycerophospholipid metabolism in the serum of infected mice (Fig. 7a). Furthermore, four distinct metabolic pathways in urine were significantly affected, including riboflavin metabolism, taurine and hypotaurine metabolism, nicotinate and nicotinamide metabolism, and pentose and glucuronate interconversions (Fig. 7b). In addition, phenylalanine, tyrosine and tryptophan biosynthesis, as well as tyrosine metabolism, were markedly associated with infection in feces (Fig. 7c). Moreover, in the brain of mice, substantial perturbation in taurine and hypotaurine metabolism was observed (Fig. 7d).

**Fig. 8:**
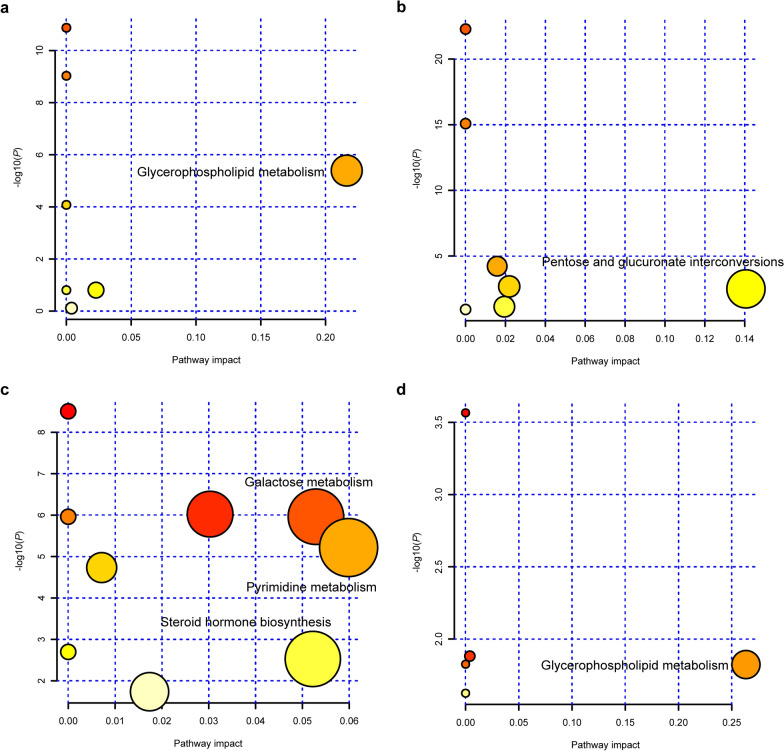
Metabolic pathway analysis of significantly differential metabolites in serum (**a**), urine (**b**), feces (**c**) and brain (**d**) between control rats and *Angiostrongylus cantonensis*-infected rats. The x-axis represents increasing metabolic pathway impact from pathway topology analysis, whereas the y-axis represents unadjusted *P*-value by pathway enrichment analysis. Greater pathway enrichment and higher pathway impact values are exhibited in larger sizes and darker colors, respectively

Compared to infected mice, infected rats exhibited conspicuous disturbances in glycerophospholipid metabolism in both the serum and brain (Fig. 8a, d), and pentose and glucuronate interconversions was dramatically affected in urine (Fig. 8b). Furthermore, three metabolic pathways in feces were strongly correlated with infection, specifically pyrimidine metabolism, galactose metabolism, and steroid hormone biosynthesis (Fig. 8c).

### Identification of potential early diagnostic biomarkers

**Fig. 9:**
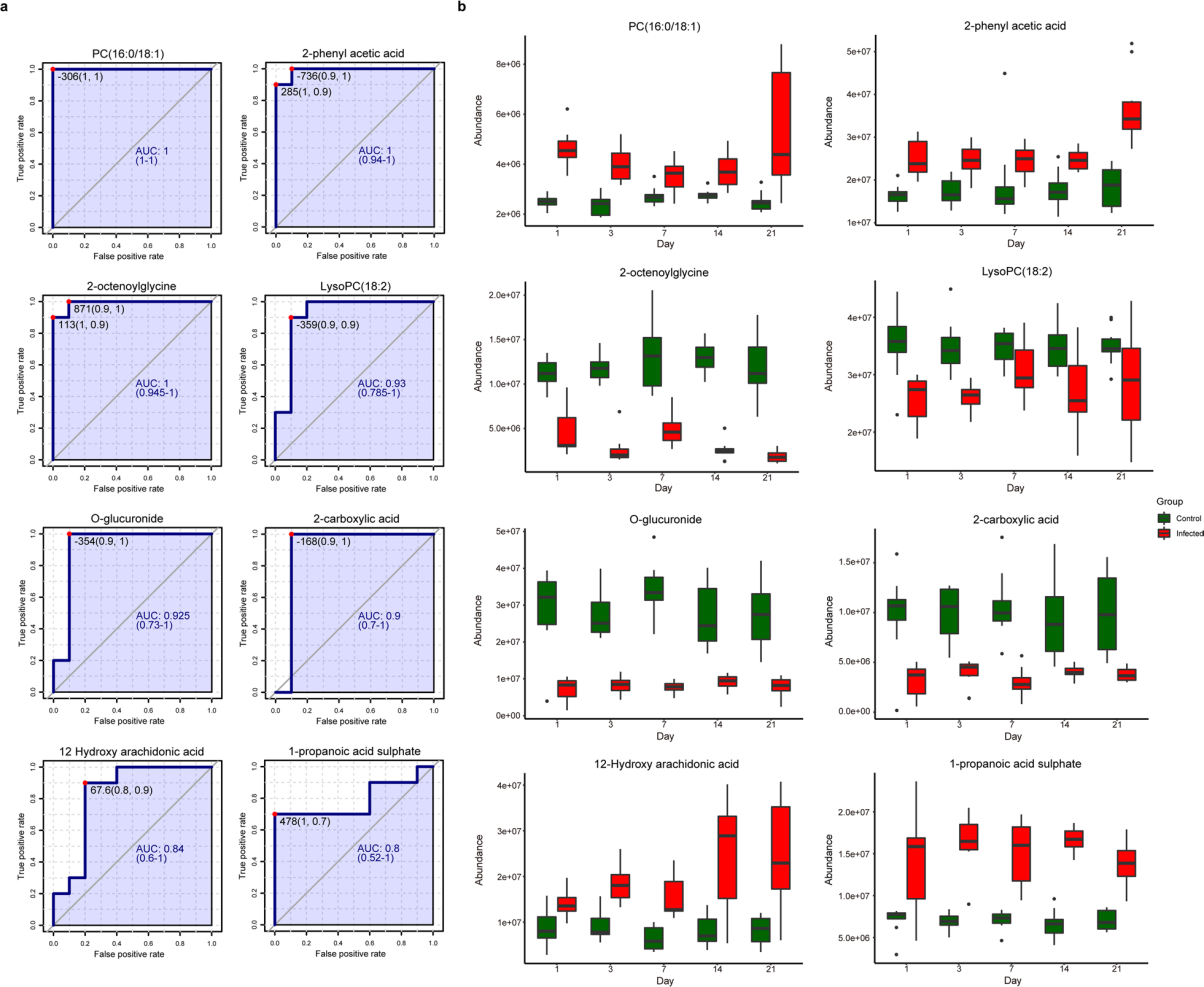
Overview of selected representative metabolites from serum and urine associated with angiostrongyliasis. **a** The ROC curves for selected biomarkers. The x-axis represents the specificity, while the y-axis represents the sensitivity. **b** Box plots showing the abundance of selected biomarkers. Each column represents a group

The identification of potential biomarkers for early diagnosis of *A. cantonensis* infection was conducted by screening differential compounds with VIP values > 10.0 in serum and urine samples from infected mice compared to control mice at day 1. The diagnostic performance of each compound was evaluated using the area under the curve (AUC). Among eight differential metabolites, phosphatidylcholine (16:0/18:1) (VIP = 10.017), 2-phenyl acetic acid (VIP = 14.806), and 2-octenoylglycine (VIP = 13.640) exhibited the highest AUC value (AUC = 1). The AUC values of lysophosphatidylcholine (18:2) (VIP = 10.640, AUC = 0.93), O-glucuronide (VIP = 23.963, AUC = 0.925), 2-carboxylic acid (VIP = 10.542, AUC = 0.9), 12 hydroxy arachidonic acid (VIP = 12.407, AUC = 0.84), and 1-propanoic acid sulphate (VIP = 12.214, AUC = 0.8) decreased, but remained greater than 0.8. Notably, 1-propanoic acid sulphate exhibited lower sensitivity, and 12 hydroxy arachidonic acid showed lower specificity (Fig. 9a). In addition, infection led to an increase in phosphatidylcholine (16:0/18:1) and 12 hydroxy arachidonic acid levels in serum, as well as 2-phenyl acetic acid and 1-propanoic acid sulphate levels in urine. Conversely, lysophosphatidylcholine (18:2) levels in serum, and 2-octenoylglycine, O-glucuronide, and 2-carboxylic acid levels in urine, decreased after infection (Fig. 9b).

### Relationships between host metabolome and gut microbiome

**Fig. 10:**
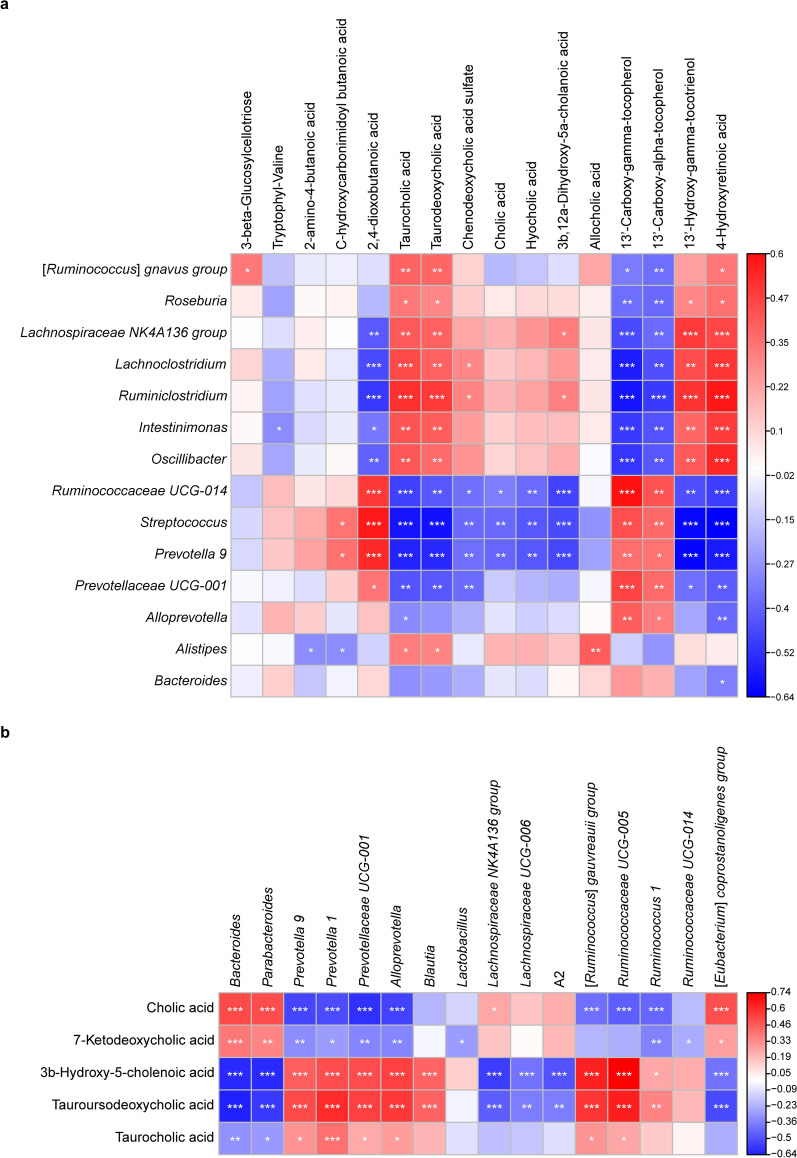
Correlation analysis of differential metabolites in feces and differential genera in gut between control mice and *Angiostrongylus cantonensis*-infected mice (**a**) and control rats and *A. cantonensis*-infected rats (**b**). The correlations between them are exhibited by colors; red indicates a positive correlation, blue indicates a negative correlation, and a darker color illustrates a stronger correlation (*P < 0.05, **P < 0.01, ***P < 0.001)

Spearman correlation analysis between significantly altered metabolites and differential bacterial genera in mouse feces revealed significant correlations. For instance, the level of 2, 4-dioxobutanoic acid was markedly increased in feces of infected mice, positively correlated with enriched *Ruminococcaceae UCG-014*, *Streptococcus*, *Prevotella 9*, and *Prevotellaceae UCG-001*. Conversely, it showed negative associations with *Lachnospiraceae NK4A136 group*, *Lachnoclostridium*, *Ruminiclostridium*, *Intestinimonas*, and *Oscillibacter*, all of which were reduced in feces of infected mice. After infection, the dramatically down-regulated taurocholic acid (TCA), taurodeoxycholic acid, cholic acid (CA), chenodeoxycholic acid sulfate, 13’-hydroxy-gamma-tocotrienol, and 4-hydroxyretinoic acid were positively correlated with [*Ruminococcus*] *gnavus group*, *Roseburia*, *Lachnospiraceae NK4A136 group*, *Lachnoclostridium*, *Ruminiclostridium*, *Intestinimonas*, and *Oscillibacter*, all enriched in control mice; while negatively correlated with *Ruminococcaceae UCG-014*, *Streptococcus*, *Prevotella 9*, and *Prevotellaceae UCG-001*. An intriguing observation was the opposite associations of the up-regulated 13’-carboxy-gamma-tocopherol and 13’-carboxy-alpha-tocopherol with the aforementioned genera. Moreover, *Bacteroides*, more abundant in infected mice, was only negatively related to the down-regulated 4-hydroxyretinoic acid (Fig. 10a).

For rats, Spearman correlation analysis revealed that increased CA and 7-ketodeoxycholic acid (7-KHCA) showed markedly positive relations with bacteria enriched in the infected group (including *Bacteroides*, *Parabacteroides*, and [*Eubacterium*] *coprostanoligenes group*), but were negatively correlated with bacterial flora enriched in the control group, such as *Prevotella 9*, *Prevotellaceae UCG-001*, and *Ruminococcaceae UCG-005*. Notably, [*Ruminococcus*] *gauvreauii group*, which exhibited a positive response to infection, was negatively associated with CA and 7-KHCA. Interestingly, 3β-hydroxy-5-cholenoic acid, tauroursodeoxycholic acid (TUDCA), and TCA, which decreased after infection, exhibited opposite associations with the aforementioned genera (Fig. 10b). These findings suggest a close relationship between alterations in BAs in feces and serum and specific changes in gut microbiota in infected rats.

### BA metabolism is severely disrupted in *A. cantonensis*-infected mice

**Fig. 11:**
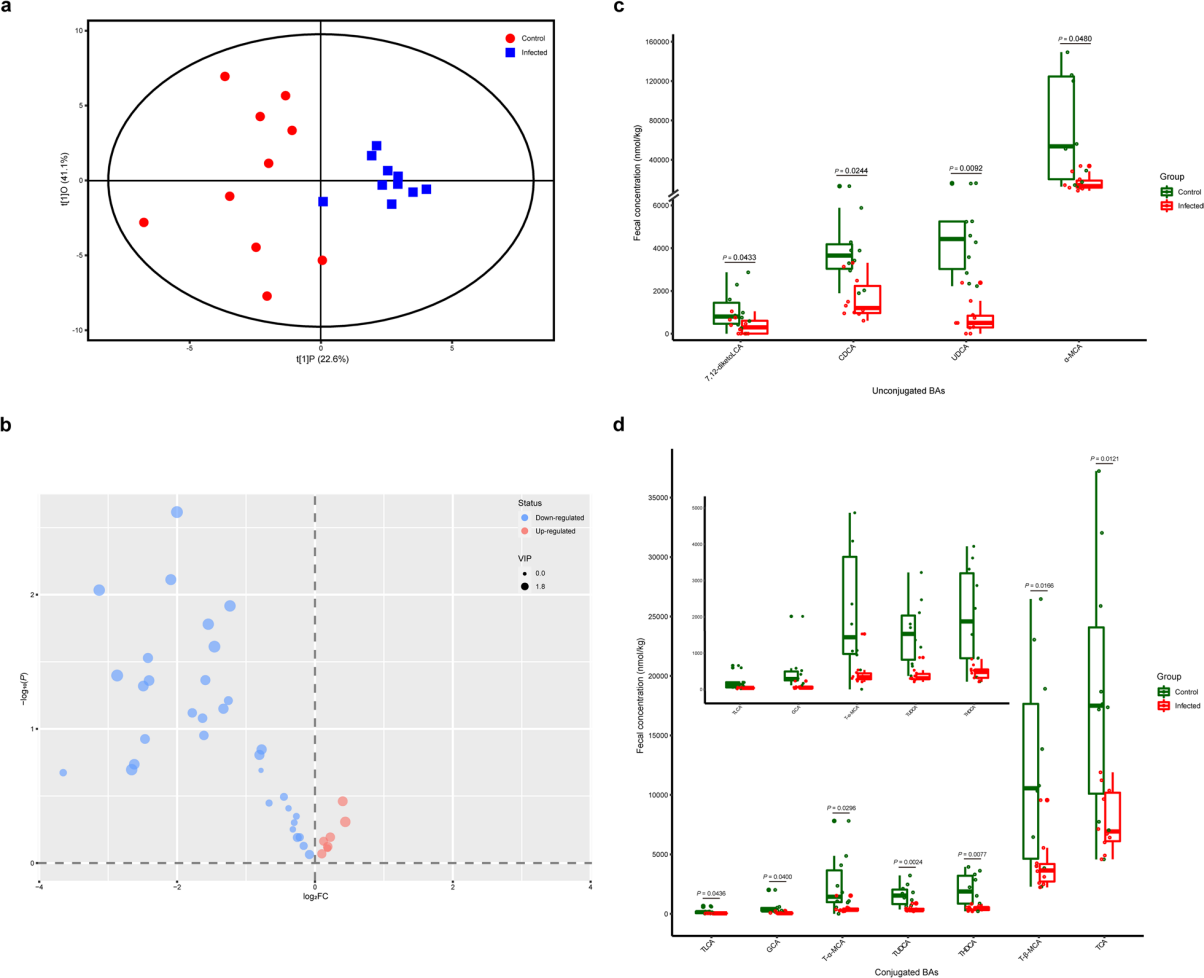
BAs were measured in fecal samples from control mice and *Angiostrongylus cantonensis*-infected mice at 21 dpi, using targeted quantitative metabolomics. **a** OPLS-DA plot of BAs metabolic profiles. Each point represents one sample in score plot. **b** Volcano plot of BAs. Each point shows one kind of BA, and the size of point is positively associates with the VIP value of OPLS-DA model. The red point indicates an up-regulated metabolite, while the blue point indicates a down-regulated metabolite. **c** Concentration of significantly altered unconjugated BAs in feces of the *A. cantonensis*-infected mice. Each column represents one group, the top and the bottom whiskers indicate the maximum and the minimum values, respectively, and the hyphen represents the median value. **d** Concentration of markedly altered conjugated BAs in feces of the *A. cantonensis*-infected mice. Each column represents one group, the top and the bottom whiskers indicate the maximum and the minimum values, respectively, and the hyphen represents the median value. *BAs* bile acids; *OPLS-DA* orthogonal partial least squares-discriminatory analysis; *VIP* variable importance in the projection; *7,12-diketoLCA* 7,12-diketolithocholic acid; *CDCA* chenodeoxycholic acid; *UDCA* ursodeoxycholic acid; *α-MCA* α-muricholic acid; *TLCA* taurolithocholic acid; *GCA* glycocholic acid; *T-α-MCA* tauro α-muricholic acid; *TUDCA* tauroursodeoxycholic acid; *THDCA* taurohyodeoxycholic acid; *T-β-MCA* tauro β-muricholic acid; *TCA* taurocholic acid

The composition of BAs was analyzed in 20 fecal samples from control mice and infected mice at day 21 (10 samples per group) using a targeted metabolomics approach. As expected, the OPLS-DA plot clearly discriminated between *A. cantonensis*-infected mice and control mice (Fig. 11a). Subsequently, a volcano plot revealed that 33 BAs were reduced; among these, 11 BAs decreased significantly (*P* < 0.05), with TUDCA being the most significantly affected, while 7 BAs showed insignificant increases (*P* > 0.05) (Fig. 11b). Moreover, comparison of BA profile components revealed a marked reduction of four unconjugated BAs: 7,12-diketolithocholic acid (7,12-diketoLCA), chenodeoxycholic acid (CDCA), ursodeoxycholic acid (UDCA), and α-muricholic acid (α-MCA) in infected mice (*P* < 0.05) (Fig. 11c). The infection also led to a significant decrease in the levels of seven conjugated BAs: taurolithocholic acid (TLCA), glycocholic acid (GCA), tauro α-muricholic acid (T-α-MCA), TUDCA, taurohyodeoxycholic acid (THDCA), tauro β-muricholic acid (T-β-MCA), and TCA (*P* < 0.05) (Fig. 11d). The alteration in BA composition, along with decreased concentrations of total BAs, further confirmed the perturbation of BA metabolism in *A. cantonensis*-infected mice.

## Discussion

This integrative multi-omics study provides a comprehensive, longitudinal atlas of the host-gut microbiota-metabolite axis during *A. cantonensis* infection, revealing profound and host-specific reprogramming. Our findings underscore that the pathological outcome in angiostrongyliasis is not solely a function of direct parasitic damage but is likely modulated by complex systemic interactions involving the gut microbiome and host metabolism. However, it is crucial to consider the inherent biological differences between the hosts used in this study. Rats are the natural suitable host for *A. cantonensis*, whereas mice are a non-suitable host. This fundamental difference in host suitability profoundly affects larval development, migration, immune responses, and ultimately the disease pathophysiology. Furthermore, the infection doses administered (30 L3 for mice vs. 100 L3 for rats) were not equal, which may lead to differences in infection severity and host response intensity. Therefore, some of the observed “species-specific” patterns in gut microbiota and metabolome may reflect a combination of host permissiveness, dose-dependent effects, and distinct immune-pathological dynamics, rather than purely interspecies biological differences. Our comparative analysis should thus be interpreted within this context, highlighting general patterns of host-parasite-microbiome interaction while acknowledging these confounding factors.

### Different hosts infected with *A. cantonensis* led to various changes in the structure and function of gut microbiome

The alpha diversity of intestinal flora in both non-suitable and suitable hosts decreased after infection; however, the difference was not statistically significant. Generally, during disease states, especially intestinal diseases, the diversity of gut flora reduces, which indirectly reflects an ecological imbalance of intestinal microorganisms. For instance, reduced alpha diversity of gut microbiota has been detected in both schistosome and hookworm infection models [34–36]. However, following *A. cantonensis* infection, the structure of gut bacteria in both non-suitable and suitable hosts was markedly altered compared to age-matched controls, though this change gradually diminished during the late stage of infection. This could be attributed to the parasite leaving the enteric cavity and entering the brain or lungs through the bloodstream, thereby reducing its direct interaction with the gut environment. However, it should be noted that infection-induced anorexia, weight loss, and general malnutrition are common in parasitized hosts [37]. These factors can independently alter gut motility, secretion, and the nutritional landscape of the intestine, thereby shaping the microbiota community. While we observed weight loss in infected hosts, detailed food intake records were not kept. Therefore, we cannot rule out the possibility that some of the observed microbial perturbations are secondary to changes in host nutritional status and feeding behavior.

The composition of intestinal flora differed between infected and control hosts at multiple taxonomic levels. At the phylum level, there was a significant decrease in Firmicutes and a noticeable increase in Bacteroidetes in mice and rats during the early stage of infection. Bacteria from Firmicutes help hosts to absorb and store energy from food, while microbiota from Bacteroidetes have the opposite effect. As a result, the intestinal flora of obese patients exhibited an increased ratio of Firmicutes to Bacteroidetes [38]. Although we did not observe a marked change in this ratio in *A. cantonensis*-infected hosts, the significant reduction of Firmicutes was likely associated with the host’s weight loss during the late stage of infection. Down-regulation of Firmicutes has also been observed in ASD, while up-regulation of Bacteroidetes and Proteobacteria has been observed in PD and depression [39, 40]. This reduction of Firmicutes weakens the protective effect of intestinal flora and increases the host’s susceptibility to inflammation, considering that most SCFA-producing bacteria belong to Firmicutes [41]. In addition, the abundance of Proteobacteria was also dramatically increased in *A. cantonensis*-infected hosts. Bacteria of Proteobacteria are mainly Gram-negative or opportunistic pathogens, which can produce lipopolysaccharide and induce systemic chronic low-grade inflammation. In mice infected with *A. cantonensis* at 1 and 7 dpi, the abundance of Burkholderiaceae and Enterobacteriaceae of Proteobacteria increased dramatically, along with a significant rise in Actinobacteria. Moreover, *Lactobacillus*, which is often considered a probiotic genus due to its association with health benefits in other contexts, showed a marked increase at 7 dpi. *Lactobacillus* is known to maintain the balance of intestinal microecology, bind to pattern recognition receptors like Toll-like receptors, trigger the host's innate and adaptive immune responses, and regulate the host's immune reactions [42, 43]. Up-regulation of *Lactobacillus* has also been observed in patients with PD and ASD [39, 40], and studies have demonstrated its ability to increase gastric vagal nerve activity, reduce level of gamma-aminobutyric acid (GABA) in the hippocampus, and improve cognitive impairment in patients with AD; metabolites of *Lactobacillus* can also decrease inflammatory levels in the brain [44, 45]. However, *Lactobacillus* in the gut of mice was not continuously elevated, resulting in a severe inflammatory response in the mouse brain during the late stage of infection, characterized by significant increases in inflammatory factors, such as IL-5, IL-13, IL-10, IL-6, IL-1β, TNF-α, and IFN-γ [19], accompanied by cognitive functional disorder. On the other hand, rats showed down-regulation of Actinobacteria, Lactobacillaceae, and *Lactobacillus* after infection.

At the genus level, the differential microbiome of infected mice and rats were mainly classified into Firmicutes and Bacteroidetes. Specifically, we focused our investigation on *Roseburia*, *Ruminococcus*, *Blautia* (phylum Firmicutes), and *Bacteroides* (phylum Bacteroidetes), which played significant roles in SCFA metabolism, while *Bacteroides* also participated in bile salt metabolism [46, 47]. In infected mice, *Roseburia* was markedly decreased, while in infected rats, both *Ruminococcus* and *Blautia* were significantly reduced. However, *Bacteroides* was obviously up-regulated in both infected mice and rats, and maintained continuous up-regulation in the latter. Due to the higher relative abundance of *Bacteroides* compared to *Roseburia*, *Blautia*, and *Ruminococcus*, infection with *A. cantonensis* did not notably reduce SCFA levels in the hosts.

Prevotellaceae are intestinal commensal bacteria that participate in the production of mucins in the intestinal mucosa and the synthesis of SCFAs; therefore, a decrease in their abundance can be linked to decreased mucin synthesis and increased intestinal permeability [48, 49]. Previous studies have reported reduced abundance of Prevotellaceae or *Prevotella* in the gut microbiota of individuals with PD and multiple sclerosis (MS) [50, 51]. However, we observed that while the abundance of Prevotellaceae was dramatically decreased in infected rats, it showed a notable increase in infected mice. The reasons for this divergence are likely multifactorial, rooted in distinct host-parasite relationships. In the non-suitable host, the intense and often dysregulated Th2-polarized inflammation, possibly coupled with greater intestinal disturbance from larval migration, may create a microenvironment that favors the expansion of Prevotellaceae, which has been associated with inflammatory conditions in some models [52]. Conversely, in the suitable host, the parasite may establish a more modulated interaction, potentially secreting immunoregulatory molecules that differently shape the intestinal immune milieu and microbial ecology, leading to Prevotellaceae suppression. This highlights how host immune status, dictated by permissiveness, can critically determine the trajectory of specific microbial shifts. Further studies have highlighted the dual role of *Prevotella*, which can produce SCFAs while also activating immune cells to release pro-inflammatory factors, and elevated levels of *Prevotella* may be associated with reduced functional activity of the hippocampus [53, 54].

In addition, our findings indicated that certain genera of Ruminococcaceae, namely *Ruminococcus 1*, *Ruminococcaceae UCG-005*, and *Ruminococcaceae UCG-014*, had synergistic effects with Prevotellaceae. Consequently, they exhibited a consistent variation trend in infected hosts alongside Prevotellaceae. Notably, both Ruminococcaceae and *Ruminococcus* were obviously reduced in *A. cantonensis*-infected rats, mirroring observations made in AD, ASD, and PD [39, 55]. Other studies have demonstrated that *Ruminococcus* has a positive correlation with glutamate and glutamine, which are closely related to epilepsy onset [56], and a negative correlation with the reduction of 5-hydroxytryptamine (5-HT) in PD [57, 58]. As a result, we hypothesize that the marked reduction in the abundance of *Ruminococcus* in rats from 21 days post *A. cantonensis* infection may alleviate cognitive decline in the host. Although the abundance of *Ruminiclostridium* and *Oscillibacter* (family Ruminococcaceae) were significantly decreased in infected mice, *Ruminococcaceae UCG-014*, with a higher relative abundance, was up-regulated following infection.

The above results indicate that the colonized intestinal flora of different hosts altered markedly after *A. cantonensis* infection. The intensity of the inflammatory response in the brains of different hosts infected with *A. cantonensis* may be the result of the interaction of multiple bacteria, depending on which bacteria are dominant. In general, after *A. cantonensis* infection, mouse intestinal bacteria respond positively to the pathogen invasion, whereas rat gut microbes respond relatively negatively, this disparity may contribute to the parasite’s inability to develop and mature in mice. Functional changes in the host’s gut microbiota during the late stage of infection may alleviate the energy deficiency caused by *A. cantonensis*.

### *A. cantonensis* infection affected the lipid metabolism of the hosts

Despite *A. cantonensis* parasitizing the host’s CNS and leading to severe CNS inflammation, metabolic alterations in the host’s brain are less pronounced than those in serum, urine, and feces. This may reflect the pathological niche of *A. cantonensis*; as a non-intracellular parasite in the meninges, it may exert its effects more through physical damage and eliciting a vigorous host immune infiltrate (eosinophilic meningitis) rather than through direct, widespread manipulation of brain parenchymal cell metabolism. The systemic metabolic disturbances observed are likely a combination of the host's acute phase response, liver involvement, and gut dysbiosis. However, glycerophospholipid metabolism was the most profoundly affected by *A. cantonensis* infection in both suitable and non-suitable hosts. Glycerophospholipids, the predominant phospholipids in the body, serve as vital components of biological membranes and participate in protein recognition and signal transduction by cell membranes. Our results indicate that the majority of glycerophospholipids were up-regulated in infected hosts, with the most substantial increase observed from 14 dpi, followed by a declining trend at 35 dpi. Glycerophospholipids were mainly detected in the host’s serum and brain, and their elevation may be linked to cellular repair and proliferation following inflammation, cell damage, or apoptosis. Moreover, levels of certain lysophosphatidylcholine were also obviously increased following infection, a pattern also observed in patients with MS [59]. Lysophosphatidylcholine, generated through the catalytic activity of phospholipase A2 and cholesterol acyltransferase from phosphatidylcholine, plays a crucial role in stimulating or regulating immune cells and exhibits both pro-inflammatory and anti-inflammatory properties, an elevation in its level indicates cell membrane damage [60].

At the same time, disorders of sphingomyelin metabolism were also observed in infected hosts, mainly characterized by increased sphingomyelin levels, while up-regulation of ceramides has also been observed in patients with MS and may be associated with axonal injury [61]. Sphingomyelin not only protects cells from environmental harm, but also participates in cell signal transduction as a second messenger, mediating cell proliferation and apoptosis, etc. [60, 62, 63]. Ceramides are synthesized through the activation of sphingomyelinase, and activation of the sphingomyelinase-ceramide pathway is known to promote pro-inflammatory and pro-oxidative effects and can lead to cell autophagy and apoptosis [64–66]. The up-regulation of ceramides observed in our study suggests potential involvement of this pathway, which might contribute to the inflammatory milieu in the host, possibly facilitating parasite clearance.

Steroid hormones are a category of tetracyclic aliphatic hydrocarbon compounds that can penetrate the BBB and play an important role in maintaining the body's metabolic function, regulating inflammatory responses and immune reactions. Following infection with *A. cantonensis*, levels of glucocorticoids and sex hormones in the hosts decreased initially, but began to rise from 28 dpi. The down-regulation of steroid hormones aggravated the host’s metabolic disorders; however, no significant changes in cholesterol were observed. Interestingly, alterations in sex hormones can impact a variety of neurotransmitters, such as GABA, 5-HT, dopamine and glutamate, etc. In addition, sex hormones influence the structure of the nervous system through processes such as myelination, nerve pruning, dendritic spine remodeling, and cell apoptosis, and take part in the regulation of the body's mood, behavior, and cognitive ability [67–70]. Other studies have demonstrated that allopregnanolone positively and allosterically regulates GABA receptors, reduces BBB dysfunction and neuroinflammation, and exerts neuroprotective effects in conditions such as AD, MS, and PD [71–75].

In conclusion, lipid metabolism disorders are the most noticeable metabolism changes in *A. cantonensis*-infected hosts, and several studies have indicated that lipid metabolism disorders are associated with a variety of mental disorders, including depression, AD, and anxiety [76–78]. Correcting the metabolism disorder caused by *A. cantonensis* infection through supplementation or inhibition of the synthesis of crucial metabolites is expected to improve the clinical symptoms of patients and delay disease progression. However, it is important to note that perturbations in lipid metabolism are not specific to *A. cantonensis* infection but are common hallmarks of systemic inflammation, cellular stress, and tissue injury observed in various pathological states [79, 80]. Therefore, while these changes are significant features of the host response to *A. cantonensis*, they likely reflect a generalized metabolic adaptation to infection and inflammation.

Moreover, an important consideration in interpreting gut microbiota and metabolite changes during a systemic infection is distinguishing direct effects of the parasite from indirect effects mediated by the host's inflammatory response. Because *A. cantonensis* infection triggers a strong Th2-type systemic immune response, the observed microbial shifts likely represent an integrated outcome of direct host-parasite interactions within the gut lumen (especially early stage) and subsequent systemic host-driven changes. Future studies using heat-killed parasites, parasite excretory/secretory products, or interventions that modulate specific immune pathways could help dissect these contributions. Many of the observed differences between *A. cantonensis*-infected mice and rats—including microbial and metabolic patterns—are ultimately downstream of the fundamental disparity in parasite fate: successful development to adulthood in the rat lung versus arrested development and death in the mouse CNS. Therefore, factors like parasite burden over time, tissue-specific damage, and the duration and nature of the immune response, all of which differ between hosts, are primary drivers that likely shape the secondary effects on the microbiome and metabolome.

### *A. cantonensis* infection influenced the BA metabolism of the hosts

It is worth noting that the content of BAs in the host changes pronouncedly after infection with *A. cantonensis*, and gut flora plays a role in the process of producing secondary BAs after dehydrogenation and dehydroxylation of primary BAs [81]. Our study revealed that both primary and secondary BAs in the feces of infected mice were decreased. This may be due to a reduced synthesis of BAs in the liver, resulting in lower levels of conjugated BAs and a consequent down-regulation of primary BAs. The liver, an important metabolic organ and primary site of lipid synthesis, the disorder in metabolism observed in *A. cantonensis*-infected hosts indirectly indicates that liver function was affected to some extent. Therefore, decreased activity of certain enzymes involved in BA synthesis may be one reason for the reduction in secondary BAs. Another possible reason is that infection caused a decrease in taurine levels in mice, which leads to a decline in the synthesis of conjugated BAs.

An increase in unconjugated BAs and a decrease in conjugated BAs were observed in *A. cantonensis*-infected rats. Since there was no pronounced change in taurine in infected rats, these alterations in host BA metabolism may be linked to the up-regulated *Bacteroides* caused by the infection. Another possibility is that the host’s liver function is impaired, preventing the recycling of BAs enter the liver through the portal vein, thereby causing elevated levels of conjugated BAs in the serum. At the same time, BAs can regulate changes in gut bacteria, maintain intestinal homeostasis by promoting the growth of BA-metabolizing bacteria, controlling the proliferation of BA-sensitive bacteria [82], and reducing the occurrence of bacterial translocation and endotoxemia [83].

Although studies have shown that BAs are altered in various CNS diseases [84–86], the mechanism of BAs in diseases pathogenesis remains unclear. Targeting BA signaling or treatment with the neuroprotective UDCA has been proved to alleviate disease symptoms [87–89]. TUDCA suppresses neuronal apoptosis, the activation of astrocytes and microglia, regulates the synthesis of annexin A1 in microglia, represses up-regulation of pro-inflammatory factors and down-regulation of anti-inflammatory factors in the brain, alleviates neuroinflammation in PD, and improves cognitive deficiency in AD [90–93]. These findings indicate that applying BAs in the treatment of *A. cantonensis* infection might reduce the inflammatory response in the host’s brain and inhibit microglia activation. Future studies should investigate whether these infection-induced changes in BA profiles lead to altered signaling through host BA receptors such as the farnesoid X receptor (FXR) and Takeda G protein-coupled receptor 5 (TGR5), which are known regulators of metabolism, inflammation, and neuroprotection [94]. Furthermore, we noticed that although the synthesis pathway of primary BAs was affected in infected rats, the changes were not as significant as in infected mice.

### Translational potential of metabolic biomarkers

The identified early metabolic biomarkers in the mouse model offer a promising, non-invasive approach for diagnosis. Their detection in urine or serum could overcome the limitations of current methods. However, their diagnostic efficacy, specificity, and consistency across different host species and, crucially, in patients, require extensive validation in future clinical studies. Factors such as dietary composition, baseline gut microbiota variation, and co-morbidities could significantly influence the levels of these metabolites.

### Limitations

This study has several limitations that should be considered. First, the integration of metabolomics and microbiome data in this study is largely correlative. While we identified significant associations between specific bacterial genera and metabolite changes, these observations do not establish causality or direct functional mechanisms. Future studies employing experimental validation (e.g., in vitro cultures, germ-free mice models) are needed to elucidate the precise mechanistic roles of the identified microbiota in shaping the host metabolome during infection. Second, to move beyond correlation, direct experimental manipulation of the gut microbiome is needed. Future studies should employ techniques such as fecal microbiota transplantation from infected versus control donors into germ-free or antibiotic-treated recipients. These approaches would directly test whether the infection-altered microbiota is sufficient to modulate disease severity, CNS inflammation, or metabolic profiles. Third, the correlations we observed between specific gut bacteria, circulating metabolites, and the known neuroinflammatory context of angiostrongyliasis are intriguing. However, they do not establish a causal direction. It is equally possible that CNS inflammation secondarily affects the gut via the brain-gut axis, influencing the microbiota and metabolome. To establish causality, future studies employing fecal microbiota transplantation from infected to naive hosts, or selective modulation of key metabolites, are essential. Fourth, the sample size for each group and time point was determined based on common practice in rodent omics studies and feasibility. While this provided robust data for group-level comparisons, a formal power calculation was not performed. Larger sample sizes might reveal more subtle changes and improve generalizability. Fifth, the potential early-diagnostic biomarkers were identified and their performance (AUC values) evaluated using the same dataset. This approach carries a high risk of overfitting and over-optimistic performance estimates. External validation in an independent cohort of infected mice, and ultimately in patient samples, is absolutely required before any clinical relevance can be claimed. Sixth, this study utilized only female animals to avoid behavioral confounding factors in group housing. However, significant sex differences exist in immune responses, microbiome composition, and metabolism. Therefore, our findings may not be fully generalizable to male hosts, and future studies should include both sexes to comprehensively understand host-parasite interactions.

## Conclusions

This multi-omics analysis reveals that *A. cantonensis* infection causes a host-specific dysregulation of the gut microbiome and metabolome. The most salient finding is the severe disruption of host lipid and BA metabolism, which is more pronounced in the non-suitable host and correlates with worse pathological outcomes. This metabolic reprogramming, mediated in part by specific shifts in gut microbial communities, represents a central biological insight into the systemic pathogenesis of angiostrongyliasis. We hypothesize that these microbial and metabolic changes are not merely epiphenomena but may actively influence disease progression and outcome. Furthermore, our data generate the hypothesis that specific metabolic signatures in biofluids could serve as early diagnostic markers. Validating these hypotheses will require functional experiments manipulating the microbiome or key metabolites, and translational studies in patients.

## Supplementary Information

Additional file1

Additional file2

Additional file3

Additional file4

Additional file5

Additional file6

Additional file7

Additional file8

Additional file9

Additional file10

Additional file11

Additional file12

Additional file13

Additional file14

Additional file15

Additional file16

Additional file17

Additional file18

Additional file19

Additional file20

Additional file21

Additional file22

Additional file23

Additional file24

Additional file25

Additional file26

Additional file27

Additional file28

Additional file29

Additional file30

Additional file31

Additional file32

Additional file33

Additional file34

Additional file35

Additional file36

Additional file37

## Data Availability

The sequencing data of 16S rRNA gene have been deposited in the NCBI Sequence Read Archive (SRA) under the project number PRJNA1041488 and PRJNA1041579. All other data supporting the findings of this study are available within the article and its supplementary files.

## Abbreviations

5-HT: 5-Hydroxytryptamine
7,12-diketoLCA: 7,12-Diketolithocholic acid
7-KHCA: 7-Ketodeoxycholic acid
*A. cantonensis*: *Angiostrongylus cantonensis*
AD: Alzheimer's disease
ASD: Autism spectrum disorders
AUC: Area under the curve
BAs: Bile acids
BBB: Blood-brain barrier
CA: Cholic acid
CDCA: Chenodeoxycholic acid
CNS: Central nervous system
CSF: Cerebrospinal fluid
dpi: Days post-infection
ENS: Enteric nervous system
ESI: Electrospray ionization
GABA: Gamma-aminobutyric acid
GCA: Glycocholic acid
HMDB: Human metabolome database
KEGG: Kyoto encyclopedia of genes and genomes
L3: Third-stage larvae
L5: Fifth-stage larvae
LEfSe: Linear discriminant effect size
MS: Multiple sclerosis
*m/z*: Mass/charge
OPLS-DA: Orthogonal partial least squares-discriminatory analysis
ORFs: Open reading frames
OTUs: Operational taxonomic units
PCA: Principal component analysis
PCoA: Principal coordinate analysis
PD: Parkinson’s disease
PRM: Parallel reaction monitoring
QC: Quality control
Q/TOF–MS: Quadrupole-time-of-flight mass spectrometry
*R. norvegicus*: *Rattus norvegicus*
ROC: Receiver operating characteristic
SCFAs: Short-chain fatty acids
SD: Sprague Dawley
TCA: Taurocholic acid
THDCA: Taurohyodeoxycholic acid
TLCA: Taurolithocholic acid
TUDCA: Tauroursodeoxycholic acid
T-α-MCA: Tauro α-muricholic acid
T-β-MCA: Tauro β-muricholic acid
UDCA: Ursodeoxycholic acid
UPLC: Ultraperformance liquid chromatography
VIP: Variable importance in the projection
WHO: World health organization
α-MCA: α-Muricholic acid
