# Supplementary figures and images for: Dynamic alterations and potential roles of gut microbiota and metabolites in Angiostrongylus cantonensis-infected mice and rats
Source: Infect Dis Poverty. 2026 Jul 2;15:74. doi: 10.1186/s40249-026-01436-7 (PMC13326367; doi:10.1186/s40249-026-01436-7)

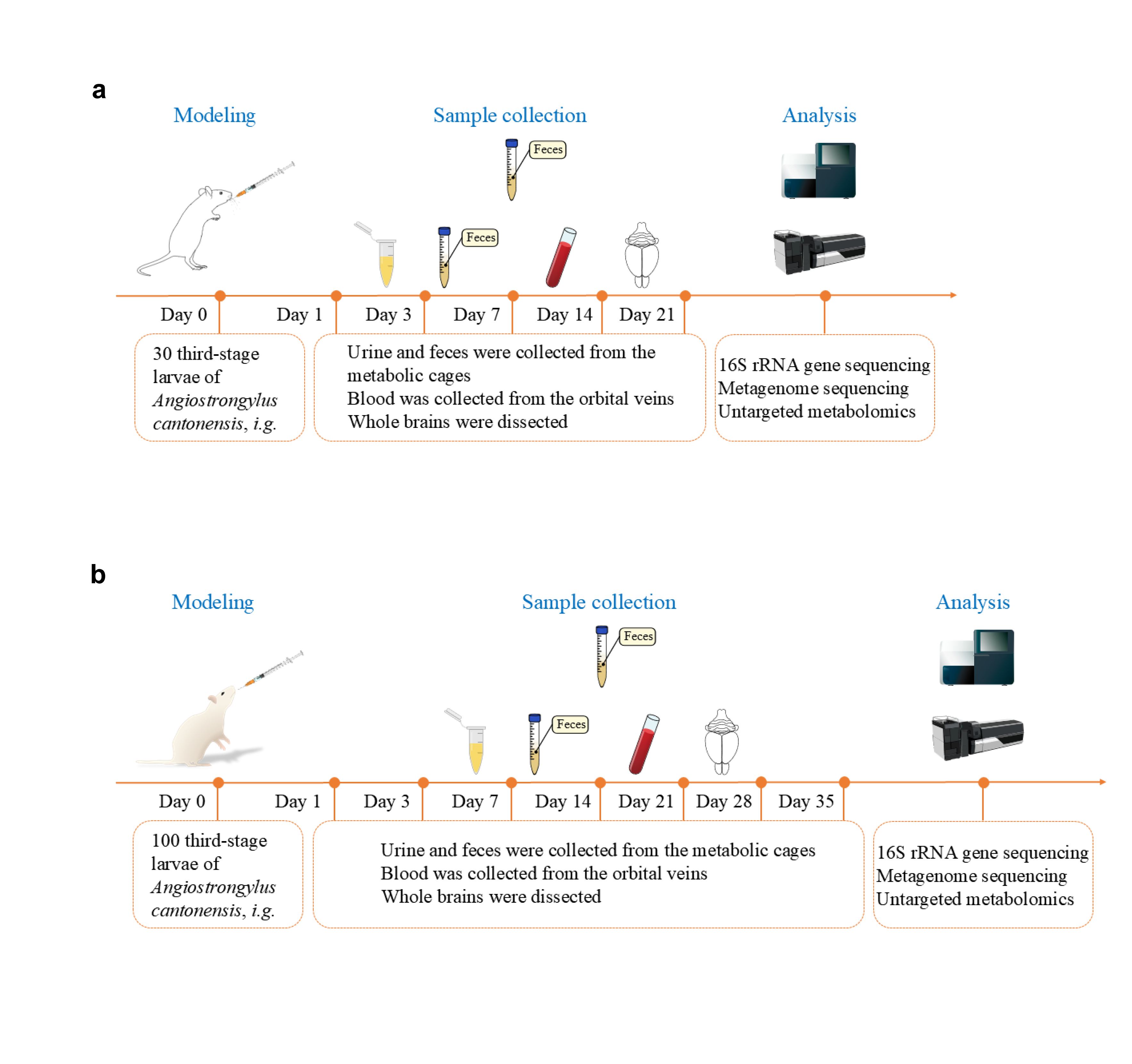

Supplement: Supplementary file 1 — Additional file1 [file 40249_2026_1436_MOESM1_ESM.tif]

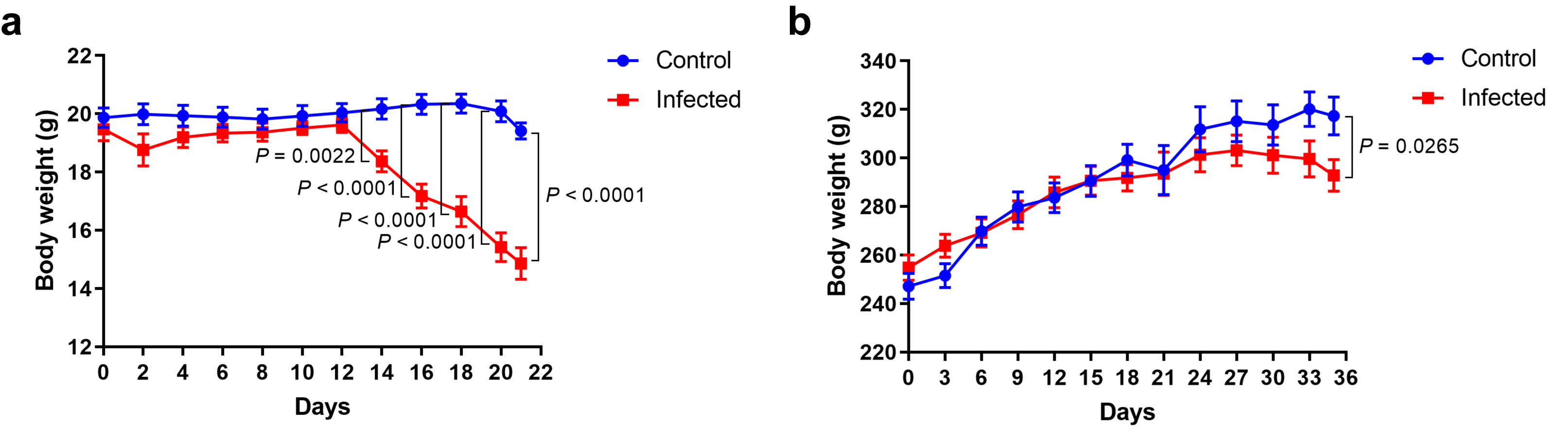

Supplement: Supplementary file 3 — Additional file3 [file 40249_2026_1436_MOESM3_ESM.tif]

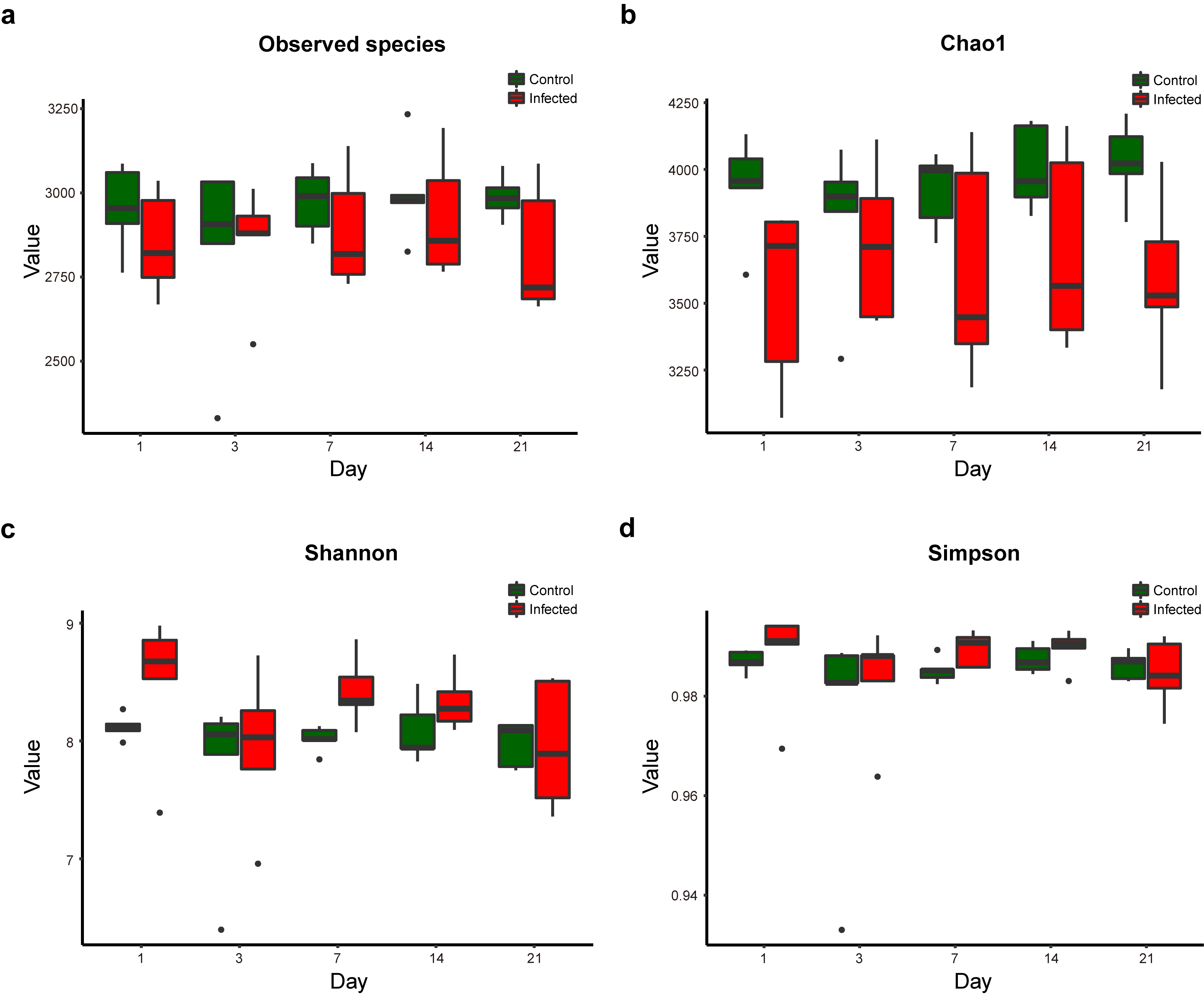

Supplement: Supplementary file 4 — Additional file4 [file 40249_2026_1436_MOESM4_ESM.tif]

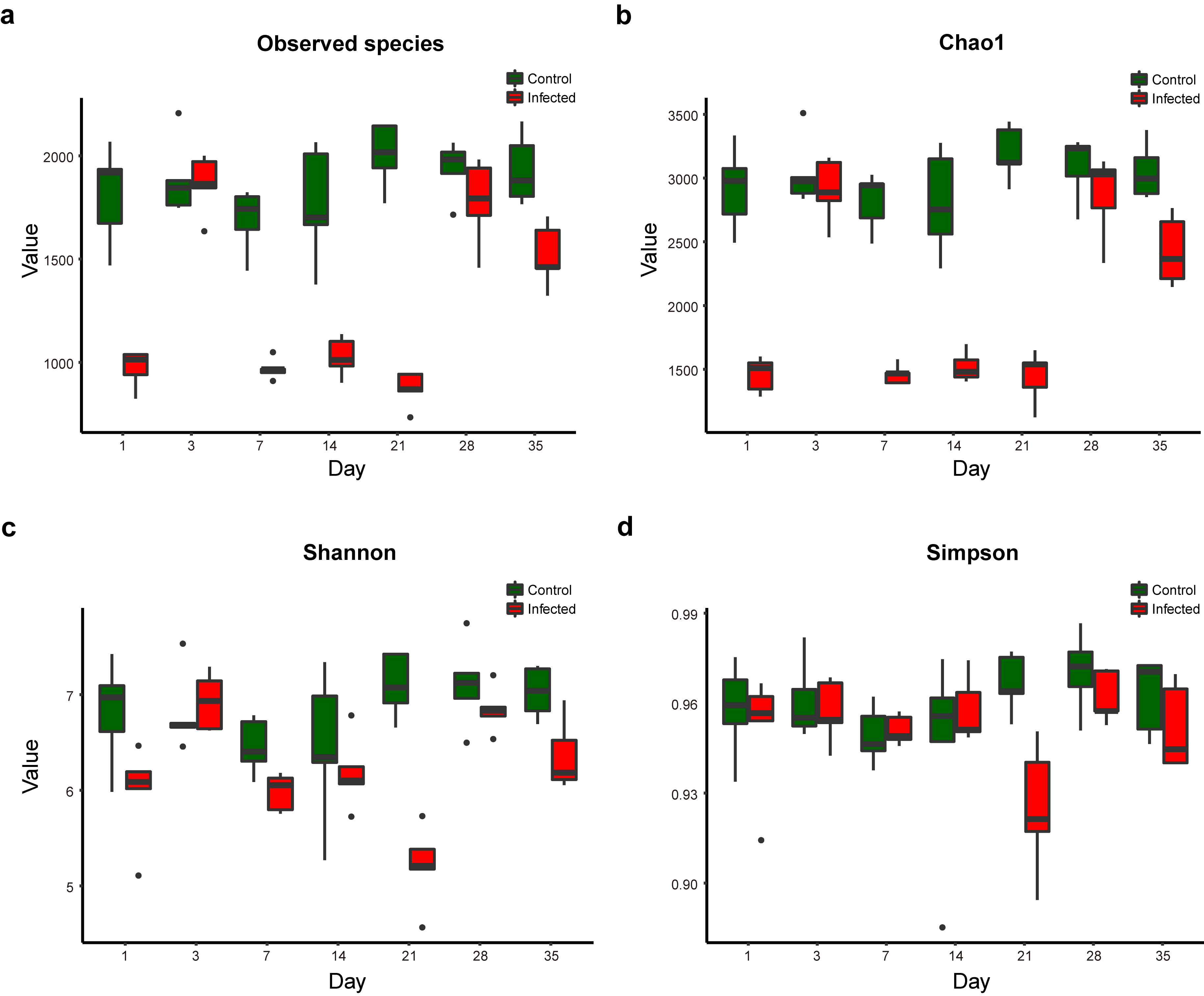

Supplement: Supplementary file 5 — Additional file5 [file 40249_2026_1436_MOESM5_ESM.tif]

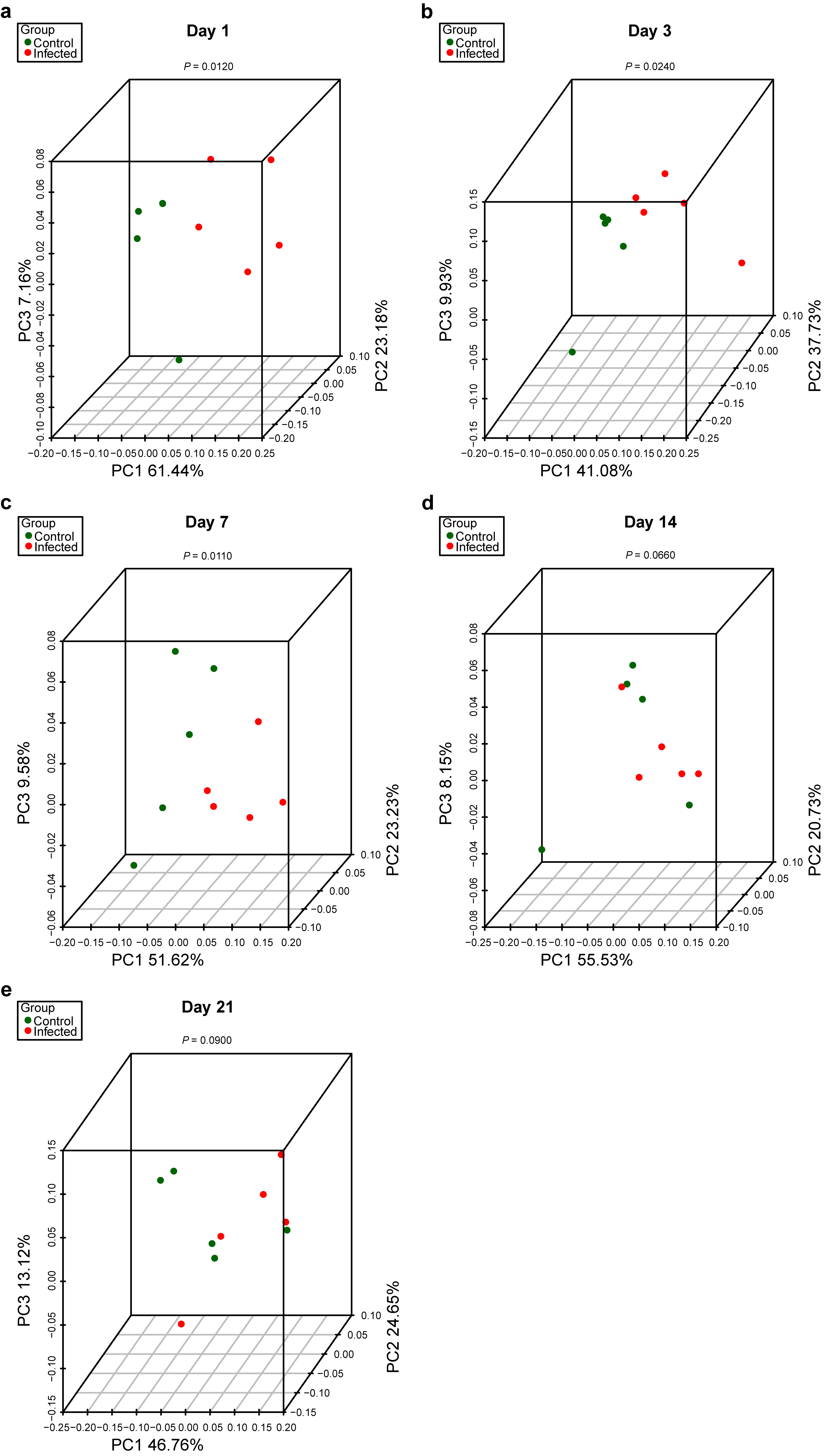

Supplement: Supplementary file 6 — Additional file6 [file 40249_2026_1436_MOESM6_ESM.tif]

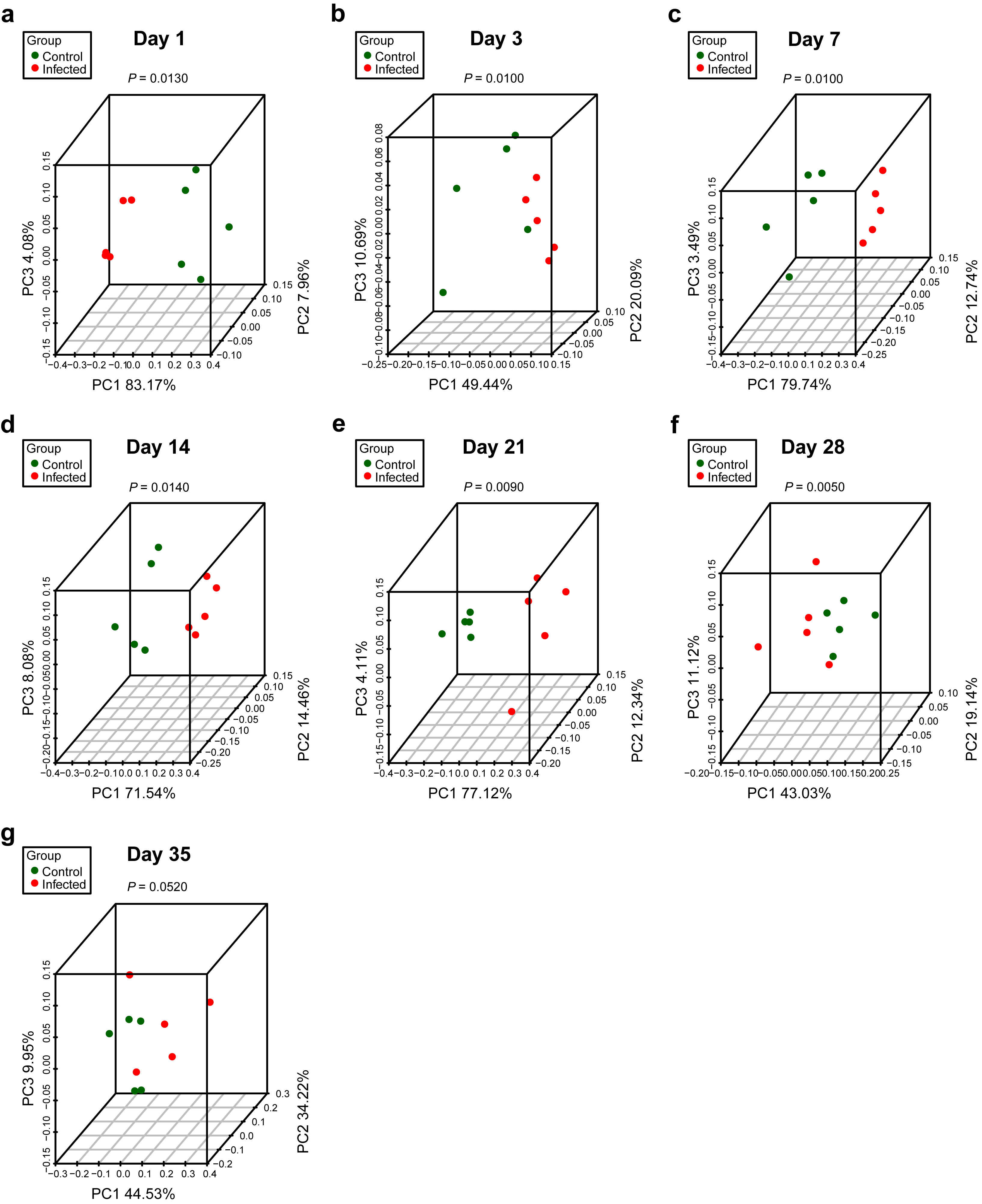

Supplement: Supplementary file 7 — Additional file7 [file 40249_2026_1436_MOESM7_ESM.tif]

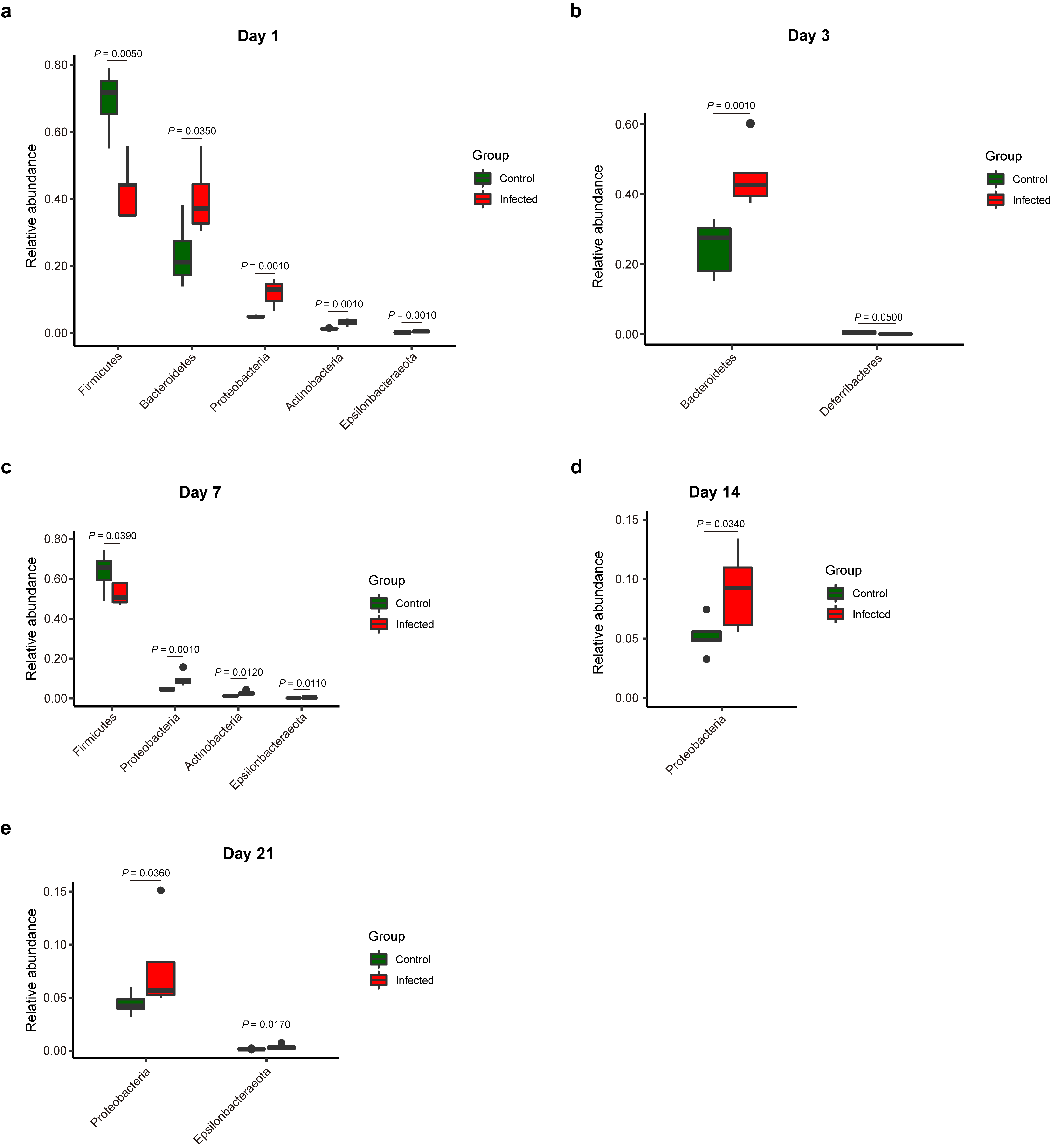

Supplement: Supplementary file 8 — Additional file8 [file 40249_2026_1436_MOESM8_ESM.tif]

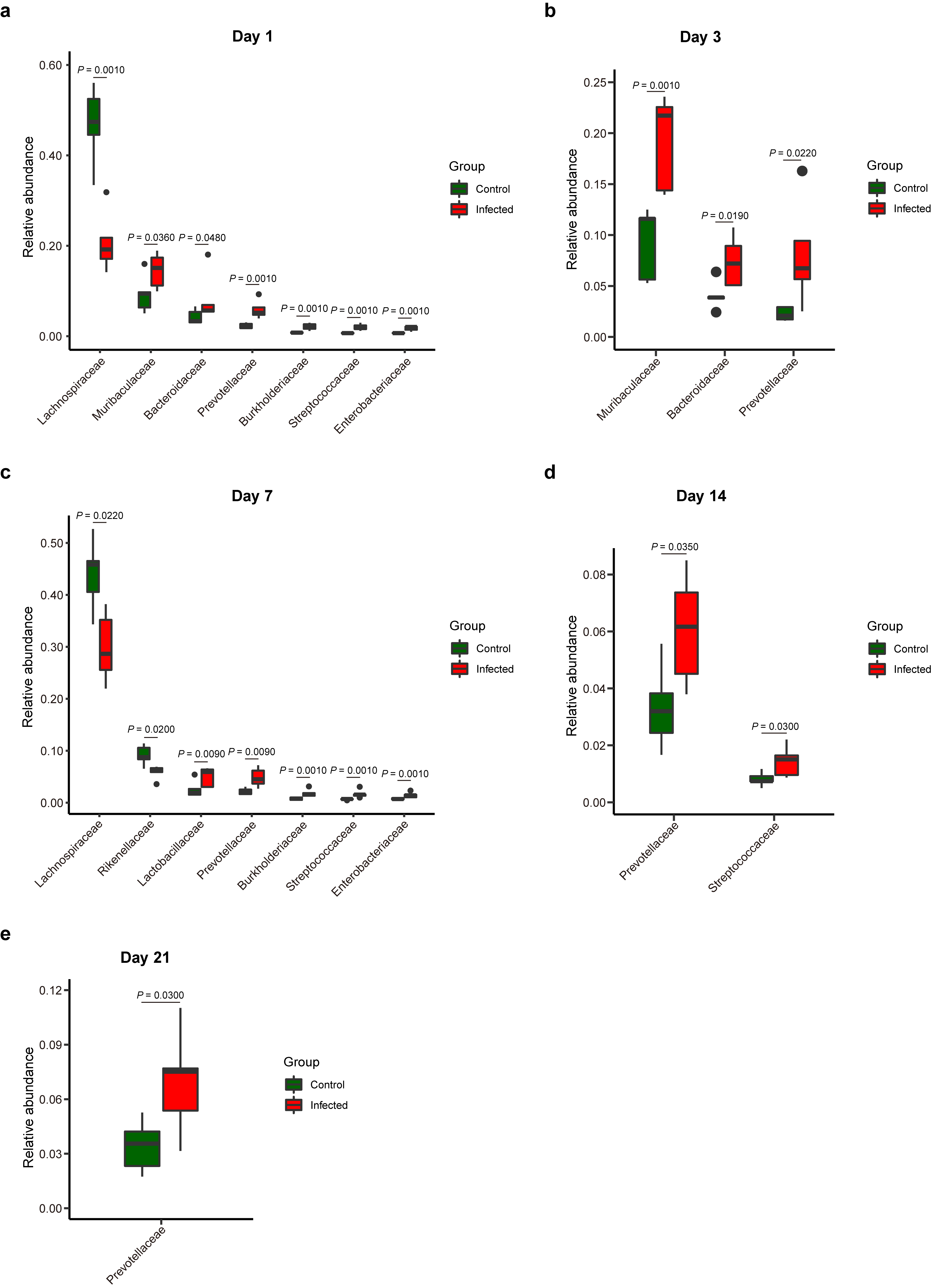

Supplement: Supplementary file 9 — Additional file9 [file 40249_2026_1436_MOESM9_ESM.tif]

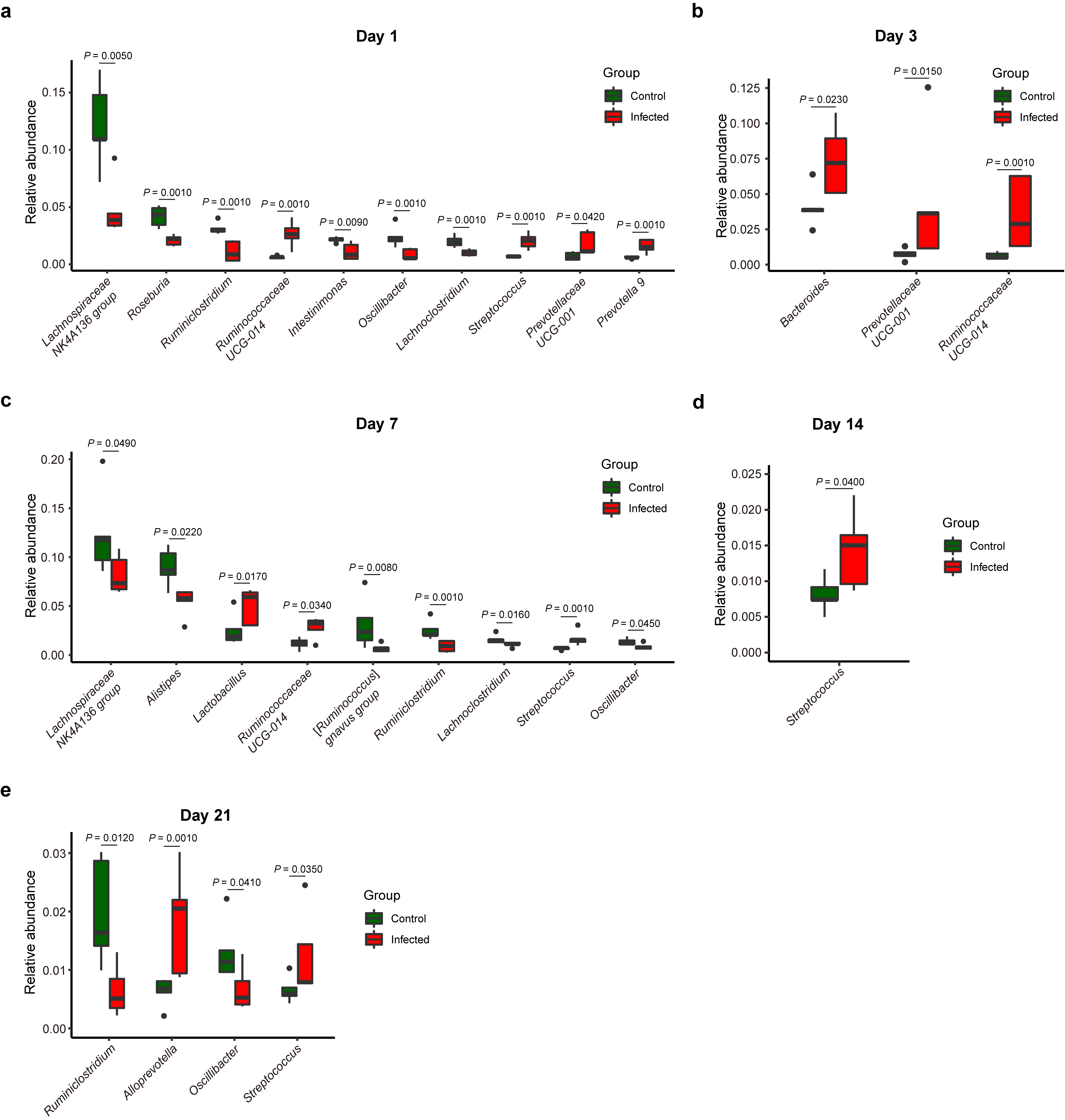

Supplement: Supplementary file 10 — Additional file10 [file 40249_2026_1436_MOESM10_ESM.tif]

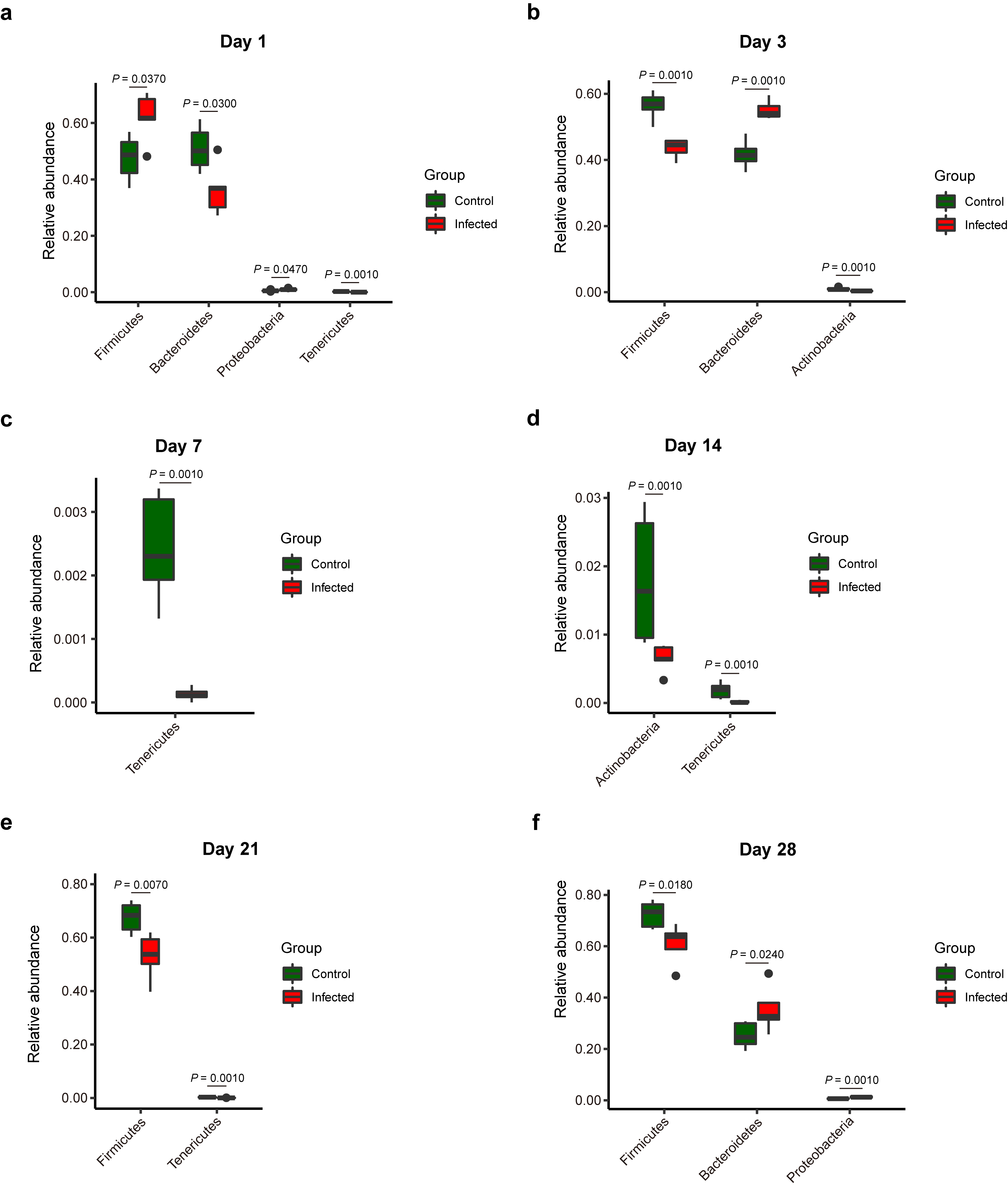

Supplement: Supplementary file 11 — Additional file11 [file 40249_2026_1436_MOESM11_ESM.tif]

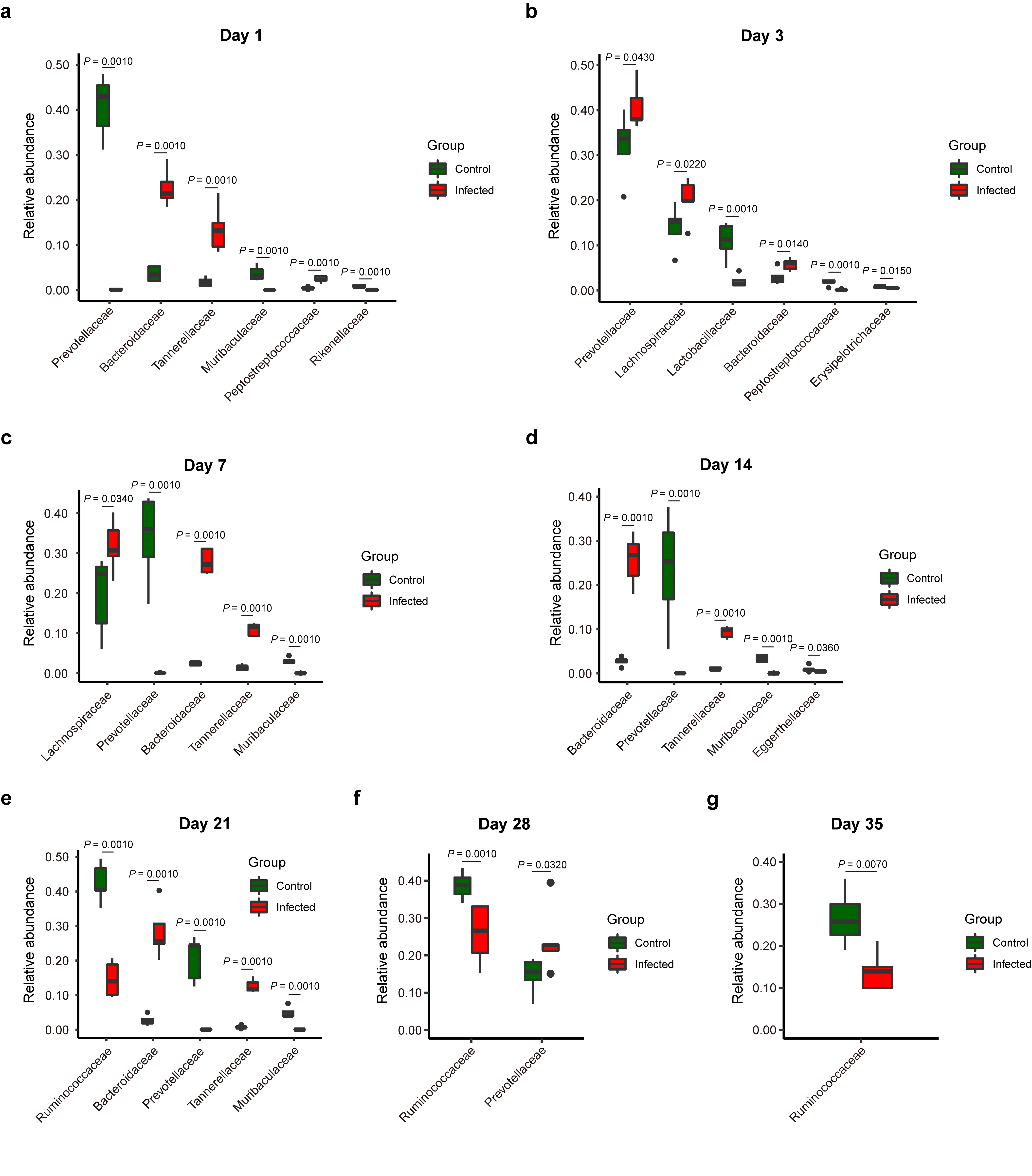

Supplement: Supplementary file 12 — Additional file12 [file 40249_2026_1436_MOESM12_ESM.tif]

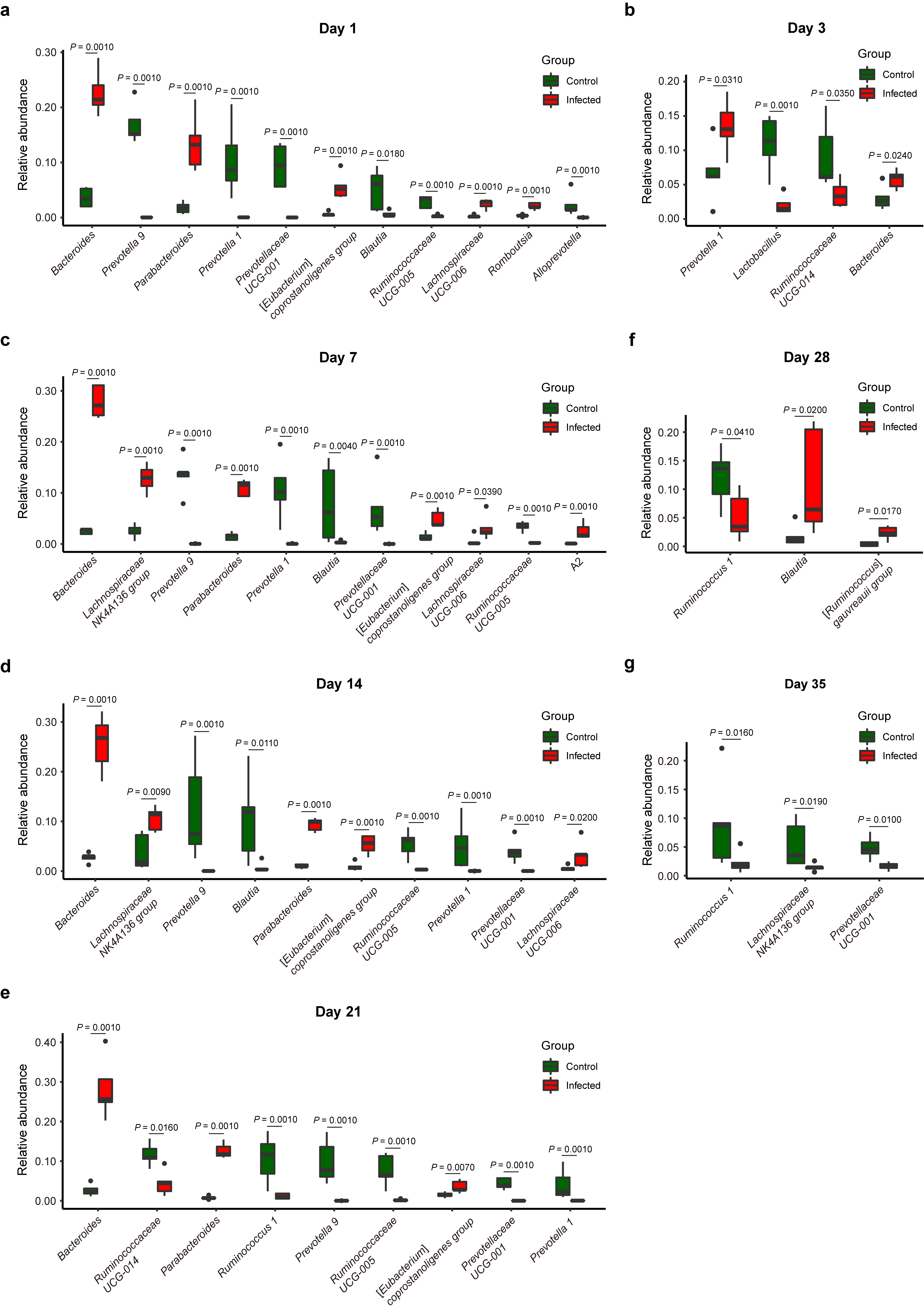

Supplement: Supplementary file 13 — Additional file13 [file 40249_2026_1436_MOESM13_ESM.tif]

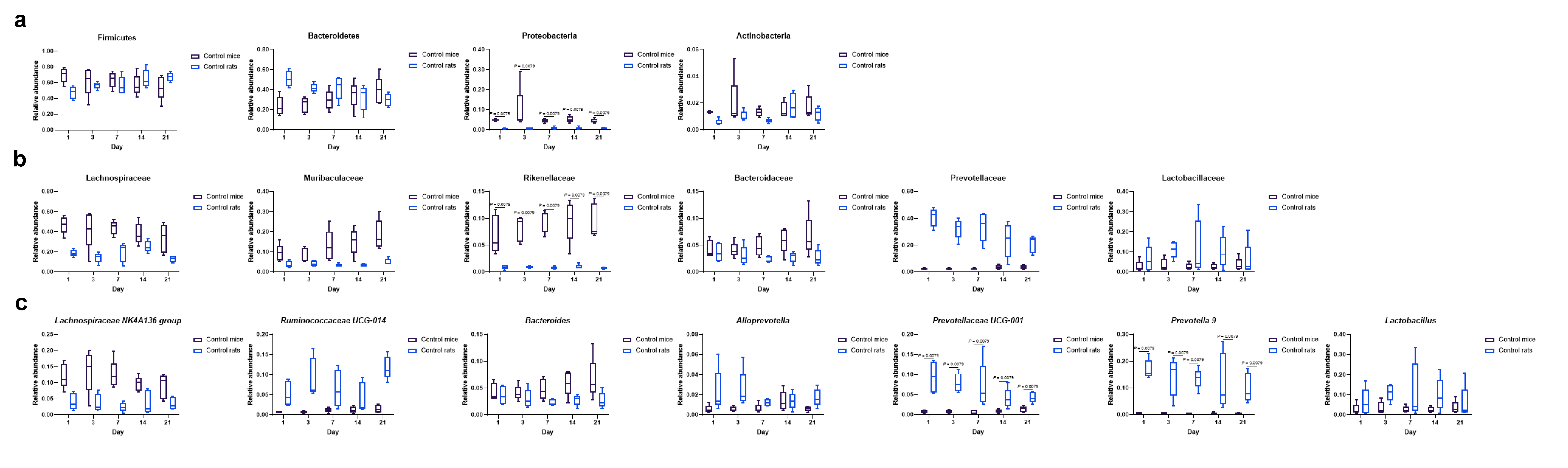

Supplement: Supplementary file 14 — Additional file14 [file 40249_2026_1436_MOESM14_ESM.tif]

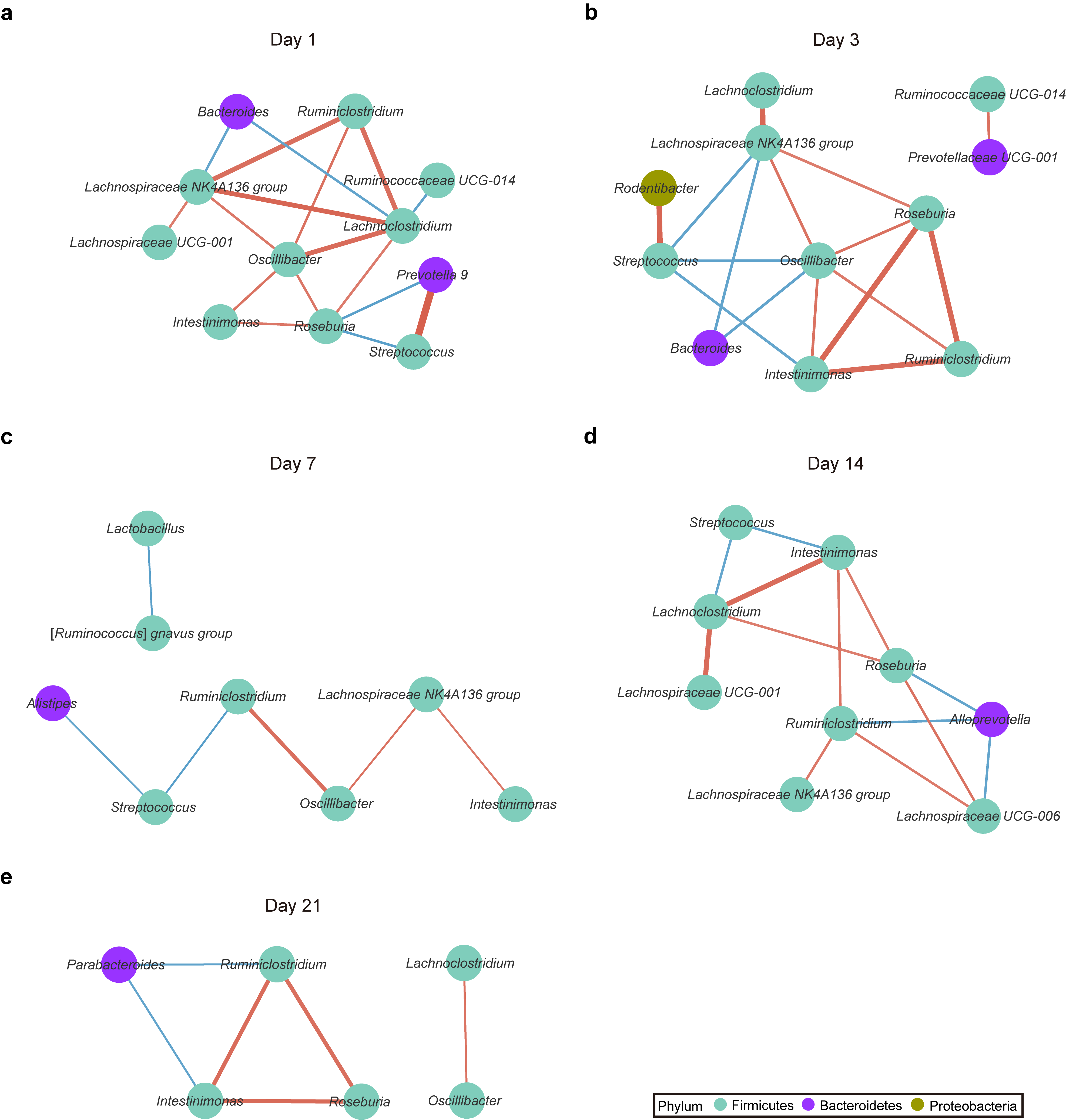

Supplement: Supplementary file 15 — Additional file15 [file 40249_2026_1436_MOESM15_ESM.tif]

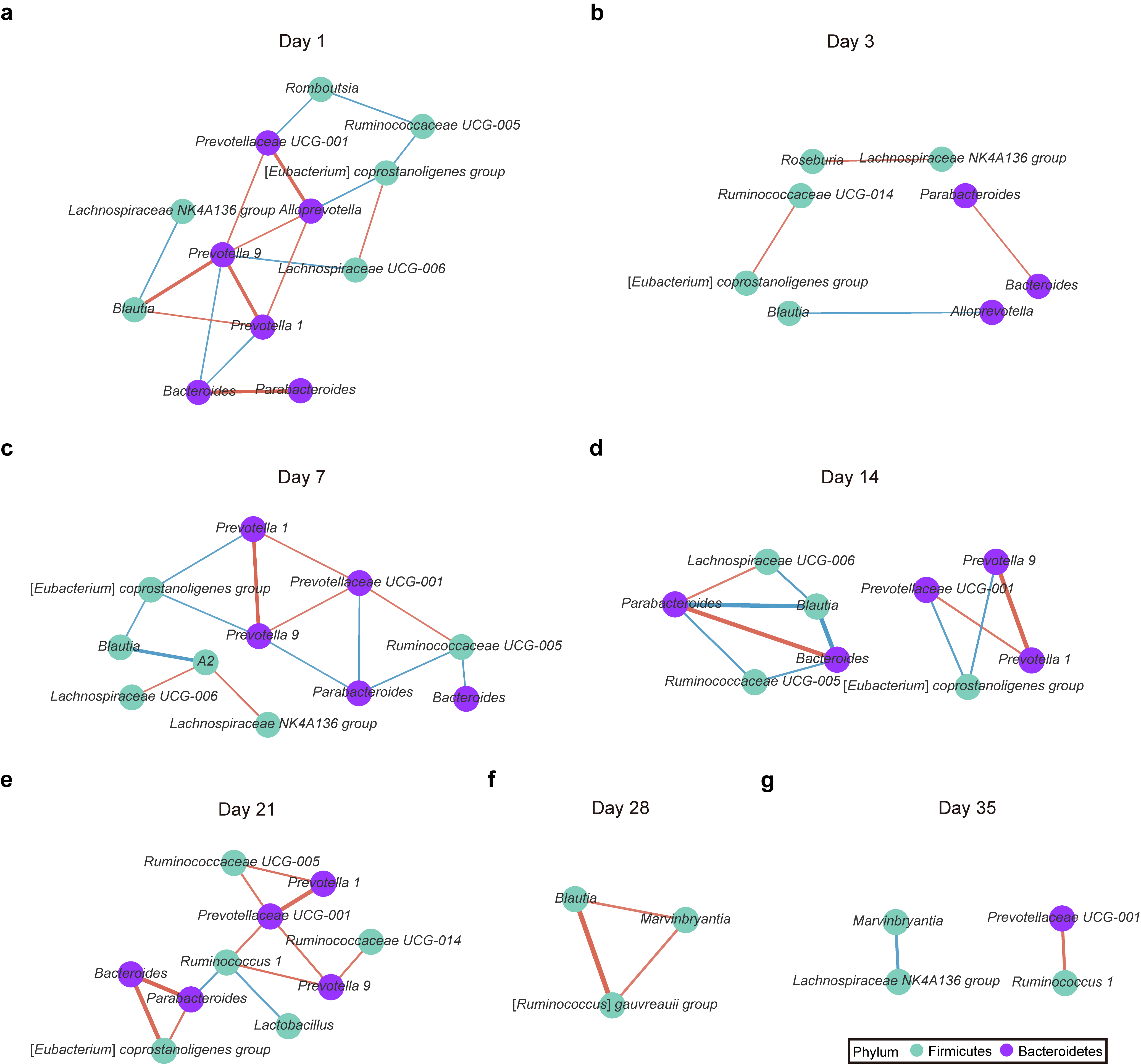

Supplement: Supplementary file 16 — Additional file16 [file 40249_2026_1436_MOESM16_ESM.tif]

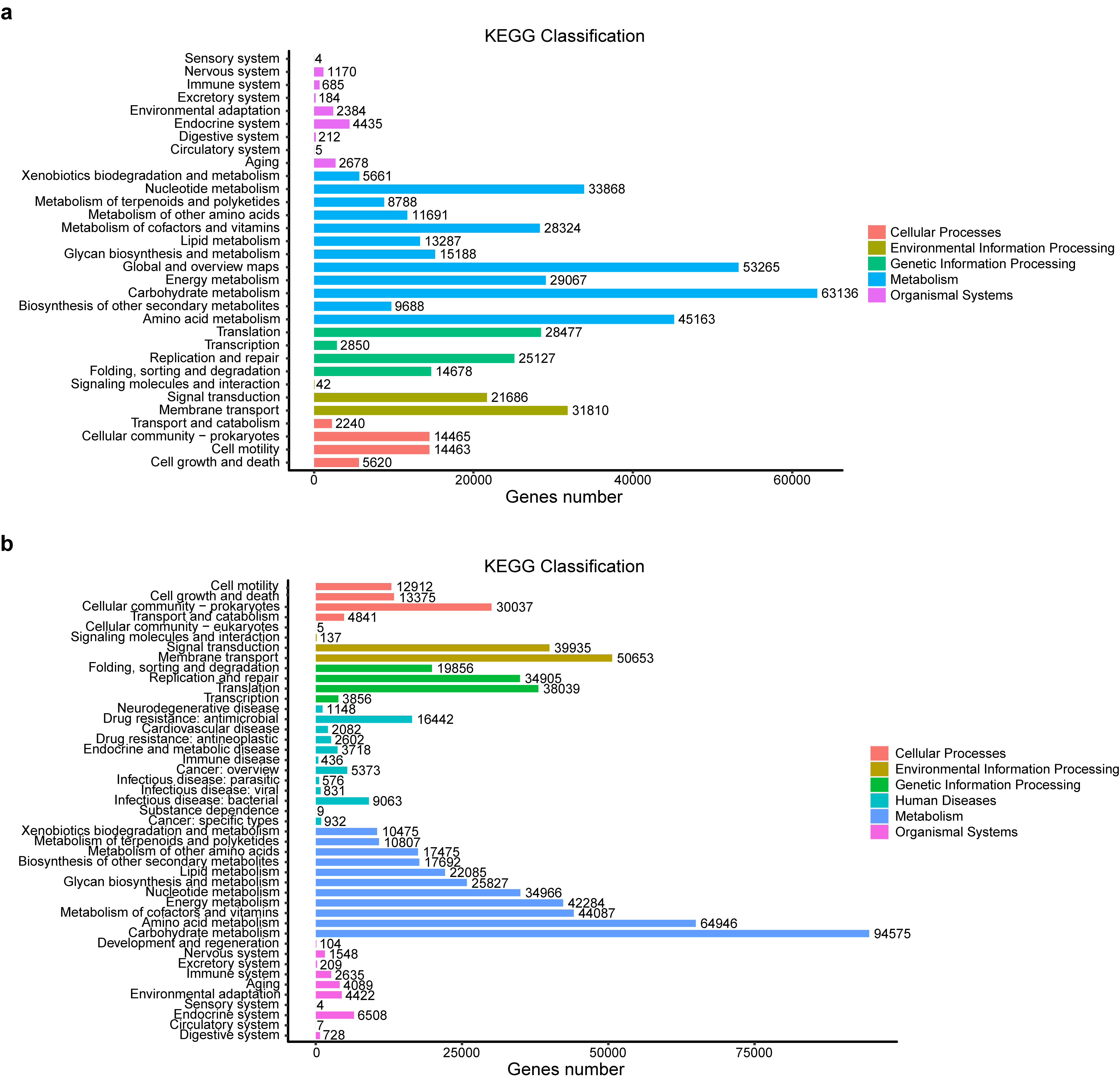

Supplement: Supplementary file 17 — Additional file17 [file 40249_2026_1436_MOESM17_ESM.tif]

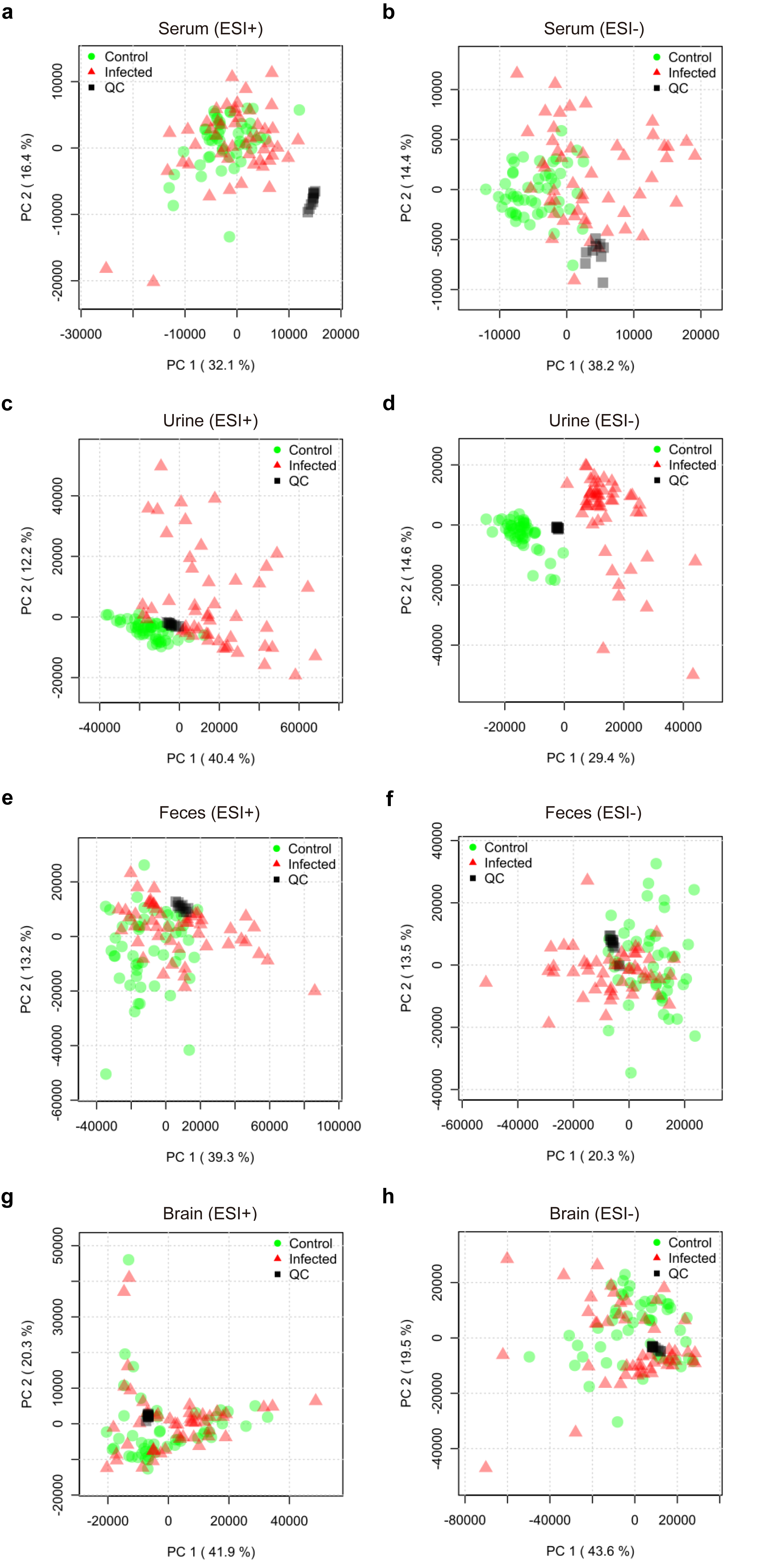

Supplement: Supplementary file 18 — Additional file18 [file 40249_2026_1436_MOESM18_ESM.tif]

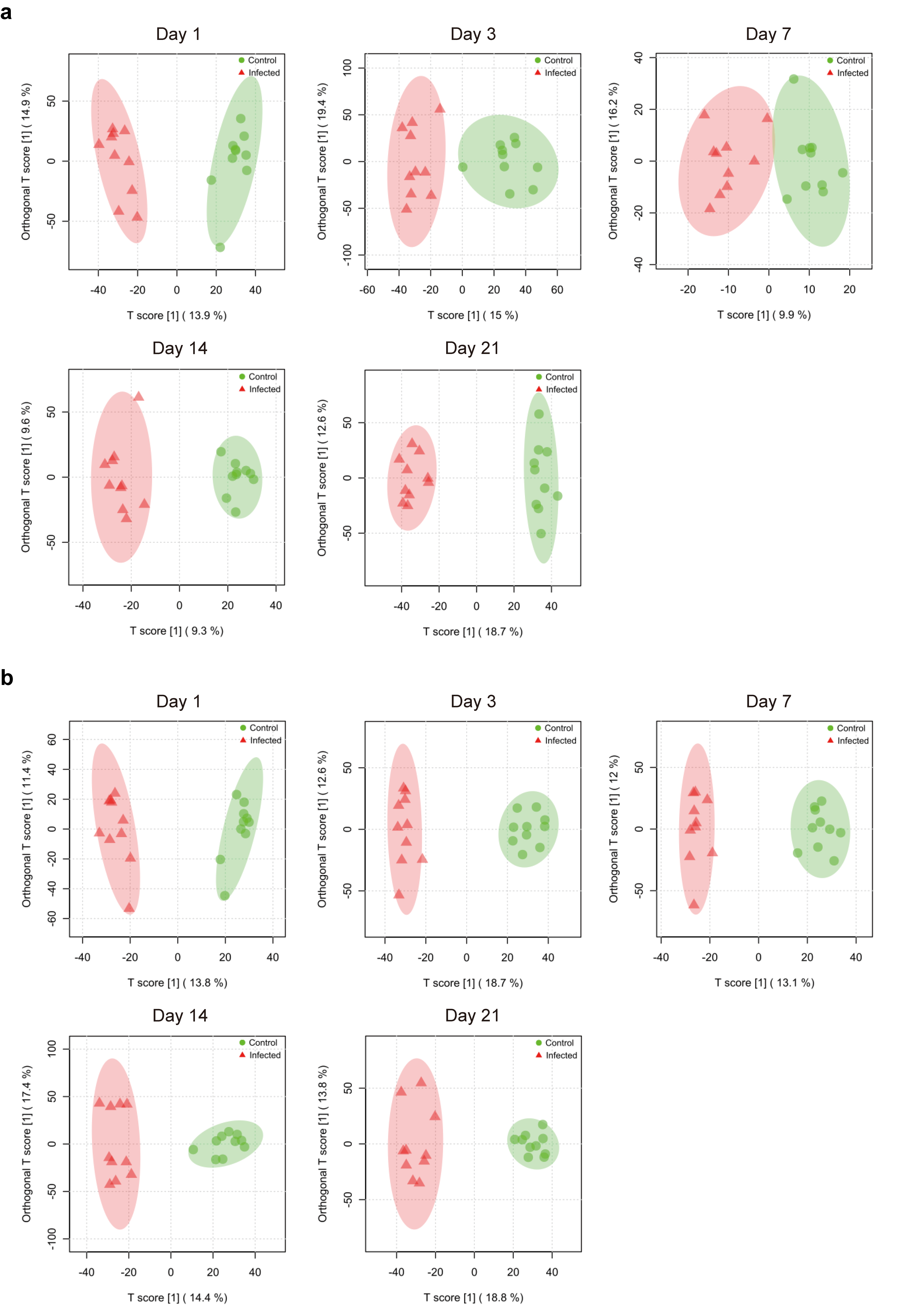

Supplement: Supplementary file 19 — Additional file19 [file 40249_2026_1436_MOESM19_ESM.tif]

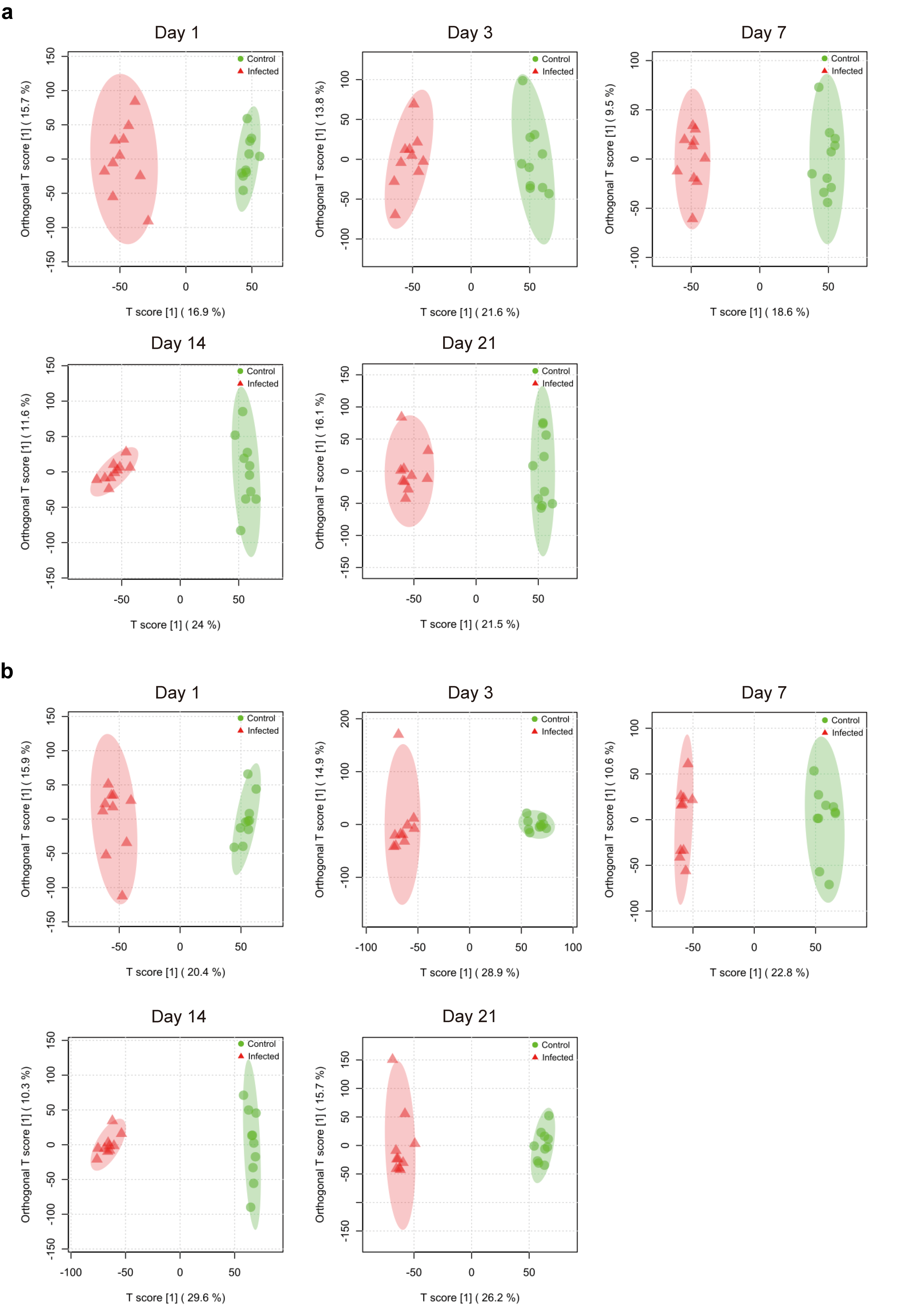

Supplement: Supplementary file 20 — Additional file20 [file 40249_2026_1436_MOESM20_ESM.tif]

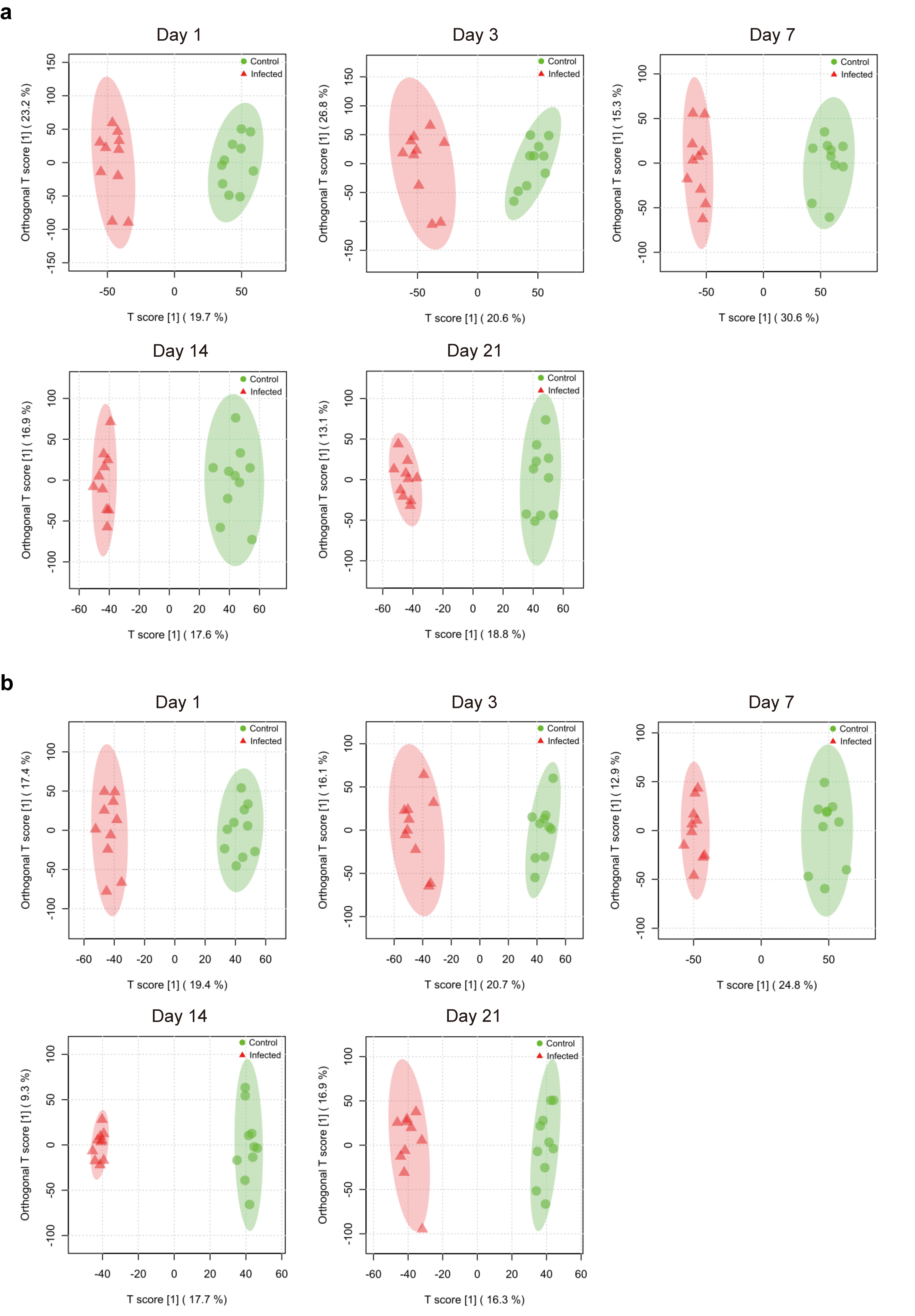

Supplement: Supplementary file 21 — Additional file21 [file 40249_2026_1436_MOESM21_ESM.tif]

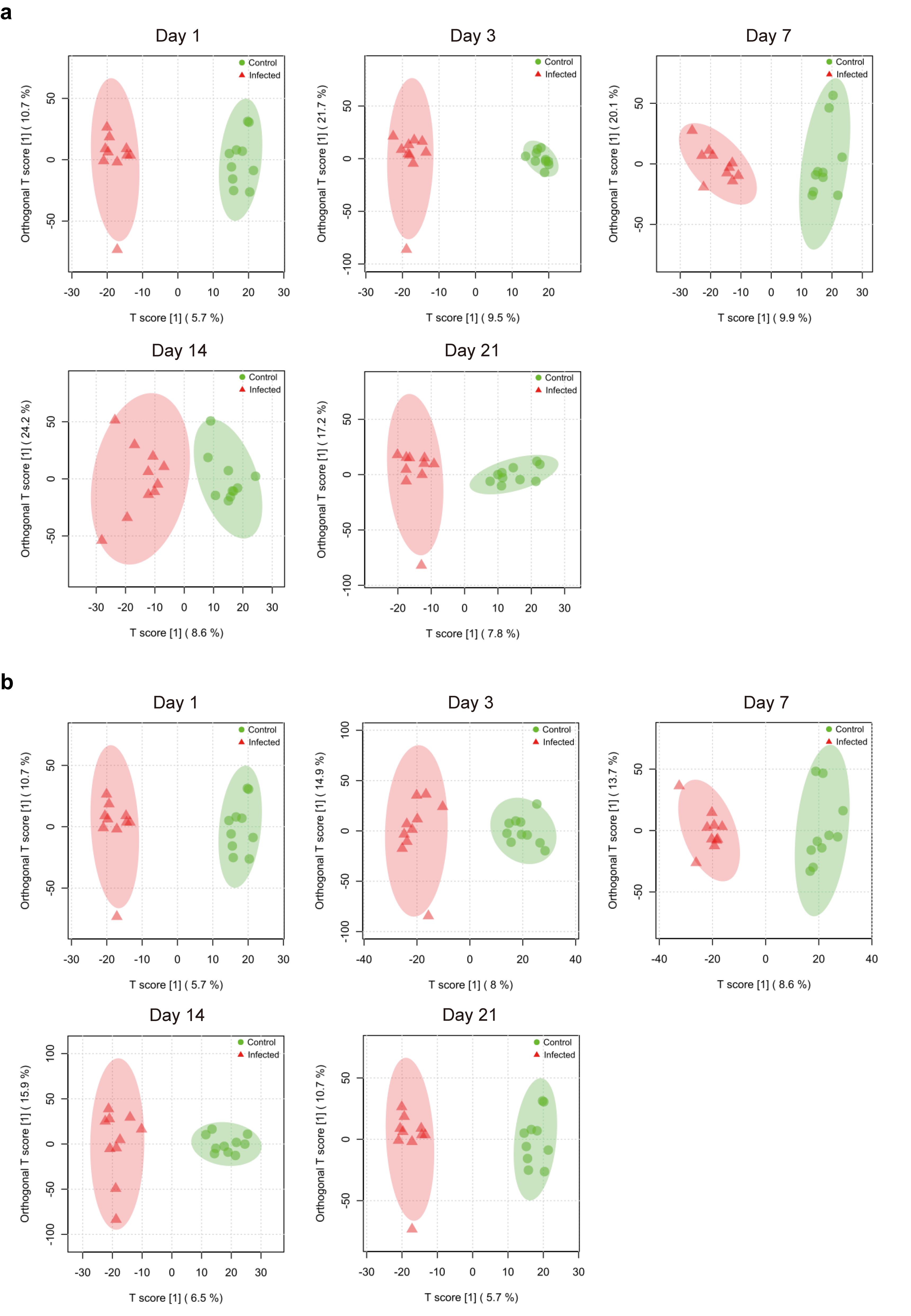

Supplement: Supplementary file 22 — Additional file22 [file 40249_2026_1436_MOESM22_ESM.tif]

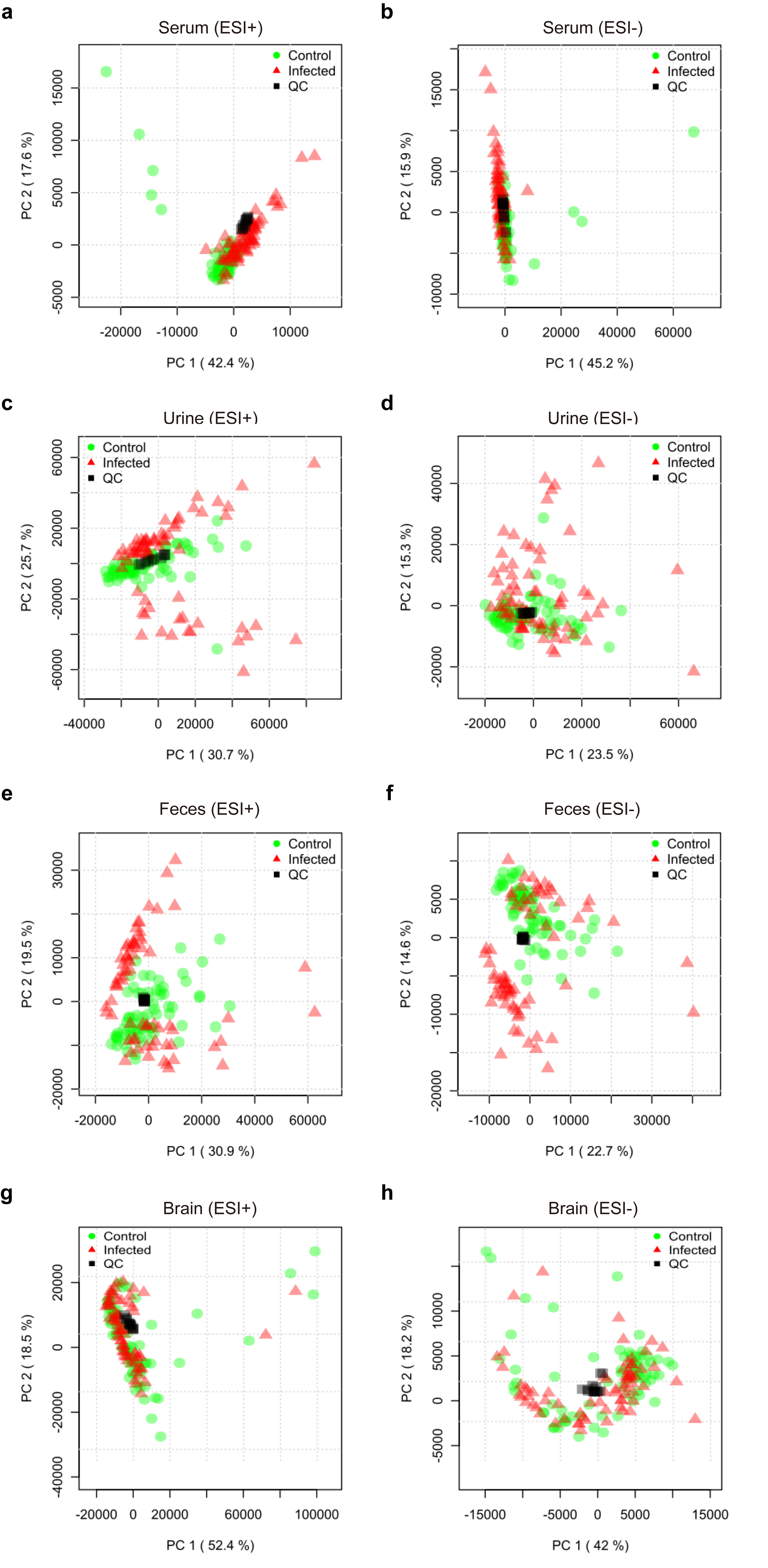

Supplement: Supplementary file 24 — Additional file24 [file 40249_2026_1436_MOESM24_ESM.tif]

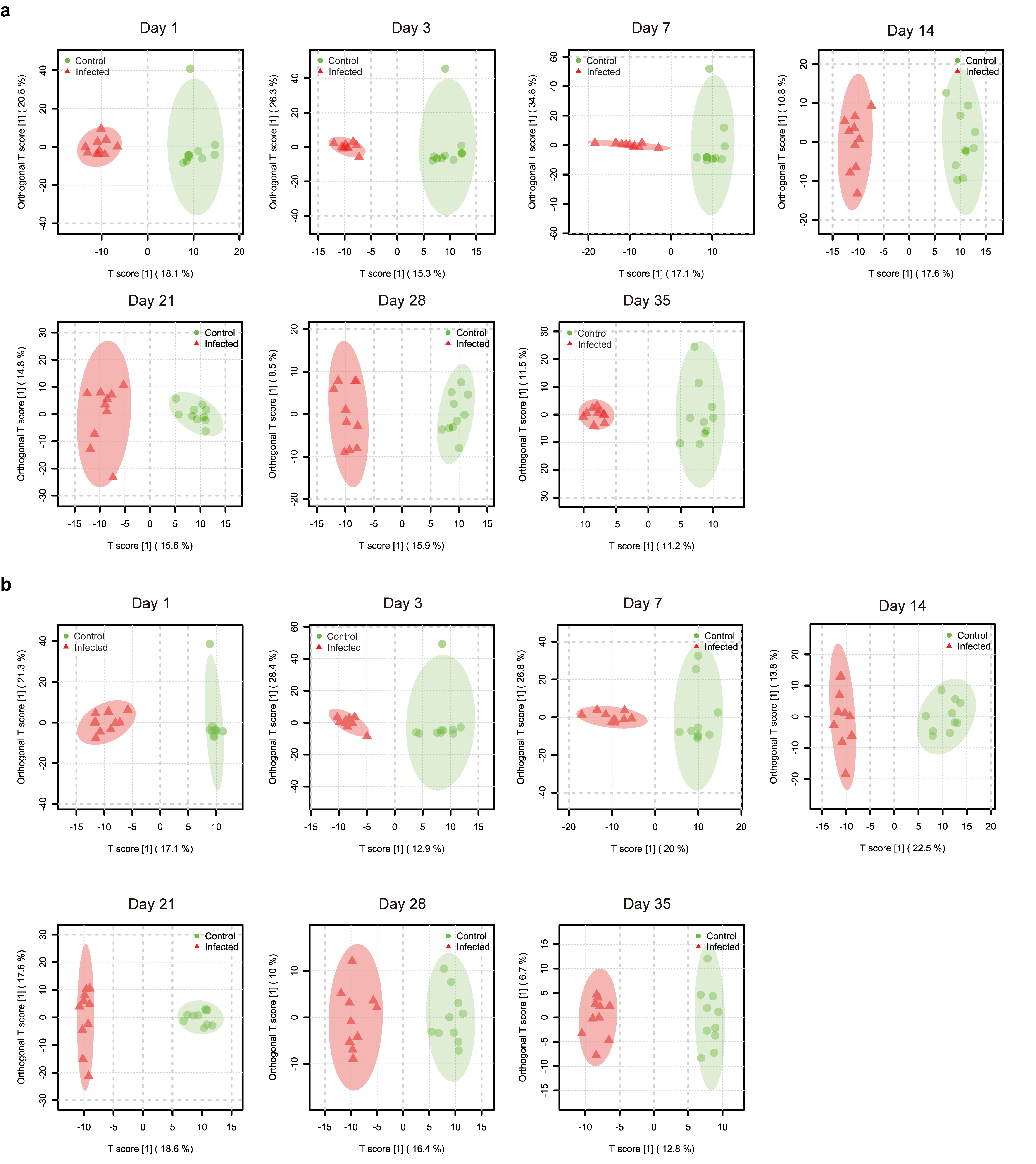

Supplement: Supplementary file 25 — Additional file25 [file 40249_2026_1436_MOESM25_ESM.tif]

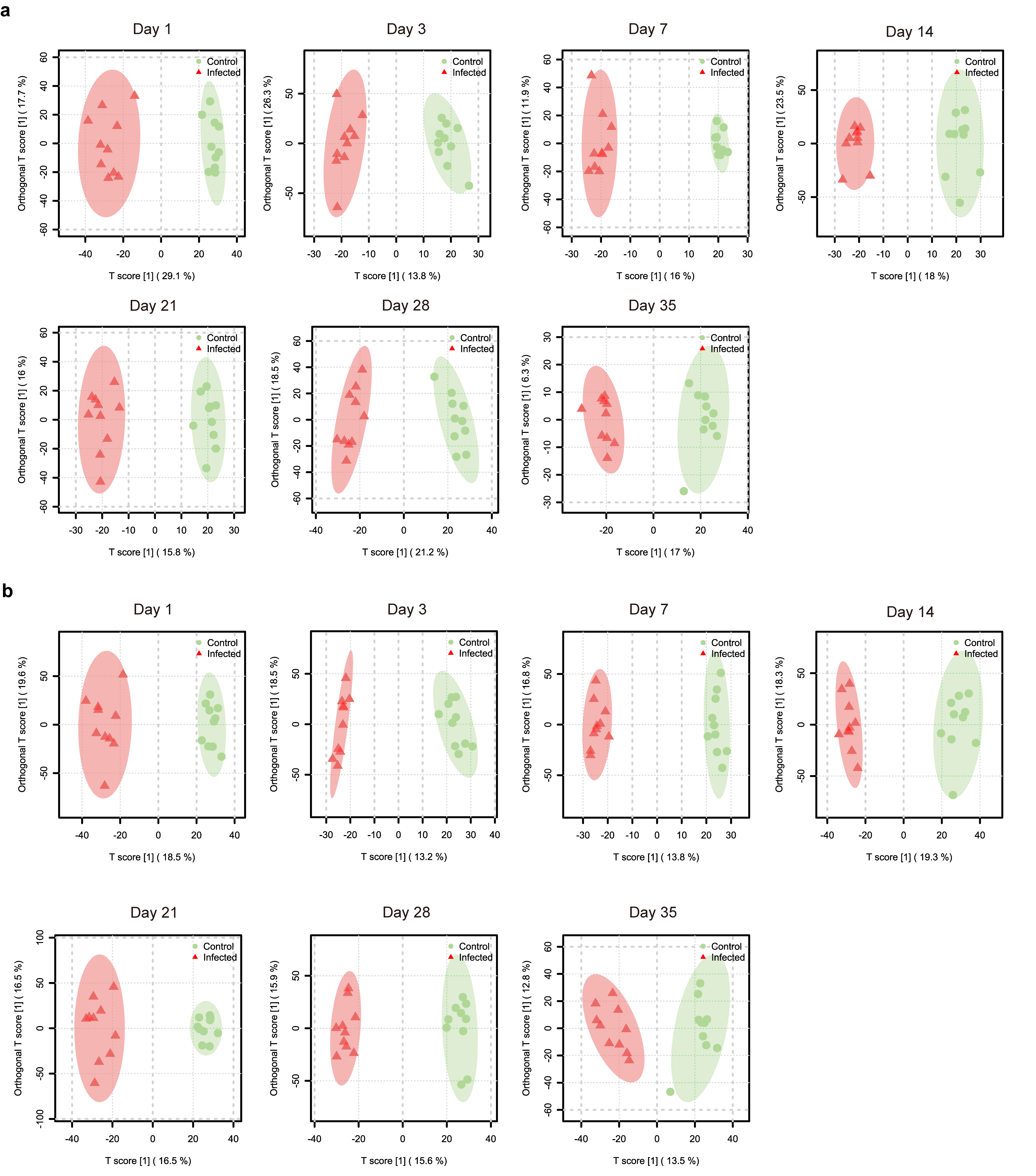

Supplement: Supplementary file 26 — Additional file26 [file 40249_2026_1436_MOESM26_ESM.tif]

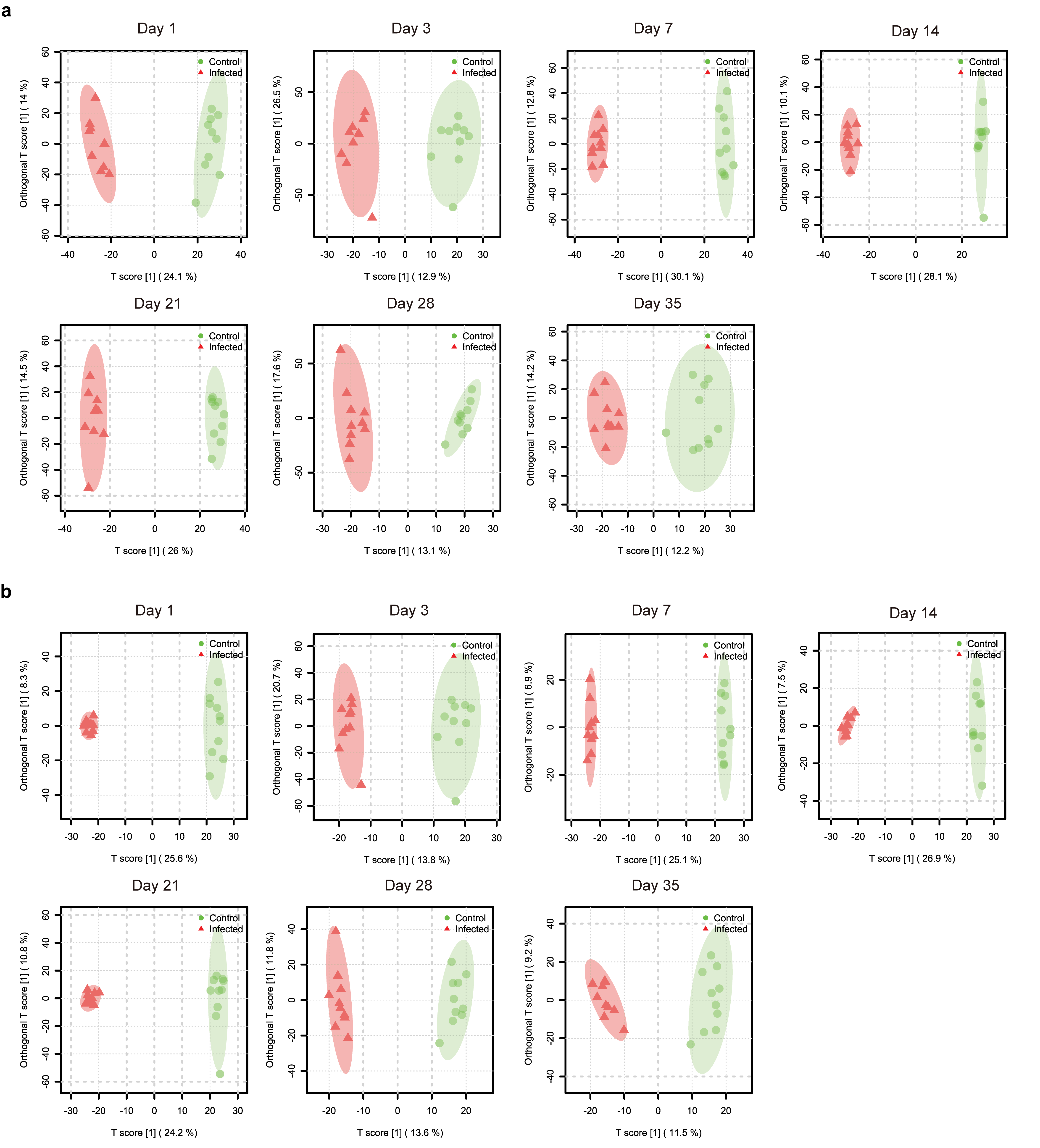

Supplement: Supplementary file 27 — Additional file27 [file 40249_2026_1436_MOESM27_ESM.tif]

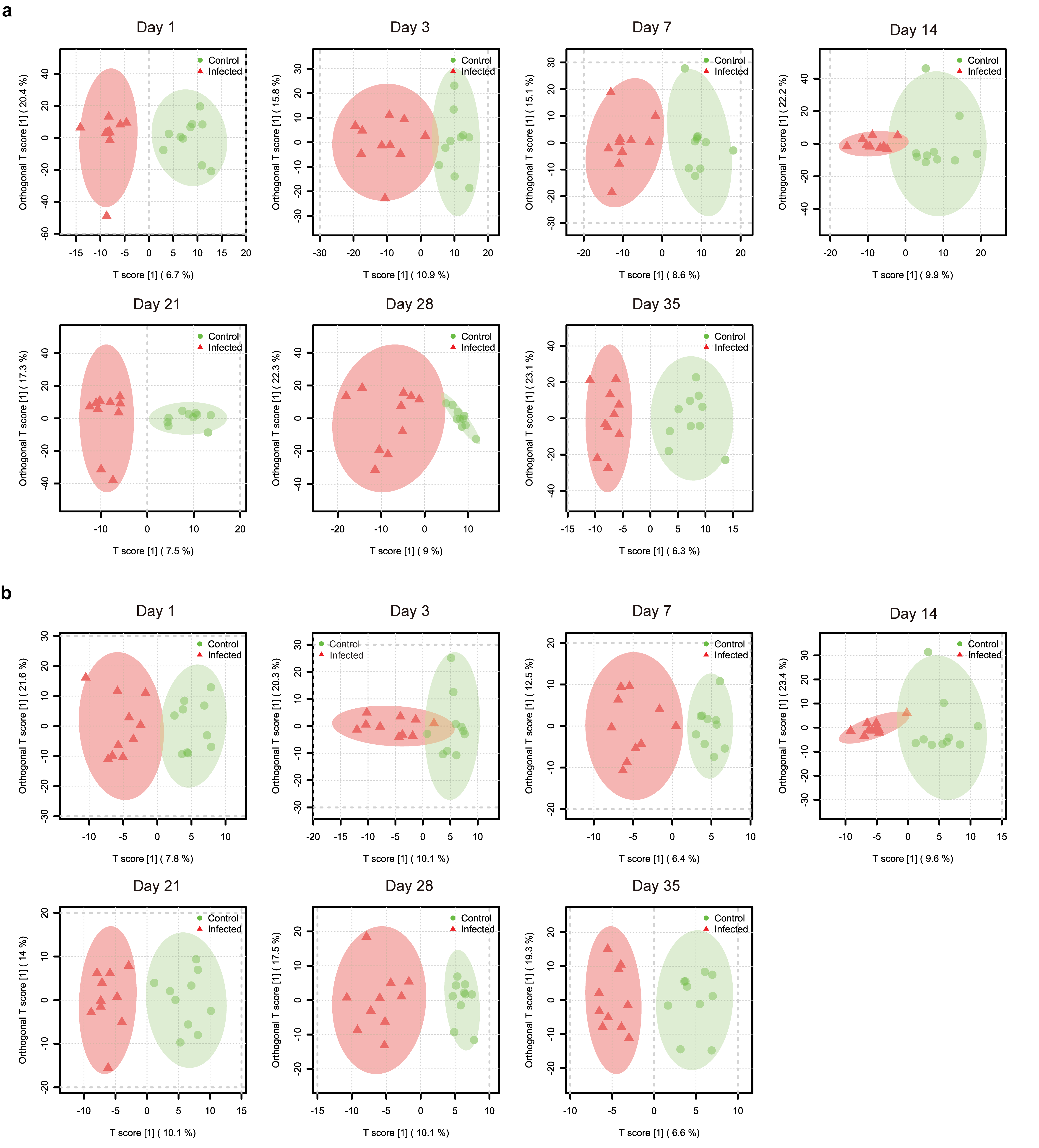

Supplement: Supplementary file 28 — Additional file28 [file 40249_2026_1436_MOESM28_ESM.tif]

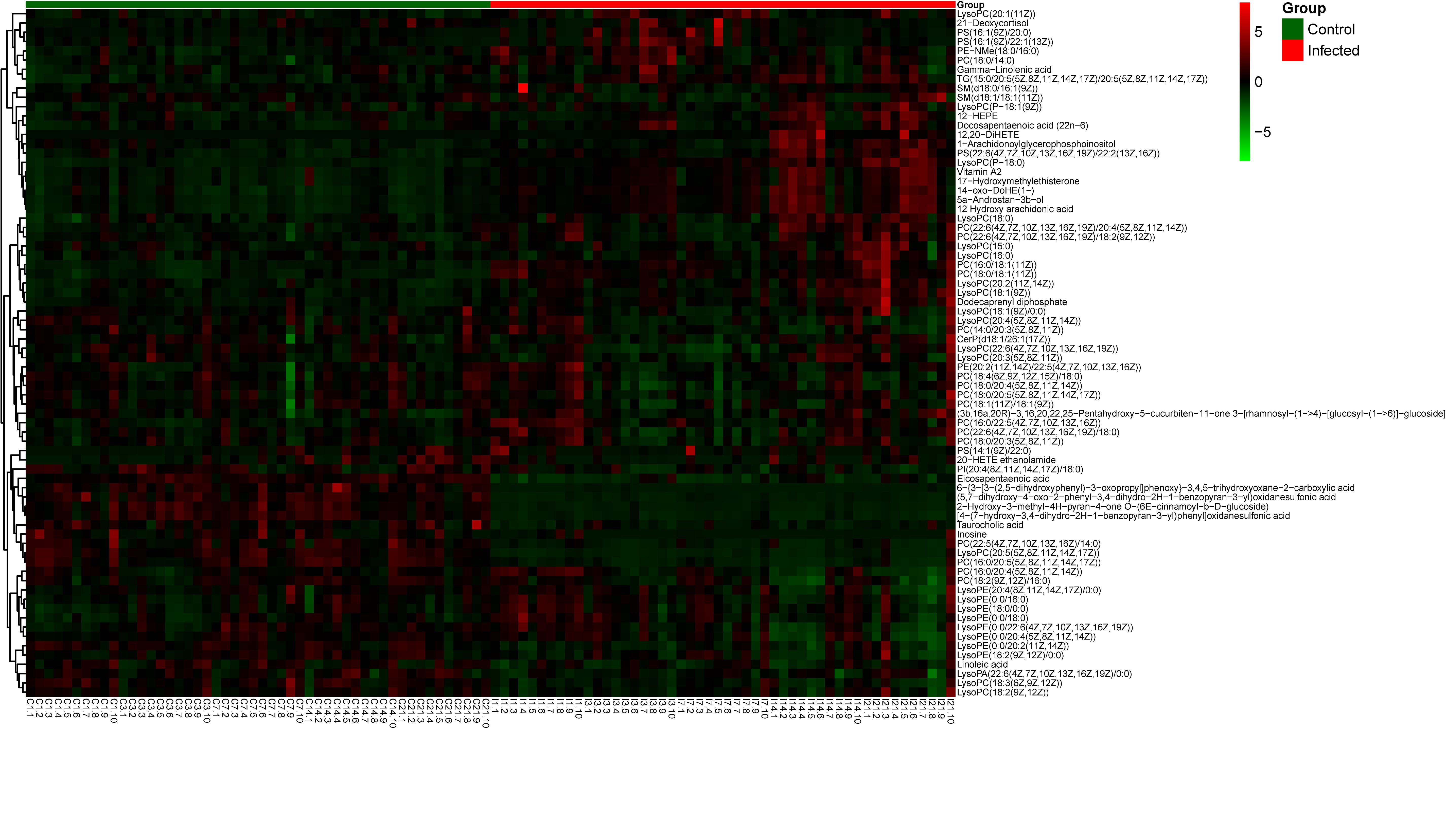

Supplement: Supplementary file 30 — Additional file30 [file 40249_2026_1436_MOESM30_ESM.tif]

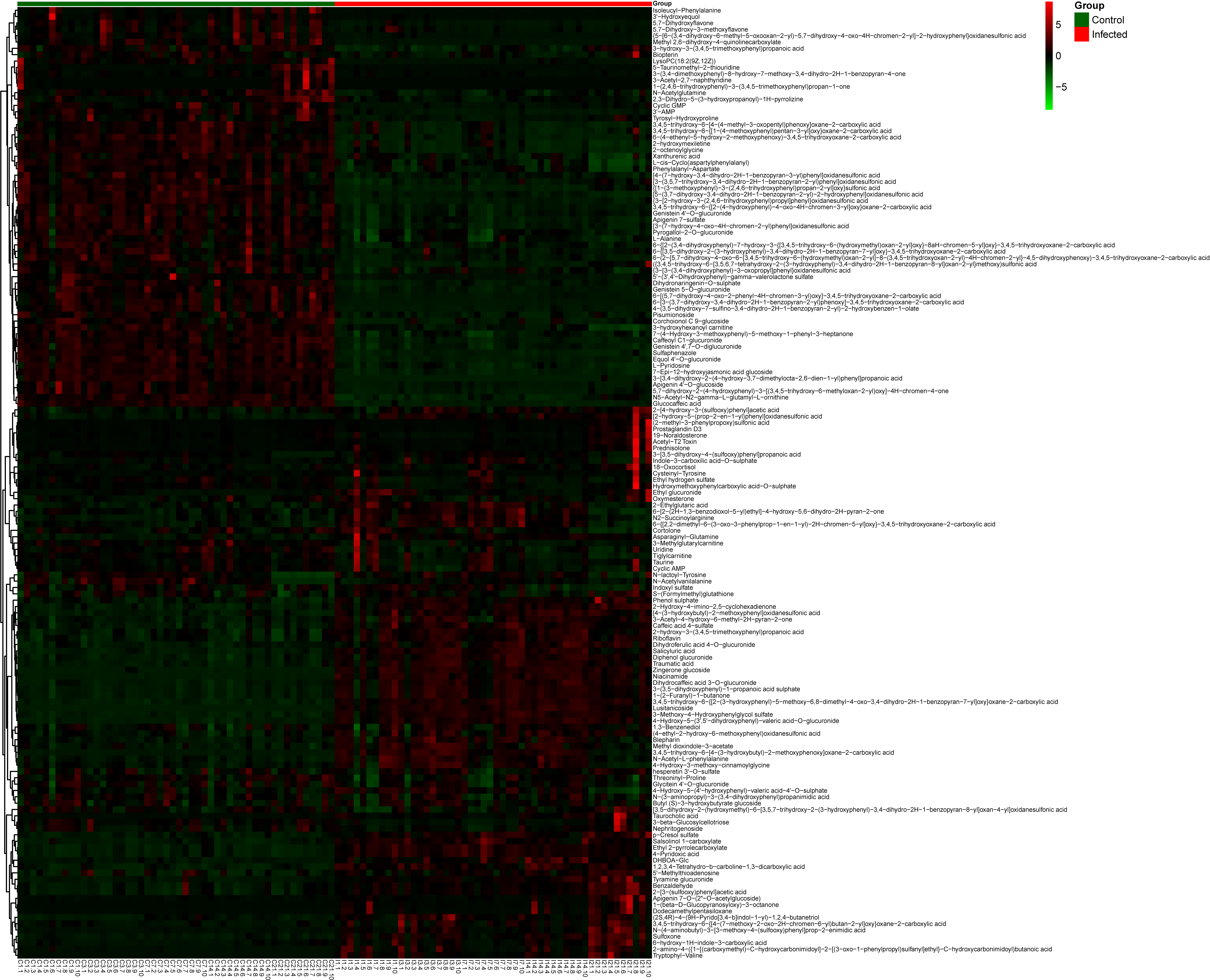

Supplement: Supplementary file 31 — Additional file31 [file 40249_2026_1436_MOESM31_ESM.tif]

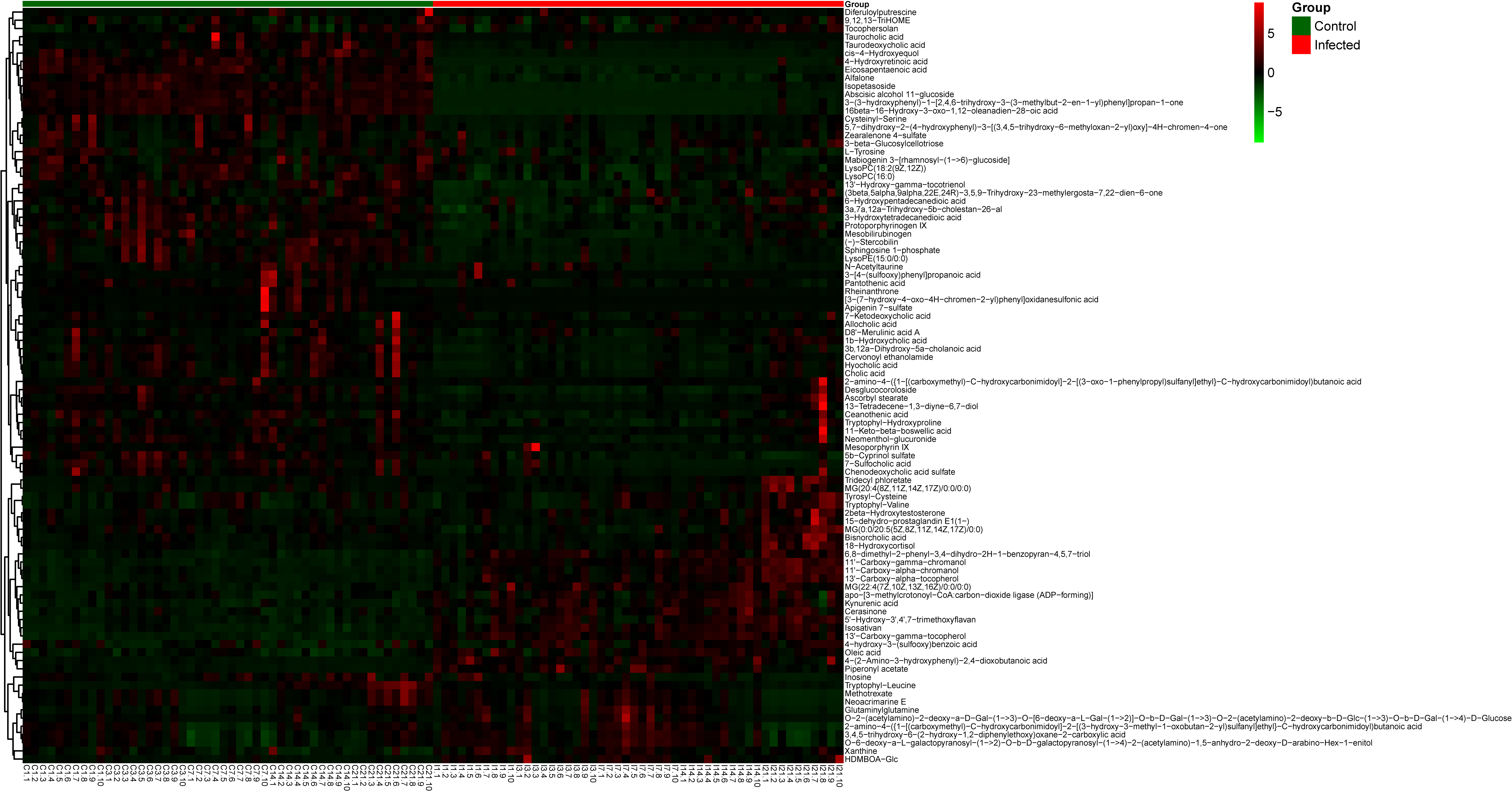

Supplement: Supplementary file 32 — Additional file32 [file 40249_2026_1436_MOESM32_ESM.tif]

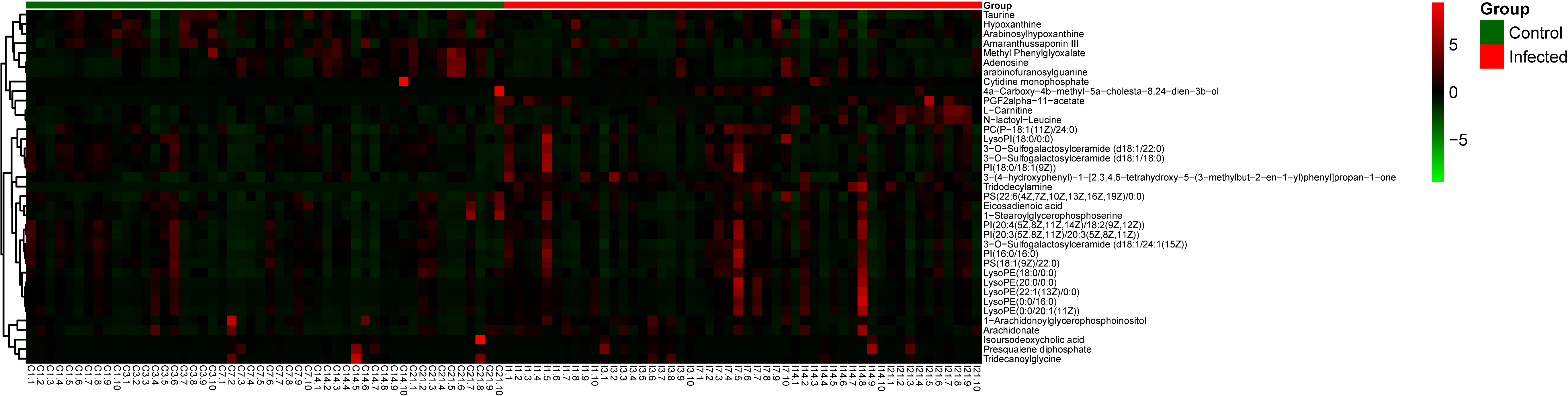

Supplement: Supplementary file 33 — Additional file33 [file 40249_2026_1436_MOESM33_ESM.tif]

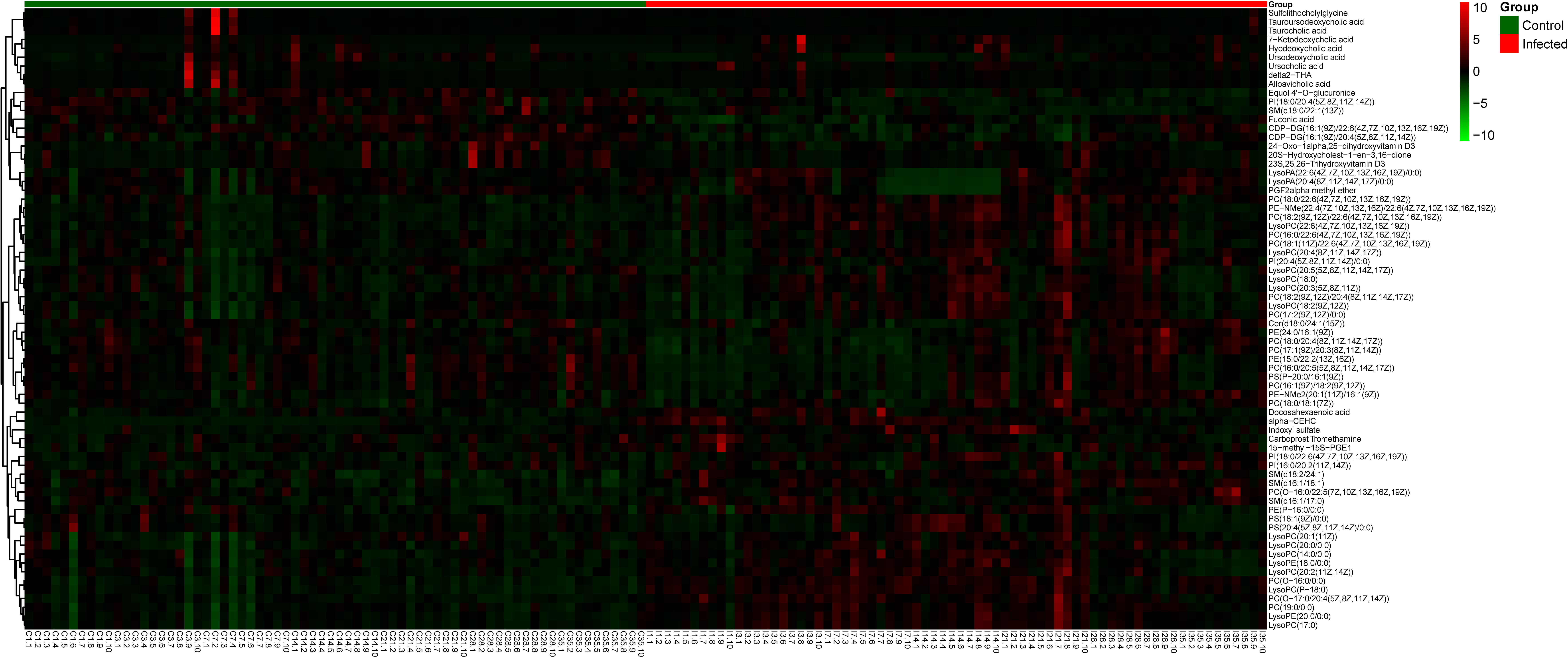

Supplement: Supplementary file 34 — Additional file34 [file 40249_2026_1436_MOESM34_ESM.tif]

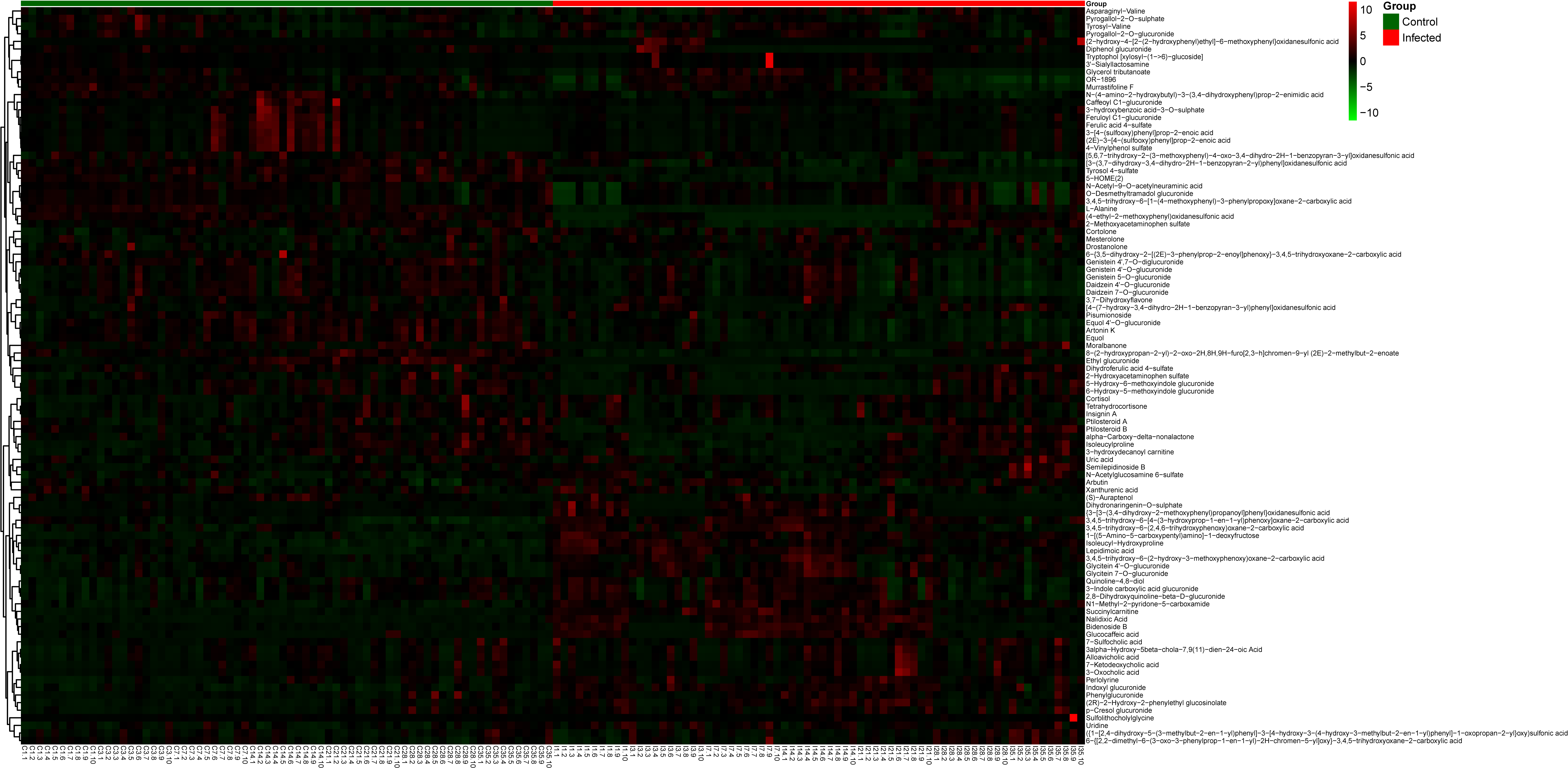

Supplement: Supplementary file 35 — Additional file35 [file 40249_2026_1436_MOESM35_ESM.tif]

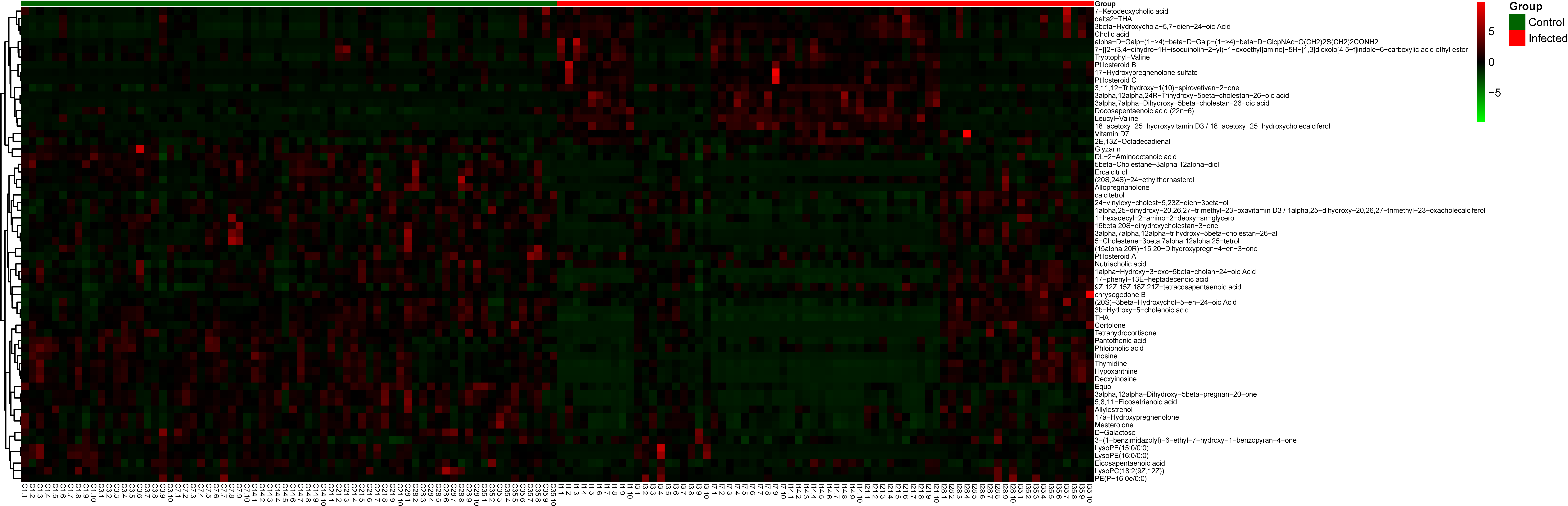

Supplement: Supplementary file 36 — Additional file36 [file 40249_2026_1436_MOESM36_ESM.tif]

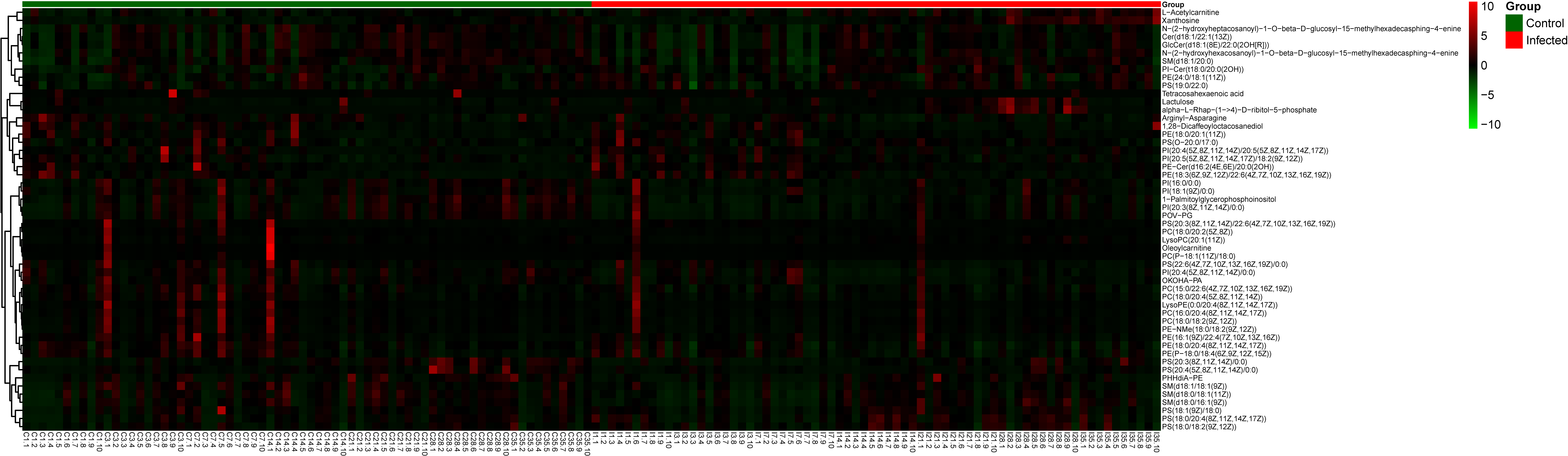

Supplement: Supplementary file 37 — Additional file37 [file 40249_2026_1436_MOESM37_ESM.tif]
