# Supplementary material for: Dynamic alterations and potential roles of gut microbiota and metabolites in Angiostrongylus cantonensis-infected mice and rats
Source: Infect Dis Poverty. 2026 Jul 2;15:74. doi: 10.1186/s40249-026-01436-7 (PMC13326367; doi:10.1186/s40249-026-01436-7)
Supplement: Supplementary file 2 — Additional file2 [file 40249_2026_1436_MOESM2_ESM.docx]

**Methods**

**Metagenome sequencing and analysis**

Adapters, low-quality bases, and reads containing ambiguous bases were initially filtered out using Trimmomatic 0.35 [1]. Subsequently, pair-end reads passing quality filter were aligned against the host genome using Burrows-Wheeler Aligner (BWA) 0.7.12 software [2], and aligned reads were discarded. Valid reads were then assembled using SOAPdenovo2 2.04 [3]. Gaps inside scaffold were used as breakpoint to interrupt the scaffold into new contigs (Scaftig), only Scaftig with a length ≥ 500 bp were retained for subsequent analysis. Open reading frames (ORFs) within the assembled scaffolds were predicted using Prodigal 2.6.3 [4], and translated into amino acid sequences. Afterwards, CDHIT 4.6.7 [5] was employed to construct non-redundant gene sets for the predicted genes across all samples. Clustering was performed at 95% sequence identity and 90% coverage, with the longest gene in each gene set selected as the representative sequence. Subsequently, clean reads from each sample were aligned against non-redundant gene set using Bowtie2 2.2.9 [6], and gene abundance in corresponding sample was calculated. Furthermore, representative sequences of gene sets were annotated with the NR database by DIAMOND 0.9.7 [7] with an e-value < 1e^-5^, yielding species level annotations and species abundance profiles. Finally, representative sequences were compared with the Kyoto Encyclopedia of Genes and Genomes (KEGG) database [8] using DIAMOND 0.9.7 [7], and information on the number and relative abundances of genes was then subjected to statistical analysis at various taxonomic levels.

**Ultraperformance liquid chromatography (UPLC) conditions for untargeted metabolomics**

Serum and brain samples were analyzed using an ACQUITY UPLC HSS T3 column (2.1 × 100 mm, 1.8 μm; Waters, Milford, MA, USA) with an injection volume of 2.500 μl. Mobile phase A was water, mobile phase B was a mixture of 70% isopropanol and 30% acetonitrile, both containing 0.1% formic acid. Separation was achieved using gradient elution at a flow rate of 350 μl/min. The gradient conditions involved holding at 1% B for 1 min, increasing from 1 to 40% B over 1 min, followed by the following ramps: 40 to 45% B over 3 min, 45 to 55% B over 2 min, 55 to 85% B over 7 min, and 85 to 99% B over 6 min, and finally returning to 1% B for 2 min.

Urine samples were separated using an ACQUITY UPLC C18 BEH column (2.1 × 100 mm, 1.7 μm; Waters, Milford, MA, USA) with an injection volume of 0.600 μl. Water was mobile phase A and acetonitrile was mobile phase B, each containing 0.1% formic acid. The gradient elution was performed at a flow rate of 400 μl/min as follows: held at 1% B for 1 min, increased from 1 to 10% B over the next 3 min, held at 10% B over 1 min, ramped from 10 to 50% B over 4 min, increased from 50 to 95% B for 2 min, held at 95% B for 1.5 min, and finally decreased to 5% B, which was held for 2.5 min.

The same gradient solvent system used for urine samples was applied to feces samples. Separation was performed on an ACQUITY UPLC HSS T3 column (2.1 × 100 mm, 1.8 μm; Waters, Milford, MA, USA) with an injection volume of 0.300 μl. The separation gradient involved holding at 1% B for 1 min, ramping from 1 to 95% B over 9 min, holding 95% B for 2 min, and returning to 1% B over 3 min, at a flow rate of 400 μl/min.

Prior to sample analysis, six consecutive injections of QC samples were performed. All samples were then injected in a fully randomized order at 40 ℃ to minimize confounding batch effects. QC samples were detected after every 10 experimental samples to monitor instrument stability.

**Quadrupole-time-of-flight mass spectrometry (Q/TOF-MS) conditions for untargeted metabolomics**

A SYNAPT G2-SiHigh-Definition Mass Spectrometer with an electrospray ionization (ESI) source (Waters, Milford, MA, USA) was utilized. Nitrogen served as both the desolvation and cone gas. For serum and brain samples, the source temperature was set to 120 ℃, the capillary voltage was 2.5 kV, the cone voltage was 35 V, the desolvation gas temperature was 350 ℃, the desolvation gas flow was set at 800 L/h, the reverse cone gas flow was set at 40 L/h, and the nebulizer gas was set to 6 bar. Eluted compounds were scanned from mass/charge (*m/z*) 50 to 1300 at a rate of 1.0 s per scan for both MS mode and MS^E^ mode, with collision energy ranging from 10 to 50 eV. For urine samples, the desolvation gas flow was 750 L/h and the reverse cone gas flow was 30 L/h, other parameters were identical to serum sample conditions. The scan range was from *m/z* 50 to 1200, with collision energy set from 20 to 50 eV. Feces samples were analyzed under conditions identical to urine samples, except the collision energy was set from 10 to 50 eV. Data correction was accomplished using leucine enkephalin (*m/z* 556.2771 in positive mode and *m/z* 554.2615 in negative mode) at a concentration of 1 ng/μl and a continuous flow rate of 5 μl/min, ensuring mass accuracy in the process of data acquisition.

**Results**

**General condition of experimental animals**

Mice in the control group grew well, exhibited frequent movement, had smooth and soft hair, normal diet and defecation, and maintained a stable body weight. By day 14, *A. cantonensis*-infected mice showed reduced activity, decreased food intake and defecation, dry and ruffled fur, lethargy, and significant weight loss. By day 21, infected mice displayed symptoms including neck stiffness, ataxia, and severe emaciation. Body weight of infected mice was significantly lower than that of control mice from day 14 onward (*P <* 0.01), culminating in a *>* 20% reduction by day 21 (Additional file 3a).

Rats in the control group grew well, had frequent activity, smooth and soft fur, normal diet and defecation, and showed a gradual increase in body weight. Infected rats exhibited erect and ruffled fur and poor mental state, but showed no significant changes in activity, food intake, or defecation. The average body weight of *A. cantonensis*-infected rats showed a small but statistically significant reduction only at the final time point, day 35 (*P <* 0.05) (Additional file 3b).

**Potential relationships between bacterial genera in *A. cantonensis*-infected mice and rats**

A correlation network diagram was constructed to illustrate the relationships between bacterial genera across different time points in *A. cantonensis*-infected mice (Additional file 15). *Lachnospiraceae NK4A136 group* showed marked positive correlations with *Oscillibacter*, *Roseburia*, *Lachnoclostridium*, and *Ruminiclostridium*, but negative correlations with *Bacteroides* and *Streptococcus*. Additionally, *Bacteroides* was also negatively associated with *Lachnoclostridium*. *Streptococcus* showed negative correlation with *Alistipes*, *Roseburia*, *Ruminiclostridium*, *Intestinimonas*, and *Lachnoclostridium*, and positive correlation with *Prevotella 9* and *Rodentibacter*. Significant positive correlations were observed among *Roseburia* and *Ruminiclostridium*, *Lachnospiraceae UCG-006*, *Intestinimonas*, and *Lachnoclostridium*, whereas *Roseburia* was negatively associated with *Prevotella 9* and *Alloprevotella*. In general, genera from Bacteroidetes were predominantly negatively associated with those from Firmicutes, except for specific pairs like *Prevotella 9* and *Streptococcus*, and *Prevotellaceae UCG-001* and *Ruminococcaceae UCG-014*, suggesting a synergistic relationship between *Streptococcus*, *Ruminococcaceae UCG-014*, and Prevotellaceae, while *Roseburia* displayed an antagonistic relationship with Prevotellaceae. These correlation patterns remained consistent throughout the infection period.

In rats, correlation analysis demonstrated that *Bacteroides* exhibited positive correlations with *Parabacteroides* and [*Eubacterium*] *coprostanoligenes group* (family Ruminococcaceae), but negative correlations with *Ruminococcaceae UCG-005* and *Blautia* (phylum Firmicutes), as well as *Prevotella 1* and *Prevotella 9* (phylum Bacteroidetes). Correlation patterns for *Parabacteroides* were largely similar to those for *Bacteroides*. *Prevotella 9* was positively correlated with *Prevotellaceae UCG-001*, *Alloprevotella*, *Prevotella 1*, *Blautia*, *Ruminococcaceae UCG-014*, and *Ruminococcus 1*, but negatively correlated with *Lachnospiraceae UCG-006*, [*Eubacterium*] *coprostanoligenes group*, and *Parabacteroides*. *Prevotella 1* showed relationships similar to *Prevotella 9*. Therefore, *Bacteroides* and *Parabacteroides* appear to mutually inhibit *Prevotella 9* and *Prevotella 1*. In addition, *Prevotellaceae UCG-001* exhibited negative associations with *Romboutsia*, [*Eubacterium*] *coprostanoligenes group* (phylum Firmicutes), and *Parabacteroides* (phylum Bacteroidetes), but positive associations with *Ruminococcaceae UCG-005* and *Ruminococcus 1* (phylum Firmicutes), consistent with correlations observed for *Prevotella 9* (Additional file 16). Overall, there was a synergistic relationship between genera from Prevotellaceae and Ruminococcaceae, except for [*Eubacterium*] *coprostanoligenes group*. Similarly, inter-genera relationships remained stable throughout the infection course in rats.

**Multivariate statistical analysis of metabolite profiles**

Metabolic profiles of *A. cantonensis*-infected mice and rats were compared. First, unsupervised PCA was employed to evaluate the natural distribution of samples from mice (Additional file 18). QC samples of serum, urine, feces, and brain clustered tightly in both ESI+ and ESI- modes, confirming high data reliability. Urine samples from infected and control mice were clearly separated, whereas other sample types showed a trend toward separation but were not completely differentiated, indicating partial alterations of metabolic networks in infected mice. Supervised OPLS-DA was then performed to further elucidate differences between infected and control groups (Additional files 19-22). Results showed complete separation between infected and control groups for serum, urine, feces, and brain samples across different time points under both ESI modes, highlighting marked differences in metabolic phenotypes. Moreover, high R^2^Y and Q^2^ values for each model affirmed their stability, reliability, and ability to effectively explain and predict intergroup differences (Additional file 23).

In PCA plots for rat samples, QC samples for serum, urine, feces, and brain clustered clearly in both ESI+ and ESI- modes, indicating data suitability for subsequent analysis. Clear differentiation between infected and control groups was not evident for serum and brain samples, while urine and feces samples showed trends of separation with some overlap (Additional file 24), suggesting metabolic alterations in infected rats were less pronounced than in infected mice.

Subsequently, OPLS-DA was applied to identify group differences (Additional files 25-28), and the results exhibited that all sample types from all groups at the seven time points could be completely differentiated, with high R^2^Y and Q^2^ values in both positive and negative ion modes (Additional file 29), indicating marked alterations in the metabolic phenotypes of infected rats.

**References**

1. Bolger AM, Lohse M, Usadel B. Trimmomatic: a flexible trimmer for Illumina sequence data. Bioinformatics. 2014;30(15):2114–20.

2. Li H, Durbin R. Fast and accurate long-read alignment with Burrows-Wheeler transform. Bioinformatics. 2010;26(5):589–95.

3. Luo R, Liu B, Xie Y, Li Z, Huang W, Yuan J, et al. SOAPdenovo2: an empirically improved memory-efficient short-read de novo assembler. Gigascience. 2012;1(1):18.

4. Hyatt D, Chen GL, Locascio PF, Land ML, Larimer FW, Hauser LJ. Prodigal: prokaryotic gene recognition and translation initiation site identification. BMC Bioinform. 2010;11:119.

5. Li W, Jaroszewski L, Godzik A. Clustering of highly homologous sequences to reduce the size of large protein databases. Bioinformatics. 2001;17(3):282–3.

6. Langmead B, Salzberg SL. Fast gapped-read alignment with Bowtie 2. Nat Methods. 2012;9(4):357–9.

7. Buchfink B, Xie C, Huson DH. Fast and sensitive protein alignment using DIAMOND. Nat Methods. 2015;12(1):59–60.

8. Kanehisa M, Goto S, Sato Y, Kawashima M, Furumichi M, Tanabe M. Data, information, knowledge and principle: back to metabolism in KEGG. Nucleic Acids Res. 2014;42:D199–205.
