# Supplementary material for: Dynamic alterations and potential roles of gut microbiota and metabolites in Angiostrongylus cantonensis-infected mice and rats
Source: Infect Dis Poverty. 2026 Jul 2;15:74. doi: 10.1186/s40249-026-01436-7 (PMC13326367; doi:10.1186/s40249-026-01436-7)
Supplement: Supplementary file 23 — Additional file23 [file 40249_2026_1436_MOESM23_ESM.docx]

| Samples | Modes | Day | Perm R^2^Y | *P*-value | Perm Q^2^ | *P*-value |
| --- | --- | --- | --- | --- | --- | --- |
| Serum | ESI+ | 1 | 0.994 | 0.029 | 0.766 | 0.001 |
|  |  | 3 | 0.893 | 0.60 | 0.515 | 0.002 |
|  |  | 7 | 0.86 | 0.198 | 0.379 | 0.017 |
|  |  | 14 | 0.966 | 0.03 | 0.385 | 0.022 |
|  |  | 21 | 0.998 | < 0.001 | 0.874 | < 0.001 |
|  | ESI- | 1 | 0.997 | 0.002 | 0.759 | < 0.001 |
|  |  | 3 | 0.993 | 0.035 | 0.808 | < 0.001 |
|  |  | 7 | 0.995 | 0.023 | 0.818 | < 0.001 |
|  |  | 14 | 0.992 | 0.039 | 0.819 | < 0.001 |
|  |  | 21 | 0.97 | 0.004 | 0.817 | < 0.001 |
| Urine | ESI+ | 1 | 0.995 | < 0.001 | 0.871 | < 0.001 |
|  |  | 3 | 0.995 | < 0.001 | 0.887 | < 0.001 |
|  |  | 7 | 0.99 | < 0.001 | 0.901 | < 0.001 |
|  |  | 14 | 0.997 | < 0.001 | 0.942 | < 0.001 |
|  |  | 21 | 0.997 | < 0.001 | 0.923 | < 0.001 |
|  | ESI- | 1 | 0.987 | < 0.001 | 0.942 | < 0.001 |
|  |  | 3 | 0.989 | < 0.001 | 0.892 | < 0.001 |
|  |  | 7 | 0.993 | < 0.001 | 0.95 | < 0.001 |
|  |  | 14 | 0.994 | < 0.001 | 0.964 | < 0.001 |
|  |  | 21 | 0.994 | < 0.001 | 0.95 | < 0.001 |
| Feces | ESI+ | 1 | 0.995 | < 0.001 | 0.929 | < 0.001 |
|  |  | 3 | 0.96 | < 0.001 | 0.919 | < 0.001 |
|  |  | 7 | 0.996 | < 0.001 | 0.949 | < 0.001 |
|  |  | 14 | 0.982 | < 0.001 | 0.912 | < 0.001 |
|  |  | 21 | 0.989 | < 0.001 | 0.92 | < 0.001 |
|  | ESI- | 1 | 0.997 | 0.001 | 0.932 | 0.001 |
|  |  | 3 | 0.997 | < 0.001 | 0.921 | < 0.001 |
|  |  | 7 | 0.985 | < 0.001 | 0.924 | < 0.001 |
|  |  | 14 | 0.996 | < 0.001 | 0.93 | < 0.001 |
|  |  | 21 | 0.989 | < 0.001 | 0.89 | < 0.001 |
| Brain | ESI+ | 1 | 0.988 | 0.001 | 0.541 | 0.001 |
|  |  | 3 | 0.985 | 0.003 | 0.511 | 0.004 |
|  |  | 7 | 0.978 | 0.004 | 0.69 | < 0.001 |
|  |  | 14 | 0.991 | 0.003 | 0.494 | 0.01 |
|  |  | 21 | 0.993 | 0.017 | 0.572 | 0.001 |
|  | ESI- | 1 | 0.995 | 0.116 | 0.375 | 0.031 |
|  |  | 3 | 0.991 | 0.403 | 0.4 | 0.018 |
|  |  | 7 | 0.997 | 0.005 | 0.313 | 0.066 |
|  |  | 14 | 0.999 | 0.156 | 0.278 | 0.2 |
|  |  | 21 | 0.997 | 0.005 | 0.294 | 0.09 |
