# Supplementary material for: Dynamic alterations and potential roles of gut microbiota and metabolites in Angiostrongylus cantonensis-infected mice and rats
Source: Infect Dis Poverty. 2026 Jul 2;15:74. doi: 10.1186/s40249-026-01436-7 (PMC13326367; doi:10.1186/s40249-026-01436-7)
Supplement: Supplementary file 29 — Additional file29 [file 40249_2026_1436_MOESM29_ESM.docx]

| Samples | Modes | Day | Perm R^2^Y | *P*-value | Perm Q^2^ | *P*-value |
| --- | --- | --- | --- | --- | --- | --- |
| Serum | ESI+ | 1 | 0.96 | < 0.001 | 0.887 | < 0.001 |
|  |  | 3 | 0.994 | < 0.001 | 0.856 | < 0.001 |
|  |  | 7 | 0.985 | < 0.001 | 0.882 | < 0.001 |
|  |  | 14 | 0.981 | < 0.001 | 0.861 | < 0.001 |
|  |  | 21 | 0.994 | 0.004 | 0.729 | < 0.001 |
|  |  | 28 | 0.996 | 0.003 | 0.762 | < 0.001 |
|  |  | 35 | 0.994 | 0.11 | 0.739 | < 0.001 |
|  | ESI- | 1 | 0.991 | < 0.001 | 0.917 | < 0.001 |
|  |  | 3 | 0.946 | 0.001 | 0.787 | < 0.001 |
|  |  | 7 | 0.988 | < 0.001 | 0.882 | < 0.001 |
|  |  | 14 | 0.998 | < 0.001 | 0.873 | < 0.001 |
|  |  | 21 | 0.998 | 0.005 | 0.835 | < 0.001 |
|  |  | 28 | 0.988 | 0.036 | 0.675 | 0.001 |
|  |  | 35 | 0.986 | < 0.001 | 0.759 | < 0.001 |
| Urine | ESI+ | 1 | 0.991 | < 0.001 | 0.923 | < 0.001 |
|  |  | 3 | 0.996 | < 0.001 | 0.888 | < 0.001 |
|  |  | 7 | 0.992 | < 0.001 | 0.927 | < 0.001 |
|  |  | 14 | 0.994 | < 0.001 | 0.937 | < 0.001 |
|  |  | 21 | 0.992 | < 0.001 | 0.85 | < 0.001 |
|  |  | 28 | 0.992 | < 0.001 | 0.923 | < 0.001 |
|  |  | 35 | 0.968 | < 0.001 | 0.718 | < 0.001 |
|  | ESI- | 1 | 0.997 | < 0.001 | 0.93 | < 0.001 |
|  |  | 3 | 0.997 | < 0.001 | 0.797 | < 0.001 |
|  |  | 7 | 0.992 | < 0.001 | 0.88 | < 0.001 |
|  |  | 14 | 0.996 | < 0.001 | 0.912 | < 0.001 |
|  |  | 21 | 0.996 | 0.001 | 0.868 | < 0.001 |
|  |  | 28 | 0.998 | < 0.001 | 0.889 | < 0.001 |
|  |  | 35 | 0.985 | 0.199 | 0.706 | < 0.001 |
| Feces | ESI+ | 1 | 0.986 | < 0.001 | 0.923 | < 0.001 |
|  |  | 3 | 0.994 | < 0.001 | 0.813 | < 0.001 |
|  |  | 7 | 0.996 | < 0.001 | 0.974 | < 0.001 |
|  |  | 14 | 0.997 | < 0.001 | 0.958 | < 0.001 |
|  |  | 21 | 0.994 | < 0.001 | 0.952 | < 0.001 |
|  |  | 28 | 0.996 | < 0.001 | 0.887 | < 0.001 |
|  |  | 35 | 0.946 | 0.023 | 0.735 | < 0.001 |
|  | ESI- | 1 | 0.996 | < 0.001 | 0.937 | < 0.001 |
|  |  | 3 | 0.996 | < 0.001 | 0.843 | < 0.001 |
|  |  | 7 | 0.998 | < 0.001 | 0.971 | < 0.001 |
|  |  | 14 | 0.997 | < 0.001 | 0.961 | < 0.001 |
|  |  | 21 | 0.996 | < 0.001 | 0.938 | < 0.001 |
|  |  | 28 | 0.989 | < 0.001 | 0.836 | < 0.001 |
|  |  | 35 | 0.975 | 0.001 | 0.755 | < 0.001 |
| Brain | ESI+ | 1 | 0.954 | 0.194 | 0.263 | 0.089 |
|  |  | 3 | 0.829 | 0.285 | 0.393 | 0.01 |
|  |  | 7 | 0.878 | 0.029 | 0.189 | 0.085 |
|  |  | 14 | 0.986 | 0.014 | 0.378 | 0.034 |
|  |  | 21 | 0.986 | 0.074 | 0.543 | 0.005 |
|  |  | 28 | 0.996 | 0.086 | 0.498 | 0.012 |
|  |  | 35 | 0.995 | 0.064 | 0.363 | 0.085 |
|  | ESI- | 1 | 0.987 | 0.069 | 0.227 | 0.139 |
|  |  | 3 | 0.719 | 0.906 | 0.177 | 0.13 |
|  |  | 7 | 0.869 | 0.066 | 0.0615 | 0.215 |
|  |  | 14 | 0.99 | 0.006 | 0.357 | 0.042 |
|  |  | 21 | 0.996 | 0.186 | 0.574 | 0.004 |
|  |  | 28 | 0.901 | 0.079 | 0.511 | 0.001 |
|  |  | 35 | 0.977 | 0.064 | 0.322 | 0.077 |
